# Association between human herpesviruses infections and childhood neurodevelopmental disorders: insights from two-sample mendelian randomization analyses and systematic review with meta-analysis

**DOI:** 10.1186/s13052-024-01820-9

**Published:** 2024-11-20

**Authors:** Liwei Fang, Zuojun Wang, Jingyi Zhao, Xun Wu, Shunxin Wang, Hui Gao, De Wu

**Affiliations:** 1 Pediatric Neurorehabilitation Center, Pediatric Department, The First Affiliated Hospital of Anhui Medical University, Hefei, China; 2 Key Laboratory of Population Health Across Life Cycle (Anhui Medical University), Ministry of Education of the People’s Republic of China, Hefei, Anhui China

**Keywords:** Neurodevelopmental disorders, Human herpesviruses, Mendelian randomization, Genome-wide association study, Systematic review

## Abstract

**Background:**

The potential roles of viral infections in neurodevelopmental disorders (NDDs) have been suggested based on previous studies. Given the high prevalence of human herpesviruses (HHVs), the associations between HHVs infection and the risk of NDDs warrant explored.

**Methods:**

Our study employs a two-sample Mendelian randomization (MR) analysis and systematic review with meta-analysis to investigate whether genetically predicted HHVs infection are linked to three main childhood NDDs—autism spectrum disorder (ASD), attention deficit hyperactivity disorder (ADHD), and Tourette syndrome (TS). We utilized genetic variants associated with HHV infections in genome-wide association study (GWAS) summary datasets of European populations to establish instrumental variables and statistics for three NDDs obtained from Psychiatric Genomics Consortium. MR analysis was performed using inverse-variance weighted, MR Egger, weighted median, simple median, weighted mode, and MR-PRESSO. In addition, publications associating HHVs infection with three NDDs were systematically searched using PubMed, Web of Science, and three Chinese databases for meta-analyses.

**Results:**

The MR results found no evidence to support a link between genetically predicted HHVs infection and the risk of NDDs based on existing datasets. Twenty-seven observational studies on children with HHVs infection and NDDs were considered eligible. Meta-analysis showed that cytomegalovirus and HHV-6 infection were related with ASD, while Epstein-Barr virus and cytomegalovirus infection were associated with TD in Chinese population. **Conclusions:** These results contribute to a comprehensive understanding of the possibilities underlying HHV infections in affecting childhood NDDs. Further research is necessary to include larger and more robust statistics of HHV infections and NDDs.

**Trial registration:**

This systematic review was registered at PROSPERO as CRD42024554169. Retrospectively registered 26 July 2024.

**Supplementary Information:**

The online version contains supplementary material available at 10.1186/s13052-024-01820-9.

## Background

Neurodevelopmental disorders (NDDs) are a group of disorders affecting the development of the central nervous system (CNS) in children, including autism spectrum disorder (ASD), attention deficit hyperactivity disorder (ADHD) and tic disorders [1, 2]. ASD manifests through impaired social interaction, alongside restrictive and repetitive behaviors or interests. In 2018, the estimated prevalence of ASD among 8-year-old children in the United States was 2.3% [3], which increased to approximately 2.8% in 2020 [4]. ADHD, characterized by symptoms of inattention, hyperactivity, and impulsivity, stands as the most prevalent mental disorder affecting children and adolescents globally, with prevalence estimated at 6–7% [5]. Tourette syndrome (TS), as one kind of tic disorders, includes both phonic and motor tics, showed an estimated prevalence of 0.3% to 0.9% among school-age children aged 4–18 years [6]. These disorders could impact children's cognition, language, movement, and learning abilities, with some impairments even exist for a lifetime, resulting in health and social challenges [7, 8]. However, the etiology of NDDs is multifaceted and elusive, with genetic, neurological, and environmental factors likely contributing in combination. Perinatal infection is one of the potential risk factors for childhood NDDs [9–13]. Viral infections, which are common in humans, have been highlighted in previous studies for their correlations with ASD [14–16], ADHD [17, 18], and TS [19, 20].

Human herpesviruses (HHVs) are a group of double-stranded DNA viruses with the capacity to infect humans, comprising eight species categorized into three families. Alpha (α)-herpesviruses primarily reside in neurons, including HHV-1 and HHV-2—herpes simplex virus (HSV) 1 and 2, as well as HHV-3—varicella-zoster virus (VZV); beta (β)-herpesviruses encompass HHV-5—cytomegalovirus (CMV), HHV-6, and HHV-7, which persist in macrophages and lymphocytes; gamma (γ)-herpesviruses exclusively inhabit lymphocytes, including HHV-4—Epstein-Barr virus (EBV) and HHV-8 [21].

HHVs can be transmitted through contact and often result in latent infections, leading to a high prevalence but relatively low incidence in the population. Data from 2016 indicate that the global prevalence of HSV-1 among individuals aged 0–49 is approximately 67%, with an incidence rate of about 2%. For HSV-2, the prevalence is around 13%, while the incidence rate is only 0.6% [22]. The global burden of VZV infection in 2019 exceeded 80 million cases, with the highest disease burden observed among children under 5 years of age [23]. CMV demonstrates a global seroprevalence estimated at 83% in general population, reaching as high as 86% in the women of reproductive age [24]. The overall prevalence of congenital CMV (cCMV) infection in newborns is approximately 0.6% [25]. EBV infection affects over 90% of the global population, with the majority of children becoming seropositive by the age of 5 [26]. Furthermore, HHV-6 infects over 90% of infants and commonly persists throughout life [27], while HHV-7, closely related to HHV-6, similarly induces infections during childhood [28]. Given that HHVs can affect the fetus through perinatal infection or directly infect children, their potential association with neurodevelopment is of significant interest.

Mendelian randomization (MR) analysis follows Mendel's law of random distribution of parental alleles to offspring, serving as a method to infer causal relationships between phenotypes and diseases by utilizing genetic variants as instrumental variables (IVs) [29]. It is also referred to as "nature's randomized controlled trial." MR studies are less susceptible to reverse causation and confounding factors compared to observational studies [30]. In the absence of randomized controlled trials, MR holds the highest level of evidence in the hierarchy of evidence-based medicine [31]. In recent years, the emergence of genome-wide association studies (GWAS) has revealed associations between single nucleotide polymorphisms (SNPs) and diseases in specific populations [32]. This provides an excellent opportunity to use MR methods to investigate the causal effects of viral infections on NDDs.

This study, based on publicly available GWAS data, employs a two-sample MR approach to explore whether there are causal associations between HHVs and ASD, ADHD, and TS. Additionally, we conducted a systematic review of observational studies and performed selective meta-analyses to provide relevant evidence.

## Methods

### MR analysis

#### Study design

**Fig. 1:**
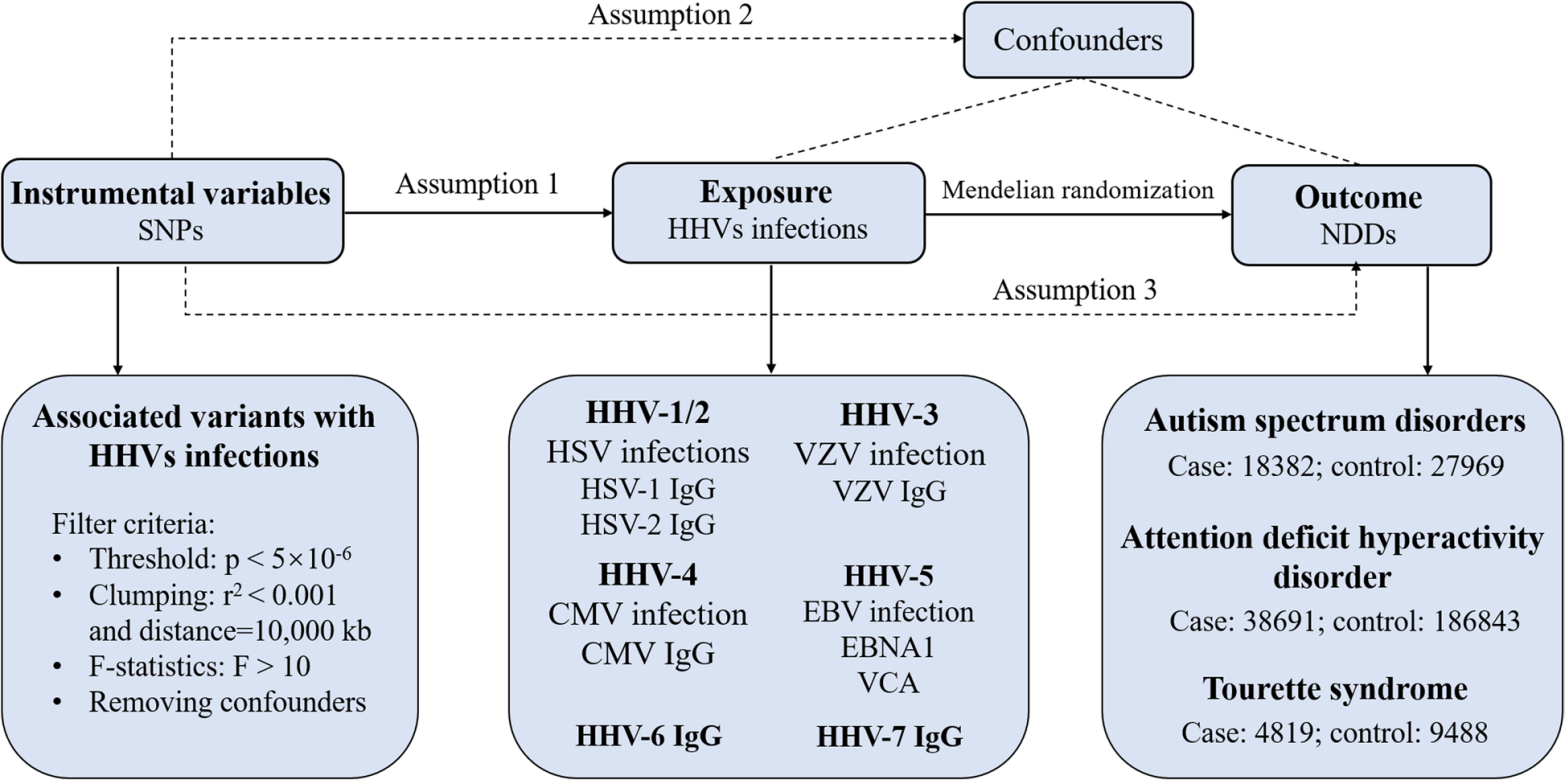
The flow chart of two-sample Mendelian randomization study design on HHV infections and three childhood NDDs. All GWAS summary data were obtained from participants of European ancestry

A two-sample MR analysis method was employed to investigate the potential relationship between HHVs and three main child NDDs (ASD, ADHD, and TS). To ensure the robustness of our findings, our MR study adhered to three fundamental assumptions: 1. Genetic variants exhibit significant associations with circulating cytokine levels (association hypothesis); 2. Genetic variants remain independent of other confounding factors (independence assumption); 3. Genetic variants should exclusively influence outcomes through exposure factors (exclusivity hypothesis) (Fig. 1). We adhered to the Strengthening the Reporting of Observational Studies in Epidemiology using Mendelian Randomization (STROBE-MR) guidelines to ensure methodological rigor in the study, as shown in Additional file 1: STROBE-MR checklist.

### Data sources

We obtained summary data on genetic variants related to HHVs as exposures from the latest FinnGen (https://r10.finngen.fi/) and the IEU Open GWAS project (https://gwas.mrcieu.ac.uk/). Since no published data could be found for HHV-8, only GWASs for HHV1-7 were included in this study. Datasets were listed as followings: HSV infection: finngen_R10_AB1_HERPES_SIMPLEX (3,723 cases and 396,378 controls), finngen_R10_H7_HERPESKERATITIS (1,252 cases and 390,647 controls), and finngen_R10_AB1_ANOGENITAL_HERPES_SIMPLEX (1,986 cases and 400,197 controls); HSV-1 IgG: ebi-a-GCST006346 (645 cases), and HSV-2 IgG: ebi-a-GCST006347 (208 cases). VZV infection: finngen_R10_AB1_ZOSTER (5,488 cases and 396,478 controls); VZV IgG: ebi-a-GCST90006928 (8735 cases). EBV infection: finngen_R10_AB1_EBV (2,979 cases and 400,974 controls); EBNA1: ebi-a-GCST006361 (914 cases); VCA: ebi-a-GCST006362 (956 cases). CMV infection: finngen_R10_CMV_NOS (487 cases and 411,593 controls); CMV IgG: ieu-b-4900 (5,010 cases). HHV-6 IgG: ebi-a-GCST90006902 (8,735 cases). HHV-7 IgG: ebi-a-GCST90006908 (8,735 cases).

GWAS summary statistics for outcomes including ASD (18,381 cases and 27,969 controls) [33], ADHD (38,691 cases and 186,843 controls) [34], and TS (4,819 and 9,488 controls) [35] were downloaded from the Psychiatric Genomics Consortium (PGC) (https://pgc.unc.edu/for-researchers/download-results/). All GWASs were based on European populations.

### Instruments selection

Initially, we applied a stringent genome-wide significance threshold (*p* < 5 × 10^−8^) to select highly correlated IVs. However, due to the limited number of SNPs meeting this criterion for most exposures, a more permissive significance threshold (*p* < 5 × 10^−6^) was adopted. Then we performed clumping (r^2^ < 0.001 and distance = 10,000 kb) to eliminate IVs exhibiting linkage disequilibrium. F-statistics were used to determine the strength of the relationship between selected IVs and exposure. When F > 10, IVs are considered strong instrumental variables. The F-statistic is calculated by *F* = *R*^*2*^*(N − 2)/(1 − R*^*2*^*)* and *R*^*2*^ = *2* × *EAF* × *(1 − EAF)* × *beta*^*2*^, where R^2^ is the proportion of exposure variability explained by each instrument and N is the sample size of the GWAS for the SNP-HHVs relationship [36]. Details of all SNPs clumped and the F-statistic and R^2^ are provided in Additional file 1: Table S1. Subsequently, a harmonization process was implemented, and palindromic SNPs were removed. We employed GWAS catalog (https://www.ebi.ac.uk/gwas/) to detect other phenotypes of individual SNPs to eliminate potential influence on the results.

### Statistical analysis

A total of five main MR analysis methods namely inverse-variance weighted (IVW), MR Egger, weighted median, simple median, and weighted mode were used in this study. These analyses are only conducted when the number of IVs ≥ 3. The IVW method was designated as the primary analytical tool for deriving credible causal effect estimates [37]. Evaluation of potential pleiotropy and heterogeneity involved MR-Egger intercept, MR-PRESSO method, and Cochran's Q test. Absence of vertical pleiotropy was determined if the MR-Egger intercept did not significantly deviate from 0 [38]. The MR-PRESSO results can be used to determine the presence of outliers, and *P* > 0.05 indicates the absence of horizontal pleiotropy [39]. Heterogeneity, as assessed by Cochran's Q statistic, was considered non-existent when *p* > 0.05, leading to the adoption of the fixed-effects IVW model as the primary outcome. Additionally, a leave-one-out analysis was performed to estimate the impact of individual aberrant SNPs on the overall MR analysis, ensuring result robustness. A causal relationship was deemed statistically significant if the results of MR analysis meet the following three conditions: (1) the p value of the IVW method was less than corrected *p*-value for multiple testing; (2) consistency between IVW and other methods was observed; (3) no pleiotropy and heterogeneity were identified. While *p* < 0.05 was considered suggestively causal significant. All statistical analyses were conducted using the “TwoSampleMR” and “MendelianRandomisation” package in R version 4.3.0.

### Systematic review with *meta*-analysis

#### Literature Search

**Fig. 2:**
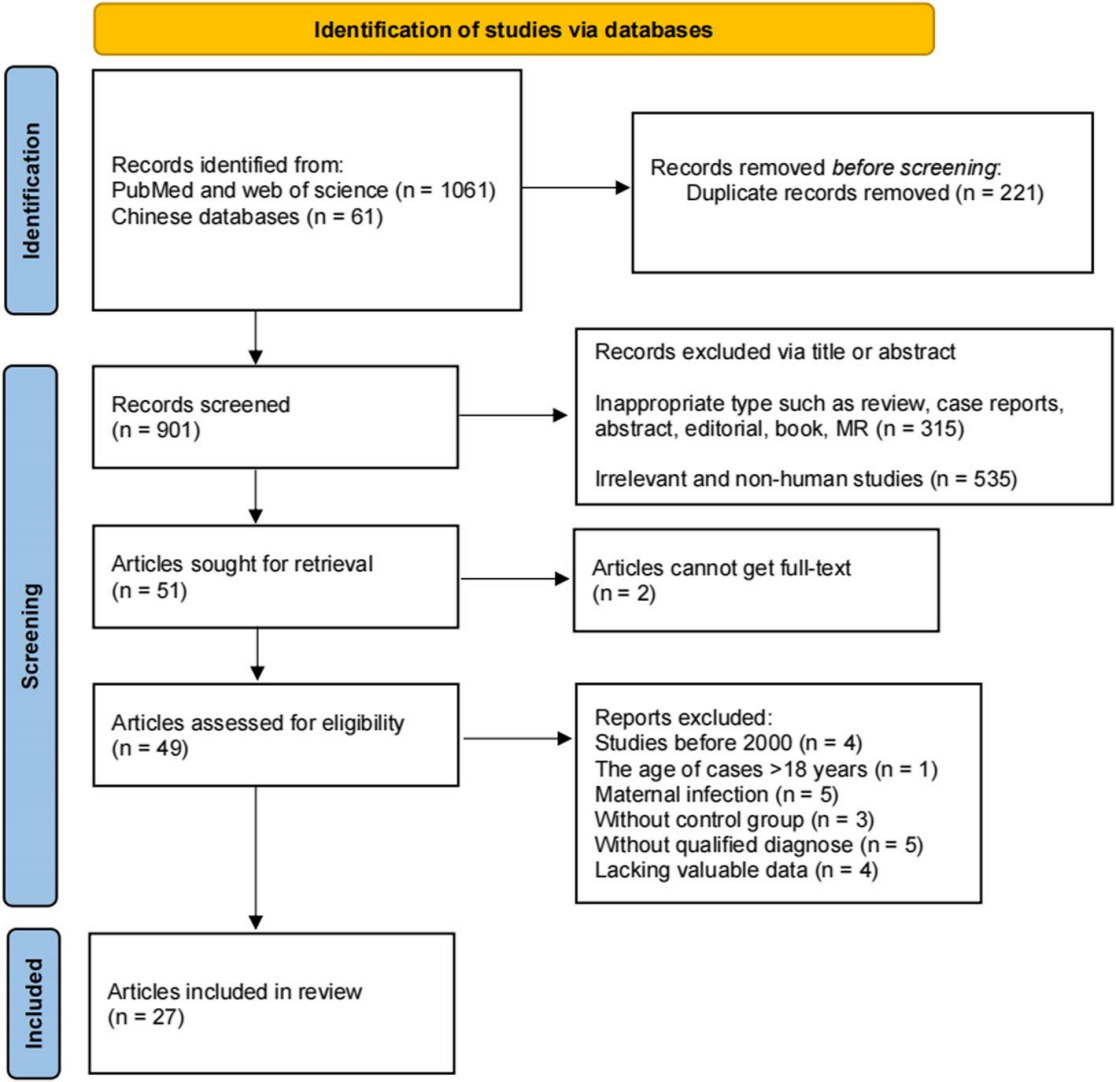
PRISMA (Preferred Reporting Items for Systematic Reviews and Meta-Analyses) flow diagram for study selection for the systematic review on HHV infections and three childhood NDDs

This part of study was reported according to the Preferred Reporting Items for Systematic reviews and Meta-Analyses (PRISMA) guideline [40], and the checklist was provided in Additional file 4: PRISMA checklist. The study protocol is also registered at PROSPERO (CRD42024554169). Five databases including PubMed (Medline), Web of Science, China National Knowledge Infrastructure, Wanfang Data Knowledge Service Platform, and China Science and Technology Journal Database—were systematically searched from inception to 1 June 2024. To search relevant studies of HHV infection and NDD, MeSH terms of all kinds of HHVs, ASD, ADHD, and TS were used. Detailed information was listed in Addition file 4: Search terms and strategy. We also manually reviewed the references of retrieved studies to identify additional studies. The flowchart of literature search was displayed in Fig. 2.

#### Inclusion and exclusion criteria

Inclusion: 1) studies comparing the prevalence of HHV infection between control groups and cases of NDDs, or comparing the prevalence of NDD between control groups and cases of HHV infection; 2) observational studies published after the year 2000; 3) NDD diagnoses based on established criteria, including Diagnostic and statistical manual of mental disorders (DSM), International Classification of Diseases (ICD), or assessments by qualified medical professionals; 4) detection of HHV infection indices, including antibodies or DNA, through body specimens such as blood, urine, or brain samples. Exclusion: 1) studies involving non-human subjects, case reports, or reviews lacking relevant information; 2) studies lacking valuable data or not providing full-text access.

### Study selection and risk of bias assessment

After removing the duplicate publications using Endnote (version X9, Clarivate Analytics), two researchers (LF and ZW) independently screened the titles and abstracts of the literatures for eligibility and selected qualified studies based on search strategy and inclusion criteria. Any discrepancies were dissolved through discussion with a third researcher (JZ).

The Agency for Healthcare Research and Quality (AHRQ) evaluation criteria was used to evaluate the risk of bias in the observational studies [41]. The AHRQ criteria consist of 11 items, each of which is answered as "yes," "no," or "not reported." A "yes" response scores 1 point, while "no" and "not reported" responses score 0 points. Scores ranging from 0 to 3 are considered low quality, 4 to 6 are considered medium quality, and 8 to 11 are considered high quality. The specific assessments were provided in Additional file 4: Risk of bias assessments.

### Data extraction and quality assessment

Data were extracted based on the PECOS (Population, Exposure, Comparator, Outcomes and Study design) framework. Bibliographic information includes author's name, year of publication. Background information includes study design, country, sample source, detection methods, sample collecting time, population size, number of positive in case and control groups, and statistical methods.

Then we used the Newcastle–Ottawa Rating Scale (NOS) to evaluate the quality of the observational studies [42]. Two researchers (XW and SW) followed the three evaluation parameters of NOS including selection, comparability, and outcome to give the “asterisk score” of each study. The score ranged from 1 to 9: 1–3, 4–6, and 7–9, which are considered low, medium, and high quality, respectively. The specific assessments were provided in Additional file 4: Quality assessments.

### Statistical analysis

Meta-analysis was considered when at least two studies assessed the same HHV infection as a risk factor. The pooled odds ratio (pOR) was calculated with 95% CI using the size and number of HHV-positive infections in NDDs patients obtained from observational studies to assess the risk of HHV infection on NDDs by random effects models or fixed effects models. The Cochran's Q test was used to assess statistical heterogeneity between studies, and I^2^ statistics were used for quantification. The fixed-effects model of I^2^ is less than 50% and the random-effects model of I^2^ is more than 50%. Furthermore, Sensitivity analysis was conducted using the leave-one-out method. Subgroup analysis was performed on detection method, sample collection time, sample type, and geographic area when possible. A combination of a visual inspection of the funnel plot, and the Begg's test (number of studies ≥ 10) was used to investigate the existence and impact of the publication bias. Statistical analyses were conducted using the “meta” package in R version 4.3.0.

## Results

### MR analysis

In summary, 14 HHV-related GWASs and 3 GWASs of ASD, ADHD, and TS were included. After clumping and excluding weak SNPs with F-statistics < 10, SNPs from 7 HHV-related GWASs were included as IVs. Among these, 12 genetic variants were used as IVs for HSV infection, 4 genetic variants for herpes simplex keratitis and keratoconjunctivitis, 5 genetic variants for HSV genital infection, 14 genetic variants for herpes zoster, 16 genetic variants for EBV infection, 5 variants for unspecified cytomegalovirus diseases, and 11 variants for anti-CMV IgG levels (Table 1).

**Table 1:** Selection of robust instrumental variables in the GWASs of HHV infections

Exposure	Num of snps under p thershold = 5e-8	Num of snps under p thershold = 5e-6	Num of snps after clumping	Num of snps (F > 10)
**HHV-1/2**				
HSV infections	2	103	12	12
HSV keratitis and keratoconjunctivitis	0	54	4	4
HSV anogenital infection	0	78	5	5
HSV-1 IgG	0	11	4	0
HSV-2 IgG	0	28	8	0
**HHV-3**				
Herpes zoster	141	925	14	14
VZV IgG	1	2531	18	0
**HHV-4**				
EBV infections	11	232	16	16
EBNA1	2	442	8	2
VCA	0	42	6	0
**HHV-5**				
CMV infections	0	63	5	5
CMV IgG	0	35	11	11
**HHV-6/7**				
HHV-6 IgG	0	66	16	0
HHV-7 IgG	0	31	27	0

*Note*: Clumping under r^2^ < 0.001 and distance = 10,000 kb

Subsequently, a harmonization process and cofounders searching were implemented. After removal of palindromic SNPs and potential confounders, there were only 1 or 2 IVs associated with HSV keratitis and keratoconjunctivitis, as well as HSV anogenital infection. Therefore, 5 exposures related to HHV infection were finally included. After correction for multiple testing (5 exposures × 3 outcomes = 15 tests), p value was calculated as *p* = 0.05/15 = 3.3 × 10^−3^. The harmonized SNPs are provided in Additional file 1: Table S2 and the potential traits of confounders are listed in Additional file 1: Table S3.

Through preliminary MR analysis and sensitivity analysis, some outliers were found by leave-one-out analysis (HSV infection with ASD: rs4716482, rs2004786; zoster with ASD: rs8181185, rs13313427, rs81302; EBV with ASD: rs28529232, rs59257919, rs318497, rs112242506, rs10468923; EBV with TS: rs59257919, rs12358176, rs2618374; CMV infection with ASD: rs61825717; CMV infection with ADHD: rs13156302; CMV infection with TS: rs13156302; CMV IgG with ASD: rs7761068, rs12214648, rs146990284).

**Fig. 3:**
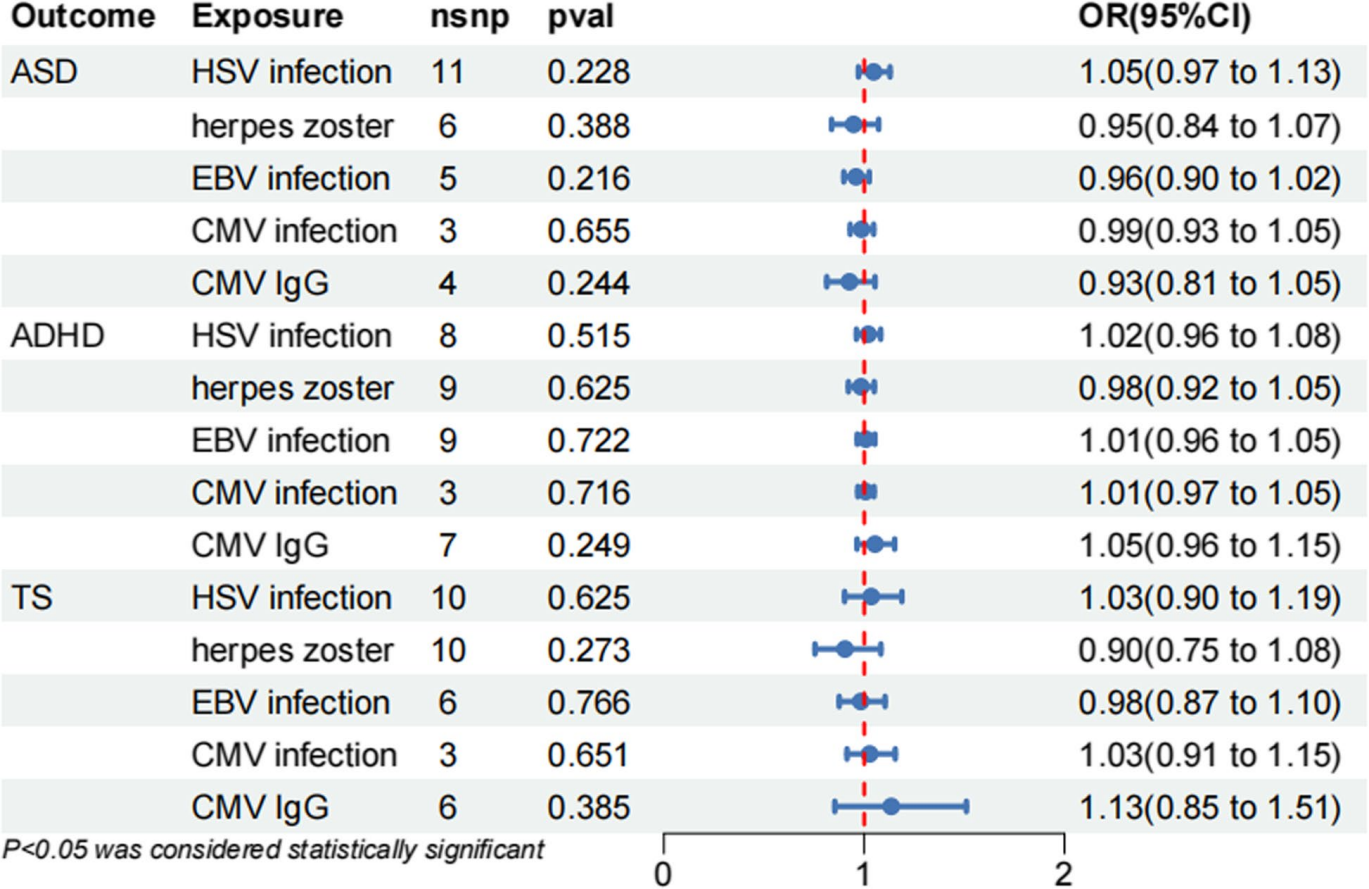
Forest plot of inverse-variance weighted analyses for the associations between five HHV-related exposures and ASD, ADHD, and TS. *P* < 0.05 is considered suggestively causal significant. nsnp: number of single-nucleotide polymorphism; OR: odd ratio; CI: confidence interval

We reanalyzed the association after removed these outliers, and the results of IVW analysis were presented in a forest plot (Fig. 3). The detailed summary effect estimates for relationships between IVs and outcomes using all MR methods are provided in Table 2. All MR methods indicate no significant causal relationship between HSV, VZV, EBV, and CMV infections and ASD, ADHD, and TS.

**Table 2:** Effect estimates for association of HHV infections with ASD, ADHD, and TS by MR

Exposure	Outcome	SNP	Method	OR	95%CI	*p*-value	Q	Q-p
HSV infection	ASD	11	IVW	1.047	0.971–1.129	0.228	10.786	0.374
			MR Egger	0.993	0.822–1.199	0.941	10.357	0.322
			Simple mode	0.980	0.833–1.153	0.815		
			Weighted median	1.009	0.909–1.120	0.869		
			Weighted mode	0.977	0.831–1.149	0.782		
			MR-PRESSO			0.255		
			MR Egger-intercept			0.556		
	ADHD	8	IVW	1.020	0.961–1.082	0.515	4.820	0.445
			MR Egger	1.020	0.889–1.170	0.790	4.820	0.682
			Simple mode	1.073	0.952–1.209	0.284		
			Weighted median	1.031	0.949–1.120	0.470		
			Weighted mode	1.061	0.951–1.182	0.323		
			MR-PRESSO			0.458		
			MR Egger-intercept			0.999		
	TS	10	IVW	1.035	0.902–1.188	0.625	3.672	0.932
			MR Egger	1.068	0.784–1.455	0.687	3.622	0.890
			Simple mode	1.127	0.855–1.487	0.418		
			Weighted median	1.064	0.882–1.283	0.425		
			Weighted mode	1.118	0.860–1.454	0.519		
			MR-PRESSO			0.463		
			MR Egger-intercept			0.828		
herpes zoster	ASD	6	IVW	0.947	0.837–1.072	0.388	2.373	0.795
			MR Egger	0.704	0.430–1.154	0.237	0.901	0.924
			Simple mode	0.975	0.783–1.215	0.832		
			Weighted median	0.954	0.822–1.106	0.530		
			Weighted mode	0.968	0.785–1.194	0.774		
			MR-PRESSO			0.266		
			MR Egger-intercept			0.292		
	ADHD	9	IVW	0.984	0.921–1.050	0.625	2.585	0.958
			MR Egger	0.929	0.728–1.186	0.574	2.358	0.937
			Simple mode	0.964	0.843–1.102	0.602		
			Weighted median	0.971	0.890–1.059	0.506		
			Weighted mode	0.961	0.845–1.093	0.559		
			MR-PRESSO			0.415		
			MR Egger-intercept			0.648		
	TS	10	IVW	0.904	0.754–1.083	0.273	7.594	0.576
			MR Egger	1.073	0.551–2.088	0.841	7.318	0.503
			Simple mode	0.863	0.574–1.299	0.499		
			Weighted median	0.899	0.709–1.140	0.380		
			Weighted mode	0.902	0.627–1.297	0.592		
			MR-PRESSO			0.263		
			MR Egger-intercept			0.614		
EBV infection	ASD	5	IVW	0.959	0.898–1.025	0.216	2.929	0.570
			MR Egger	0.975	0.896–1.062	0.605	2.564	0.464
			Simple mode	0.950	0.827–1.091	0.508		
			Weighted median	0.971	0.896–1.053	0.481		
			Weighted mode	0.985	0.914–1.062	0.715		
			MR-PRESSO			0.222		
			MR Egger-intercept			0.588		
	ADHD	9	IVW	1.008	0.965–1.053	0.722	13.651	0.091
			MR Egger	0.994	0.929–1.063	0.858	13.045	0.071
			Simple mode	1.008	0.934–1.088	0.849		
			Weighted median	1.001	0.958–1.045	0.978		
			Weighted mode	1.002	0.959–1.046	0.935		
			MR-PRESSO			0.731		
			MR Egger-intercept			0.586		
	TS	6	IVW	0.983	0.875–1.104	0.766	1.075	0.956
			MR Egger	1.026	0.863–1.220	0.784	0.632	0.959
			Simple mode	0.925	0.759–1.127	0.473		
			Weighted median	0.989	0.860–1.137	0.876		
			Weighted mode	1.000	0.873–1.146	0.999		
			MR-PRESSO			0.550		
			MR Egger-intercept			0.542		
CMV infection	ASD	3	IVW	0.987	0.930–1.047	0.655	0.267	0.875
			MR Egger	1.024	0.842–1.245	0.850	0.111	0.738
			Simple mode	0.980	0.909–1.057	0.652		
			Weighted median	0.981	0.919–1.048	0.575		
			Weighted mode	0.980	0.909–1.056	0.647		
			MR Egger-intercept			0.761		
	ADHD	3	IVW	1.007	0.968–1.049	0.716	0.034	0.983
			MR Egger	1.010	0.939–1.085	0.836	0.029	0.865
			Simple mode	1.005	0.953–1.061	0.861		
			Weighted median	1.006	0.960–1.053	0.808		
			Weighted mode	1.006	0.954–1.059	0.854		
			MR Egger-intercept			0.953		
	TS	3	IVW	1.027	0.914–1.155	0.651	0.842	0.656
			MR Egger	1.061	0.846–1.330	0.699	0.737	0.391
			Simple mode	1.002	0.851–1.180	0.980		
			Weighted median	1.003	0.870–1.157	0.965		
			Weighted mode	1.002	0.868–1.157	0.977		
			MR Egger-intercept			0.801		
CMV IgG	ASD	4	IVW	0.926	0.813–1.054	0.244	1.237	0.744
			MR Egger	0.772	0.342–1.743	0.597	1.041	0.594
			Simple mode	0.896	0.719–1.116	0.400		
			Weighted median	0.911	0.785–1.059	0.225		
			Weighted mode	0.895	0.719–1.114	0.394		
			MR-PRESSO			0.167		
			MR Egger-intercept			0.701		
	ADHD	7	IVW	1.054	0.964–1.152	0.249	11.064	0.086
			MR Egger	1.031	0.740–1.437	0.864	11.023	0.051
			Simple mode	1.051	0.893–1.237	0.573		
			Weighted median	1.050	0.956–1.154	0.305		
			Weighted mode	0.990	0.849–1.155	0.904		
			MR-PRESSO			0.293		
			MR Egger-intercept			0.897		
	TS	6	IVW	1.135	0.853–1.510	0.385	9.513	0.090
			MR Egger	1.135	0.357–3.612	0.840	9.513	0.049
			Simple mode	0.952	0.571–1.588	0.858		
			Weighted median	1.044	0.778–1.402	0.774		
			Weighted mode	0.965	0.595–1.564	0.890		
			MR-PRESSO			0.425		
			MR Egger-intercept			0.999		

*Note*: MR-PRESSO analysis was only conducted when the number of IVs ≥ 3. The *p*-value less than 0.05 of IVW, MR Egger, Simple mode, Weighted median, and Weighted mode is considered suggestively causal significant. In sensitivity analysis, the *p*-value > 0.05 in MR-Egger intercept and MR-PRESSO indicates the absence of pleiotropy. Heterogeneity was considered non-existent when *p* > 0.05 in Cochran's Q test

Cochrane's Q test and MR-PRESSO method indicated no heterogeneity (*p* > 0.05). Potential SNP directional pleiotropy was also not detected by MR-Egger regression analysis (intercept *p* > 0.05) (Table 2). Least-one-out sensitivity analysis results suggested that the associations were not apparently driven by any single SNP. The visualization of MR analyses including scatter plots, leave-one-out plots, and funnel plots are presented in Additional file 2: Figure S1-S5.

### Systematic review with meta-analysis

A total of 1122 studies were identified from five databases, and 221 duplicate studies were removed. After screening the titles and abstract, 850 studies were excluded. Subsequently, after reviewing the full texts of the remaining studies, 27 eligible studies were included in the review (Fig. 2).

The included studies comprised 25 case–control studies (57 datasets) and 2 retrospective cohort studies. The case–control studies involved 2,024 cases with NDD and 10,759 controls, while the cohort studies included 202 cases of HHV infection and 566 controls without HHV infection. Of all included studies, 13 were from Europe, 10 from Asia, 3 from America, and 1 from Africa. HHV infection detection were assessed by antibodies (13 studies) using enzyme-linked immunosorbent assay (ELISA)/chemiluminescence immunoassay (CILA)/Enzyme immunoassays (EIA) and DNA (14 studies) using polymerase chain reaction (PCR). The samples analyzed included serum, whole blood, white blood cells, dried blood spot (DBS), urine and brain slices. DBS samples were collected within the first postnatal week of the enrolled population, and brain slices were obtained from postmortem brain tissues. More detailed information was provided in Table 3.

**Table 3:** Characteristics of the included studies

Author (year)	Country	Design	Sample source	Detection indicators (methods)	Sample collecting Age (year or days)	Population and control	Exposure/Outcome	Statistical analysis method
**ASD**								
***HSV infection***								
Marylú Mora, 2009 [43]	Venezuela	Case–control study	Serum	HSV IgM (ELISA)	ASD: 11.9 ± 5.56 Control: 4 ± 1.7	ASD = 40 Control = 40	HSV-2 cases = 26/40 (65%) Control = 7/40 (17.5%)	Fisher’s exact test
Carla Lintas, 2010 [44]	Italy	Case–control study	Brain	DNA (PCR)	ASD: 13.36 ± 8.78 Control: 15.23 ± 8.89	ASD = 15 Control = 13	HSV-1: Cases: 1/15 Control: 0/13 HSV-2: Cases: 0/15 Control: 2/13	Chi-squared or Fisher’s exact test
Jianhua Li, 2010 [45]	China	Case–control study	Serum	HSV IgM and IgG (unknown)	ASD: 3.2 ± 1.4 Control: 3.0 ± 1.7	ASD = 208 Control = 192	HSV-IgM: Cases = 2/208 (0.96%) Control = 2/192 (1.04%) HSV-IgG: Cases = 8/208 (3.85%) Control = 11/192 (5.73%)	Chi-squared test
Ivan Gentile, 2014 [46]	Italy	Case–control study	Serum	HSV-1/2 IgG (CLIA)	ASD: 6.1 ± 2.5 Control: 5.9 ± 2.8	ASD = 54 Control = 46	Total IgG: Cases = 16/54 (29.6%) Control = 10/46 (21.7%) HSV-2 IgG: Cases = 1/54 (1.9%) Control = 0/46 (0%)	chi-squared test
Emanuela Zappulo, 2018 [47]	Italy	Case–control study	DBS	HSV-1/2 DNA (PCR)	2–5 days	ASD = 38 Control = 44	HSV-cases = 0/38 Control = 0/44	Chi-squared or Fisher’s exact test
Thayne L. Sweeten, 2019 [48]	Group 1: Utah and Portland	Case–control study	Whole blood	HSV-1/2 DNA (PCR)	ASD:10.7 Control: 12.6	ASD = 80 Control = 60	HSV-cases: 0/80 Control: 0/60	Fisher’s exact test
Thayne L. Sweeten, 2019 [48]	Group 2: California	Case–control study	DBS	HSV-1/2 DNA (PCR)	1–7 days	ASD = 145 Control = 175	HSV-cases: 0/145 Control: 0/175	Fisher’s exact test
Dmitry Maltsev, 2024 [49]	Ukraine	Case–control study	WBC	HSV DNA (PCR)	2–9 years	ASD = 240 Control = 53	HSV-cases = 11/240 (5%) Control = 0/53 (0%)	Student’s t-test and Z-sign test
***VZV infection***								
Ivan Gentile, 2014 [50]	Italy	Case–control study	Serum	VZV IgM (CILA)	ASD: 6.1 ± 2.5 control: 5.9 ± 2.8	ASD = 54 Control = 46	VZV-cases = 32/54 (59.3%) Control = 18/46 (39.1%)	Chi-squared or Fisher’s exact test
Ivan Gentile, 2017 [51]	Italy	Case–control study	DBS	VZV DNA (PCR)	2–5 days	ASD = 38 Control = 44	VZV-cases = 0/38 Control = 0/44	Chi-squared test
Thayne L. Sweeten, 2019 [48]	Group 1: Utah and Portland	Case–control study	Whole blood	VZV DNA (PCR)	ASD: 10.7 Control: 12.6	ASD = 80 Control = 60	VZV-cases: 0/80 Control: 0/60	Fisher’s exact test
Thayne L. Sweeten, 2019 [48]	Group 2: California	Case–control study	DBS	VZV DNA (PCR)	1–7 days	ASD = 145 Control = 175	VZV-cases: 0/145 Control: 0/175	Fisher’s exact test
***EBV infection***								
Marylú Mora, 2009 [43]	Venezuela	Case–control study	Serum	EBV IgG (ELISA)	ASD: 11.9 ± 5.56 Control: 4 ± 1.7	ASD = 40 Control = 40	EBV-cases = 30/40 (75%) Control = 27/40 (67.5%)	Fisher’s exact test
Carla Lintas, 2010 [44]	Italy	Case–control study	Brain	EBV DNA (PCR)	ASD: 13.36 ± 8.78 Control: 15.23 ± 8.89	ASD = 15 Control = 13	EBV-cases: 0/15 Control: 0/13	Chi-squared or Fisher’s exact test
Ivan Gentile, 2014 [52]	Italy	Case–control study	Serum	EBV VCA-IgG (CILA)	ASD: 6.1 ± 2.5 control: 5.9 ± 2.8	ASD = 54 Control = 46	EBV-cases: 29/54 (53.7%) Control: 25/46 (54.3%)	chi-squared or Fisher’s exact test
Thayne L. Sweeten, 2019 [48]	Group 1: Utah and Portland	Case–control study	Whole blood	EBV DNA (PCR)	ASD: 10.7 Control: 12.6	ASD = 80 Control = 60	EBV-cases: 0/80 Control: 0/60	Fisher’s exact test
Thayne L. Sweeten, 2019 [48]	Group 2: California	Case–control study	DBS	EBV DNA (PCR)	1–7 days	ASD = 145 Control = 175	EBV-cases: 1/145 Control: 0/175	Fisher’s exact test
Dmitry Maltsev, 2024 [49]	Ukraine	Case–control study	WBC	EBV DNA (PCR)	2–9 years	ASD = 240 Control = 53	EBV-cases = 153/240 (64%) Control = 12/53 (22%)	Student’s t-test and Z-sign test
***CMV infection***								
Maha I S Kawashti, 2006 [53]	Kingdom of Saudi Arabia	Case–control study	Serum	CMV IgG (EIA)	3–6 years	ASD = 30 Control = 30	CMV-cases = 13/30 (43.3%) Control = 3/30 (10%)	Chi-squared test
Marylú Mora, 2009 [43]	Venezuela	Case–control study	Serum	CMV IgG (ELISA)	ASD: 11.9 ± 5.56 Control: 4 ± 1.7	ASD = 40 Control = 40	CMV-Cases = 20/40 (50%) Control = 22/40 (55%)	Fisher’s exact test
Jianhua Li, 2010 [45]	China	Case–control study	Serum	CMV IgM and IgG (unknown)	ASD: 3.2 ± 1.4 Control: 3.0 ± 1.7	ASD = 208 Control = 192	CMV-IgM: Cases = 5/208 (2.40%) Control = 3/192 (1.56%) CMV-IgG: Cases = 18/208 (8.65%) Control = 12/192 (6.25%)	Chi-squared test
Carla Lintas, 2010 [44]	Italy	Case–control study	Brain	CMV DNA (PCR)	ASD: 13.36 ± 8.78 Control: 15.23 ± 8.89	ASD = 15 Control = 13	CMV-cases: 3/15 Control: 3/13	Chi-squared or Fisher’s exact test
Ivan Gentile, 2014 [52]	Italy	Case–control study	Serum	CMV IgG (CILA)	ASD: 6.1 ± 2.5 Control: 5.9 ± 2.8	ASD = 54 Control = 46	CMV-cases: 18/54 (33.3%) Control: 21/46 (45.7%)	Chi-squared or Fisher’s exact test
Ayako Sakamoto, 2015 [54]	Japan	Observational study with historical control	DBS or dried umbilical cords	CMV DNA (PCR)	6y9m (3y9m–12y7m)	ASD = 27 Control = 3230	CMV-cases = 2/27 (7.4%) Control = 10/3230 (0.31%)	Fisher’s exact test
Mona-Lisa Engman, 2015 [55]	Sweden	Observational study with historical control	DBS	CMV DNA (PCR)	2–5 days	ASD = 115 Control = 6060	CMV-cases = 1/115 (0.9%) Control = 12/6060 (0.2%)	Binomial test
Ivan Gentile, 2017 [56]	Italy	Case–control study	DBS	CMV DNA (PCR)	2–5 days	ASD = 38 Control = 44	CMV-cases = 2/38 (5.3%) Control = 0/44 (0%)	Chi-squared or Fisher’s exact test
Marjolein J Korndewal, 2017 [57]	Netherlands	Retrospective cohort study	DBS	cCMV DNA (PCR)	5y6m ± 2 m	CMV-Exposed: 133 Unexposed: 274	ASD-exposed: 4/133 ASD-unexposed: 5/274	/
Thayne L. Sweeten, 2019 [48]	Group 1: Utah and Portland	Case–control study	Whole blood	CMV DNA (PCR)	ASD: 10.7 Control: 12.6	ASD = 80 Control = 60	CMV-cases: 0/80 Control: 0/60	Fisher’s exact test
Thayne L. Sweeten, 2019 [48]	Group 2: California	Case–control study	DBS	CMV DNA (PCR)	1–7 days	ASD = 145 Control = 175	CMV-cases: 0/145 Control: 0/175	Fisher’s exact test
Chien-Heng Lin, 2021 [58]	China	Retrospective cohort analysis	Urine or blood	CMV DNA (PCR)	CMV-exposed: 3.78 ± 4.66 Unexposed: 3.62 ± 4.50	CMV-exposed: 69 unexposed: 292	ASD-exposed: 4/69 (5.8%) ASD-unexposed: 1/292 (0.34%)	Chi-squared test and Cox proportional hazards regression
Zeinab R Hassan, 2023 [59]	Egypt	Case–control study	Serum and whole blood	CMV IgG and IgM (ELISA) and CMV DNA (PCR)	3–16 years	ASD = 45 Control = 45	ELISA: CMV-cases: 44/45 (97.8%) control: 43/45 (95.6%) PCR: CMV-cases: 25/45 (55.6%) control: 21/45 (46.7%) Total: CMV-cases: 45/45 control: 43/45	Chi-squared test
Dmitry Maltsev, 2024 [49]	Ukraine	Case–control study	WBC	CMV DNA (PCR)	2–9 years	ASD = 240 Control = 53	CMV-cases = 26/240 (11%) Control = 0/53 (0%)	Student’s t-test and Z-sign test
***HHV-6 infection***								
Garth L Nicolson, 2007 [60]	USA	Case–control study	Whole blood	HHV-6 DNA (PCR)	ASD: 8.4 ± 2.8 Control: 7.9 ± 3.3	ASD = 48 Control = 45	HHV6 cases: 14/48 (29.2%) Control: 4/45 (8.3%)	Chi-squared test
Carla Lintas, 2010 [44]	Italy	Case–control study	Brain	HHV-6 DNA (PCR)	ASD: 13.36 ± 8.78 Control: 15.23 ± 8.89	Cases = 15 Control = 13	HHV6 cases: 3/15 Control:1/13	Chi-squared or Fisher’s exact test
Ivan Gentile, 2013 [61]	Italy	Case–control study	Serum	HHV-6 IgG (ELISA)	ASD: 5.83 Control: 5.88	ASD = 30 Control = 28	HHV6 cases: 25/30 (83.3%) Control: 23/28 (81.2%)	Chi-squared or Fisher’s exact test
Thayne L. Sweeten, 2019 [48]	Group 1: Utah and Portland	Case–control study	Whole blood	HHV-6B DNA (PCR)	ASD: 10.7 Control: 12.6	ASD = 80 Control = 60	HHV6 cases: 1/80 Control: 1/60	Fisher’s exact test
Thayne L. Sweeten, 2019 [48]	Group 2: California	Case–control study	DBS	HHV-6B DNA (PCR)	1–7 days	ASD = 145 Control = 175	HHV6 cases: 2/145 Control: 0/175	Fisher’s exact test
Dmitry Maltsev, 2024 [49]	Ukraine	Case–control study	WBC	HHV-6 DNA (PCR)	2–9 years	ASD = 240 Control = 53	HHV6 cases = 162/240 Control = 14/53	Student’s t-test and Z-sign test
***HHV-7 infection***								
Thayne L. Sweeten, 2019 [48]	Group 1: Utah and Portland	Case–control study	Whole blood	HHV-7 DNA (PCR)	ASD: 10.7 Control: 12.6	ASD = 80 Control = 60	HHV7 cases: 0/80 Control: 1/60	Fisher’s exact test
Thayne L. Sweeten, 2019 [48]	Group 2: California	Case–control study	DBS	HHV-7 DNA (PCR)	1–7 days	ASD = 145 Control = 175	HHV7 cases: 0/145 Control: 0/175	Fisher’s exact test
Dmitry Maltsev, 2024 [49]	Ukraine	Case–control study	WBC	HHV-7 DNA (PCR)	2–9 years	ASD = 240 Control = 53	HHV7 cases = 172/240 Control = 15/53	Student’s t-test and Z-sign test
***HHV-8 infection***								
Ivan Gentile, 2013 [61]	Italy	Case–control study	Serum	HHV-8 IgG	ASD: 5.83 Control: 5.88	ASD = 30 Control = 28	HHV8 cases: 1/30 (3.3%) Control: 0/28	chi-squared or Fisher’s exact test
Thayne L. Sweeten, 2019 [48]	Group 1: Utah and Portland	Case–control study	Whole blood	HHV-8 DNA (PCR)	ASD: 10.7 Control: 12.6	ASD = 80 Control = 60	HHV8 cases: 0/80 Control: 0/60	Fisher’s exact test
Thayne L. Sweeten, 2019 [48]	Group 2: California	Case–control study	DBS	HHV-8 DNA (PCR)	1–7 days	ASD = 145 Control = 175	HHV8 cases: 0/145 Control: 0/175	Fisher’s exact test
**ADHD**								
***HSV infection***								
Mervan Bekdas, 2014 [17]	Turkey	Case–control study	Serum	HSV IgG (ELISA)	ADHD:9.0 ± 2.2 Control:9.8 ± 2.9	ADHD = 60 Control = 30	HSV-cases = 38/60 (63.3%) Control = 24/30 (80%)	Chi-Square test
***VZV infection***								
Mervan Bekdas, 2014 [17]	Turkey	Case–control study	Serum	VZV IgG (ELISA)	ADHD:9.0 ± 2.2 Control:9.8 ± 2.9	ADHD = 60 Control = 30	VZV-cases = 54/60 (90%) Control = 26/30 (86.7%)	Chi-Square test
***EBV infection***								
Mervan Bekdas, 2014 [17]	Turkey	Case–control study	Serum	EBV IgG (ELISA)	ADHD:9.0 ± 2.2 Control:9.8 ± 2.9	ADHD = 60 Control = 30	EBV-cases = 51/60 (85%) Control = 25/30 (83.3%)	Chi-Square test
***CMV infection***								
Ruizhen Chai, 2005 [62]	China	Case–control study	Serum	CMV IgM (ELISA)	ADHD:11.7 Control:10.9	Cases = 201 Control = 265	Cases = 192/201 (95.5%) Control = 21/265 (8.2%)	Chi-Square test
Mervan Bekdas, 2014 [17]	Turkey	Case–control study	Serum	CMV IgG (ELISA)	ADHD:9.0 ± 2.2 Control:9.8 ± 2.9	Cases = 60 Control = 30	Cases = 57/60 (95%) Control = 29/30 (96.6%)	Chi-Square test
Marjolein J Korndewal, 2017 [57]	Netherlands	Retrospective cohort study	DBS	cCMV DNA (PCR)	5y6m ± 2 m	cCMV-Exposed: 133 Unexposed: 274	ADHD-exposed: 1/133 (0.8%) ADHD-unexposed: 7/274 (2.6%)	Chi-Square test
Chien-Heng Lin, 2021 [58]	China	Retrospective cohort analysis	Urine or blood	CMV DNA (PCR)	CMV-exposed: 3.78 ± 4.66 unexposed: 3.62 ± 4.50	CMV-exposed: 69 Unexposed: 292	ADHD-exposed:2/69 (2.89%) ADHD-Unexposed: 8/292 (2.73%)	Chi-Square test
**TD**								
***EBV infection***								
Jiangyu Chen, 2012 [63]	China	Case–control study	Whole blood	EBV DNA (PCR)	TD: 7.89 ± 2.65 Control:6.93 ± 1.69	TD = 49 Control = 47	EBV-Cases = 11/49 (22.49%) Control = 1/47 (2.13%)	Chi-Square test
Yanzhao Chen, 2017 [64]	China	Case–control study	Serum	EBV IgM (ELISA)	TD: 7.45 Control:7.15	TD = 80 Control = 40	EBV-Cases = 8/80 (10%) Control = 1/40 (2.5%)	Chi-Square test
Jialin Xu, 2020 [65]	China	Case–control study	Whole blood	EBV DNA (PCR)	TD: 6.9 ± 1.4 Control: 7.5 ± 1.2	TD = 100 Control = 100	EBV-Cases = 27/100 Control = 4/100	Chi-Square test
***CMV infection***								
Guifang Kuang, 2001 [66]	China	Case–control study	Whole blood	HCMV DNA (PCR)	TD: 7.6 ± 2.3 Control: 6.8 ± 0.6	TD = 66 Control = 74	CMV-Cases = 17/66 (26%) Control = 2/74 (3%)	Chi-Square test
Guifang Kuang, 2005 [67]	China	Case–control study	Whole blood	HCMV DNA (PCR)	TD: 7.89 ± 2.65 Control:6.93 ± 1.69	TD = 101 Control = 34	CMV-Cases = 37/101 (36.6%) Control = 1/34 (2.9%)	Chi-Square test
Ruizhen Chai, 2005 [62]	China	Case–control study	Serum	CMV IgM (ELISA)	TD:10.8 Control:10.9	TDs = 116 Control = 265	CMV-Cases = 112/116 (96.6%) Control = 21/265 (8.2%)	Chi-Square test
Yanhui Chen, 2006 [68]	China	Case–control study	Serum	CMV IgM (ELISA)	TD:8 ± 2 Control:8 ± 2	TD = 60 Control = 20	CMV-Cases = 2/60 Control = 1/20	Chi-Square test
Jiangyu Chen, 2012 [63]	China	Case–control study	Urine	HCMV DNA (PCR)	TD: 7.89 ± 2.65 Control:6.93 ± 1.69	TD = 49 Control = 47	CMV-Cases = 3/49 (6.12%) Control = 0/47 (0%)	Chi-Square test
Yanzhao Chen, 2017 [64]	China	Case–control study	Serum	CMV IgM (ELISA)	TD: 7.45 Control:7.15	TD = 80 Control = 40	CMV-Cases = 5/80 (6.25%) Control = 1/40 (2.5%)	Chi-Square test
Jialin Xu, 2020 [65]	China	Case–control study	Whole blood	CMV DNA (PCR)	TD: 6.9 ± 1.4 Control: 7.5 ± 1.2	TD = 100 Control = 100	CMV-Cases = 19/100 Control = 0/100	Chi-Square test

We performed separate meta-analyses based on subfamilies of HHV. The results were listed in Table 4 and the forest plots, leave-one-out plots and funnel plots were provided in Additional file 3. The pooled estimates found that a higher proportion of ASD experienced CMV infection and HHV-6 infection when comparing with non-ASD (CMV-pOR: 2.66, 95% CI 1.15–6.15, I^2^ = 66%; HHV-6-pOR: 3.93, 95% CI 2.39–6.45, I^2^ = 19%) (Additional file 3: Figure S3/S6). The relative risk of EBV infection and CMV infection were significantly increased in Chinese TD populations (EBV-pOR: 8.48, 95% CI 3.51–20.50, I^2^ = 0%; CMV-pOR: 14.16, 95% CI 2.88–69.61, I^2^ = 83%) (Additional file 3: Figure S8/S9). A potential association between EBV infection and ASD was revealed by pOR 2.19 (95% CI 0.80–5.99; I^2^ = 77%) (Additional file 3: Figure S2). HSV infection and HHV-7 infection was not significantly associated with ASD (HSV-pOR: 1.83, 95%CI 0.57–5.84, I^2^ = 74%; HHV-7-pOR: 1.87, 95%CI 0.08–41.34, I^2^ = 74%) (Additional file 3: Figure S1/S7), CMV infection was also not significantly associated with ADHD (OR: 14.12, 95%CI 0.04–4727.02; I^2^ = 96%) (Additional file 3: Figure S10). However, in the retrospective cohort studies, the cCMV infection group showed no significant increase in the probability of ASD (OR: 4.61, 95% CI 0.46–46.08; I^2^ = 69%) and ADHD (OR: 0.59, 95% CI 0.17–2.05; I^2^ = 0%) comparing with control group (Additional file 3: Figure S11/S12).

**Table 4:** Meta-analysis and subgroup analysis of association between NDDs and HHVs

Group	n	OR (95%CI)	Heterogeneity test		Subgroup difference	
			I^2^ (%)	*p*-value	*p*-value	
***Case–control studies***						
ASD and HSV						
**Total**	8	1.83 (0.57–5.84)	74%	< 0.01		
**Detection indicator**					0.78	
-Antibody	3	2.03 (0.46–8.89)	85%	< 0.01		
-DNA	5	1.33 (0.10–17.16)	45%	0.18		
ASD and EBV						
**Total**	6	2.19 (0.80–5.99)	77%	< 0.01		
**Detection indicator**					< 0.01	
-Antibody	2	1.14 (0.62–2.10)	0%	0.54		
-DNA	4	5.88 (2.98–11.59)	0%	0.77		
**Region**					0.66	
-America	2	1.56 (0.61–3.97)	0%	0.59		
-Europe	4	2.44 (0.41–14.53)	91%	< 0.01		
ASD and CMV						
**Total**	12	2.66 (1.15–6.15)	66%	< 0.01		
**Sample collection time**					0.01	
-after neonatal period	8	1.53 (0.71–3.32)	56%	0.03		
-a postnatal week	4	9.84 (2.97–32.54)	0%	0.48		
**Detection indicator**					0.12	
-Antibody	5	1.34 (0.60–3.02)	60%	0.04		
-DNA	7	3.96 (1.34–11.74)	56%	0.03		
**Sample source**					0.03	
-Serum	5	1.34 (0.60–3.02)	60%	0.04		
-Dried blood spot	4	9.84 (2.97–32.54)	0%	0.48		
**Region**					0.43	
-America	2	0.91 (0.39–2.12)	0%	0.38		
-Asia	3	5.64 (1.05–30.24)	84%	< 0.01		
-Europe	6	1.82 (0.54–6.16)	51%	0.08		
ASD and HHV-6						
**Total**	6	3.93 (2.39–6.45)	19%	0.29		
**Region**					0.48	
-America	2	4.44 (1.45–13.56)	0%	0.82		
-Europe	4	2.53 (0.84–7.57)	51%	0.11		
ASD and HHV-7						
**Total**	2	1.87 (0.08–41.34)	74%	0.05		
ADHD and CMV						
**Total**	2	14.12 (0.04–4727.02)	96%	< 0.01		
TD and EBV						
**Total**	3	8.48 (2.51–20.50)	0%	0.75		
TD and CMV						
**Total**	7	14.16 (2.88–69.61)	83%	< 0.01		
**Detection indicator**					0.80	
-Antibody	3	9.53 (0.21–411.27)	93%	< 0.01		
-DNA	4	15.69 (5.53–44.54)	0%	0.80		
***Retrospective cohort studies***						
CMV and ASD						
**Total**	2	4.61 (0.46–46.08)	69%	0.07		
CMV and ADHD						
**Total**	2	0.59 (0.17–2.05)	0%	0.33		

The subgroup analyses stratified by continent, detection indicators, sample sources and collection time were performed, as shown in Table 3 and Additional file 3. CMV infection detected using DBS in the postnatal first week had a significant association with ASD (OR: 9.84, 95% CI 2.97–32.54, I^2^ = 0%) (Additional file 3: Figure S4/S5). Regarding detection indicators, a higher proportion of ASD patients was found EBV-DNA positive (OR: 5.88, 95% CI 2.98–11.59, I^2^ = 0%) and CMV-DNA positive (OR: 3.96, 95% CI 1.34–11.74, I^2^ = 56%) (Additional file 3: Figure S2/S4). An increased risk of TD was also observed in CMV-DNA positive patients (OR: 15.69, 95% CI 5.53–44.54, I^2^ = 0%) (Additional file 3: Figure S8). The risk of ASD in EBV or CMV positive patients did not significantly increase in either America or Europe, whereas an increased proportion of ASD patients experienced CMV infection in Asia (pOR: 5.64, 95% CI 1.05–30.24, I^2^ = 84%) (Additional file 3: Figure S5) and HHV-6 infection in America (pOR: 4.44, 95% CI 1.45–13.56, I^2^ = 0%) (Additional file 3: Figure S6).

## Discussion

This study employed a two-sample MR analysis to investigate the association between HHVs and ASD, ADHD, and TS. We included HSV, VZV, EBV, and CMV-related SNPs as IVs after removing weak SNPs in MR analysis and no association was found between these HHVs infection and risk of ASD, ADHD, and TS. According to meta-analysis, ASD patients had a greater proportion of CMV and HHV-6 infection than non-ASDs. The relative risk of EBV and CMV infection was much higher in Chinese TD populations.

Previous studies have proposed an association between viral infections and ASD, yet the precise mechanisms remain unclear and may involve several possibilities: direct infection of the CNS, infection in peripheral sites triggering CNS-related conditions, or modulation of immune responses affecting ASD [15]. Viruses can damage neurons through cell lysis or inducing apoptosis. Activation of the immune system after viral infections can also affect neurons through inflammatory responses, release of free radicals, imbalance in cytokines, and production of autoantibodies [69]. Neurobehavioral disorders, including ASD, are associated with structural and functional deficits in CNS neurons [70].

Our results did not find that HSV or VZV infections increase the risk of ASD. HSV infection in early embryonic development HSV has been proposed to activate the immune system and induce inflammatory responses, leading to abnormal growth in the cerebral cortex, which commonly observed in children with ASD [71]. However, due to the low incidence of congenital HSV infection and the limited sample size of the study, the detection rate of HSV DNA was 0 in both the ASD and control groups [47, 48]. For HSV infection, regardless of the time of infection, there was significant heterogeneity among different studies. This variability could be influenced by factors such as ethnicity, region, detection methods, and social factors. VZV is also a neurotropic virus associated with CNS inflammation and CNS diseases like multiple sclerosis, shares some pathological features with ASD [72]; however, evidence supporting its direct involvement in ASD pathogenesis remains scarce. Only a small-scale case–control study has indicated a higher VZV antibodies seropositivity among children with ASD compared to controls [50]. Future research with larger sample sizes is needed to further explore the potential correlation.

CMV has the potential to invade the CNS at any stage of neural development, provoke inflammation by inhibiting host antiviral defenses such as interferon, and elicit local infiltration of macrophages and T cells at infection sites, thereby altering immune function and causing developmental anomalies in specific brain regions or structures, potentially contributing to ASD [73]. The subgroup analysis revealed that CMV infection detected from DBS collected within one week after birth was significantly associated ASD. The detection of CMV infection in older children showed no significant correlation with ASD. This is because most studies detect CMV antibodies, which may include postnatal or asymptomatic infections that do not affect neurodevelopment. It indicates that congenital or perinatal CMV infection is a high-risk factor for ASD. This finding is consistent with the results of a systematic review published in 2017 on cCMV infection and ASD. In addition to the direct damage to the brain caused by cCMV, the immune response following maternal CMV infection during pregnancy can also affect the fetal nervous system [74]. However, MR analysis found no evidence between ASD and CMV. A recent MR study based on another European population GWAS also failed to establish a significant association between CMV infection and ASD [75]. It appears that CMV and ASD share no common genetic susceptibility based on current data. CMV infection may correlate with ASD through complicated immune responses.

There is limited research investigating the roles of EBV in the pathogenesis of ASD. Combining the results from the current observational studies, it was found that EBV infection may be associated with ASD. Sensitivity analysis revealed that removing the study by Ivan et al. [52] resulted in significant correlation, suggesting that this study is one source of heterogeneity. This may be due to the use of CLIA technology for detecting EBV antibodies, which generally has higher sensitivity compared to methods like ELISA. Additionally, subgroup analysis showed that congenital or perinatal EBV infection is significantly associated with ASD. This suggests that, similar to CMV, EBV may be involved in ASD-related neural damage or immune responses during early embryonic development. Future research is warranted to explore the mechanisms by which congenital EBV infection affects children's neurodevelopment.

HHV-6 and HHV-7 are common pathogens causing exanthema subitum in infants and can also lead to febrile seizures and encephalitis [76]. HHV-8 is the causative agent of Kaposi's sarcoma [77]. Our results suggest that more patients with ASD had HHV-6 infection when comparing with non-ASD control group. Although there are currently no studies on the mechanism by which HHV-6 associating with ASD, it is known that HHV-6 can enter the CNS through the nasopharynx and olfactory pathways, infecting various neural cells [78]. Additionally, HHV-6 is usually acquired between 6 and 15 months of age, suggesting its potential role in children's neurodevelopment [79]. A comprehensive understanding of HHV-6 and its relationship with ASD in the future will help elucidate why HHV-6 increases the risk of ASD. Moreover, research on the association between HHV-7/8 infections and ASD is limited and seems to show no significant correlation. Further studies with large sample sizes or multicenter approaches are needed.

In comparison to ASD, research concerning ADHD is relatively sparse. Currently, the prevailing pathogenesis of ADHD includes autoimmune and viral infection hypotheses. It was speculated that lentiviral particles may lay dormant within the CNS, with viral proteins potentially translated upon exposure to exogenous antigens and other stimuli, causing immune response dysregulation [80]. Human CMV may influence the pathogenesis of ADHD by regulating glutamate uptake and transporter expression in astrocytes [81]. However, a retrospective study conducted in an Asian population failed to observe a significant increase in subsequent ADHD incidence among individuals with CMV infection [58]. Similarly, in the Turkish population, no statistical differences were noted in serum IgG levels of HSV-1, VZV, EBV and CMV between ADHD patients and healthy children [17]. In addition, research into HSV-1 susceptibility genes did not reveal an accumulation in ADHD cases [82], indirectly suggesting minimal influence of HSV on ADHD incidence. In a Greek birth cohort study, HSV-1/2, EBV, and CMV infection were not associated with elevated ADHD test scores [83]. Nonetheless, a notable disparity persisted between simply increased ADHD test scores and clinical ADHD diagnoses, underscoring the need for further follow-up investigations to bolster these findings.

In terms of TD, its etiology remains elusive, with a potential involvement of genetic, immune, psychological, and environmental factors [84]. While viral infections are mostly associated with immune responses, the ‘kindling hypothesis’ has been proposed by some researchers, suggesting that infections may trigger sustained hypersensitivity reactions, leading to tic symptoms due to immune imbalance [85]. Antibodies or T cells produced after viral infections may also cross-react with autoantigens, exacerbating tic symptoms through an autoimmune response [86]. Furthermore, acute and chronic infection could increase the level of various pro-inflammatory cells [87], directly or indirectly activate the metabolism and decomposition of tryptophan in the CNS via pro-inflammatory cytokines, thereby affecting the neurotransmitter balance in the brain and contributing to tic symptoms [88]. Additionally, post-infection defects in T-regulatory cells leading to diminished autologous reactive lymphocyte capacity, potentially elucidating diverse tic symptoms observed in certain TS patients [89].

Despite these insights, limited attention has been given to exploring the association between HHVs and TD around the world. A case report mentioned that a girl without any history of neuropsychiatric illness appeared multiple motor and vocal tics one month following herpetic encephalitis [90]. Another case report documented an association between recurrent HSV-1 infection and exacerbation of tic symptoms in children with TS [91]. Several studies in China have found that EBV and CMV infection significantly correlated with TD (Table 3), potentially due to immune system dysregulation triggered by these infections. Given the constraints of single regional reports and small sample sizes, general conclusion cannot be drawn from the meta-analysis of all Chinese studies. In light of our MR analysis results, we have not found a direct association between HHVs infection and the risk of TS based on European population, the potential role of viral infection in initiating and/or exacerbating TS symptoms cannot be dismissed, which requires further research.

In spite of the high prevalence of HHV infection in the population, unlike influenza, most infections are asymptomatic or latent. Nonetheless, silent viral infections can still induce immune responses, potentially leading to NDDs [15]. Asymptomatic infections possess the capability to reactivate at any point in life [92], and studies based on subjective reported infections may result in either overestimation or underestimation of infection cases. Furthermore, children included in studies may not exhibit strong immune responses to HHVs present in their bodies, leading to false negatives due to low antibody levels or false positives owing to variations in detection sensitivity. Even with viral DNA detection, challenges persist regarding low detection rates in blood samples [48]. Additionally, some previous studies were observational, prone to bias in participant selection and outcome assessment blinding. The limited number of studies included in the meta-analysis makes it challenging to identify the sources of publication bias. Visually, the funnel plot for CMV and ASD appears asymmetrical; however, statistical tests of the funnel plot suggest the absence of publication bias (Rank correlation on funnel plot asymmetry: z-score = 0.70, *p* = 0.484 > 0.05). It is important to note that these statistical tests often have low power, meaning that even if the statistical results do not provide evidence of asymmetry, the possibility of bias cannot be entirely ruled out [93]. Besides, most observational studies involved cases with multiple viral co-infections, complicating the interpretation of causality and potentially leading to false-positive results.

Our MR analysis also has some limitations. Firstly, the data used in this study are from European populations, thus limiting the generalizability of conclusions globally due to disparities in HHV infection rates across regions. Future MR analyses in diverse populations are still needed. Secondly, although IVs were derived from recent large-scale GWAS studies, the final analysis included fewer strongly correlated variables, leading to the adoption of lenient thresholds. Moreover, a greater number of weak IVs related to viral infections, particularly antibody levels, were not included in the MR analysis under stringent criteria (F > 10). More robust IVs are needed to supplement the research in the future. Thirdly, genetic variants of outcomes were extracted from publicly available summary data from the PGC, where missing EAF values in NDDs preclude the supplementation of EAF values from normal populations. Consequently, reverse MR analysis or MR Steiger analysis cannot be conducted, leaving room for exploration of the directionality between exposure and outcome.

## Conclusions

Our results offer evidence that HSV, VZV, EBV, and CMV infections do not directly increase the risk of ASD, ADHD, and TS through two-sample MR analyses. HHV-6 infection and cCMV infection were associated with an increased risk of ASD based on the results of meta-analysis. These findings, along with previous published studies, highlight the complexity of viral infections in childhood NDDs. To further investigate the contribution of HHV infections to the risk of ASD, ADHD, and TD, larger population studies, comprehensive data of HHV infections, and more robust analyses are warranted.

## Supplementary Information

Supplementary file 1.

Supplementary file 2.

Supplementary file 3.

Supplementary file 4.

## Data Availability

The majority of data presented in the study are included in the article and supplemental files, the sources of original datasets are presented in the Methods and R codes used and/or analyzed are available from the corresponding author on reasonable request.

## Abbreviations

NDDs: Neurodevelopmental disorders
CNS: Central nervous system
ASD: Autism spectrum disorder
ADHD: Attention deficit hyperactivity disorder
TS: Tourette syndrome
HHVs: Human herpesviruses
HSV: Herpes simplex virus
VZV: Varicella-zoster virus
CMV: Cytomegalovirus
EBV: Epstein-Barr virus
MR: Mendelian randomization
IVs: Instrumental variables
GWAS: Genome-wide association studies
SNPs: Single nucleotide polymorphisms
IVW: Inverse-variance weighted
PRISMA: Preferred Reporting Items for Systematic reviews and Meta-Analyses
DSM: Diagnostic and statistical manual of mental disorders
ICD: International Classification of Diseases
NOS: Newcastle-Ottawa Rating Scale
ELISA: Enzyme-linked immunosorbent assay
CILA: Chemiluminescence immunoassay
EIA: Enzyme immunoassays
PCR: Polymerase chain reaction
DBS: Dried blood spot
