# Supplementary material for: Association between human herpesviruses infections and childhood neurodevelopmental disorders: insights from two-sample mendelian randomization analyses and systematic review with meta-analysis
Source: Ital J Pediatr. 2024 Nov 20;50:248. doi: 10.1186/s13052-024-01820-9 (PMC11580506; doi:10.1186/s13052-024-01820-9)
Supplement: Supplementary file 1 [file 13052_2024_1820_MOESM1_ESM.pdf]

STROBE-MR checklist[1 2]

Item No.	Section		Checklist item	Page No.	Relevant text from manuscript
1	**TITLE and ABSTRACT**		Indicate Mendelian randomization (MR) as the study's design in the title and/or the abstract if that is a main purpose of the study	1	Line1-3
	**INTRODUCTION**				
2	**Background**		Explain the scientific background and rationale for the reported study. What is the exposure? Is a potential causal relationship between exposure and outcome plausible? Justify why MR is a helpful method to address the study question	3-5	Line 50-97
3	**Objectives**		State specific objectives clearly, including pre-specified causal hypotheses (if any). State that MR is a method that, under specific assumptions, intends to estimate causal effects	5	Line 98-101
	**METHODS**				
4	**Study design and data sources**		Present key elements of the study design early in the article. Consider including a table listing sources of data for all phases of the study. For each data source contributing to the analysis, describe the following:		
		a)	Setting: Describe the study design and the underlying population, if possible. Describe the setting, locations, and relevant dates, including periods of recruitment, exposure, follow-up, and data collection, when available.	6-7	Line 117-134
		b)	Participants: Give the eligibility criteria, and the sources and methods of selection of participants. Report the sample size, and whether any power or sample size calculations were carried out prior to the main analysis	6-7	Line 117-134
		c)	Describe measurement, quality control and selection of genetic variants	7	Line 136-148
		d)	For each exposure, outcome, and other relevant variables, describe methods of assessment and diagnostic criteria for diseases	6-7	Line 117-134
		e)	Provide details of ethics committee approval and participant informed consent, if relevant		N/A
5	**Assumptions**		Explicitly state the three core IV assumptions for the main analysis (relevance, independence and exclusion restriction) as well assumptions for any additional or sensitivity analysis	6	Line 108-115
6	**Statistical methods: main analysis**		Describe statistical methods and statistics used		

		a)	Describe how quantitative variables were handled in the analyses (i.e., scale, units, model)		The statistical effect size or measurement units related to exposure have not been converted or reported.
		b)	Describe how genetic variants were handled in the analyses and, if applicable, how their weights were selected	7	Line 136-148
		c)	Describe the MR estimator (e.g. two-stage least squares, Wald ratio) and related statistics. Detail the included covariates and, in case of two-sample MR, whether the same covariate set was used for adjustment in the two samples	7-8	Line 150-153; Covariates were not included in our study.
		d)	Explain how missing data were addressed		N/A
		e)	If applicable, indicate how multiple testing was addressed		N/A
7	**Assessment of assumptions**		Describe any methods or prior knowledge used to assess the assumptions or justify their validity	7	Line 136-144
8	**Sensitivity analyses and additional analyses**		Describe any sensitivity analyses or additional analyses performed (e.g. comparison of effect estimates from different approaches, independent replication, bias analytic techniques, validation of instruments, simulations)	8	Line 153-161
9	**Software and pre-registration**				
		a)	Name statistical software and package(s), including version and settings used	8	Line 166-167
		b)	State whether the study protocol and details were pre-registered (as well as when and where)	8	Line 172-173
	**RESULTS**				
10	**Descriptive data**				
		a)	Report the numbers of individuals at each stage of included studies and reasons for exclusion. Consider use of a flow diagram	11	Line 229-235
		b)	Report summary statistics for phenotypic exposure(s), outcome(s), and other relevant variables (e.g. means, SDs, proportions)		Table S1
		c)	If the data sources include meta-analyses of previous studies, provide the assessments of heterogeneity across these studies		N/A
		d)	For two-sample MR:<br>i. Provide justification of the similarity of the genetic variant-exposure associations between the exposure and outcome samples	6-7	Line 117-134; Both exposure and outcomes are derived from European populations, resulting in low ethnic heterogeneity. There are no populations that overlap between the databases from which the exposure and outcome data are derived.

			ii. Provide information on the number of individuals who overlap between the exposure and outcome studies		
11	**Main results**				
		a)	Report the associations between genetic variant and exposure, and between genetic variant and outcome, preferably on an interpretable scale		Table 2; Table S1 and S2
		b)	Report MR estimates of the relationship between exposure and outcome, and the measures of uncertainty from the MR analysis, on an interpretable scale, such as odds ratio or relative risk per SD difference		Table 2
		c)	If relevant, consider translating estimates of relative risk into absolute risk for a meaningful time period		N/A
		d)	Consider plots to visualize results (e.g. forest plot, scatterplot of associations between genetic variants and outcome versus between genetic variants and exposure)		Figure 3; additional file 2
12	**Assessment of assumptions**				
		a)	Report the assessment of the validity of the assumptions		Table 1 and 2
		b)	Report any additional statistics (e.g., assessments of heterogeneity across genetic variants, such as I^2, Q statistic or E-value)		Table 1 and 2, Table S1
13	**Sensitivity analyses and additional analyses**				
		a)	Report any sensitivity analyses to assess the robustness of the main results to violations of the assumptions	12	Line 243-253; Table 2
		b)	Report results from other sensitivity analyses or additional analyses	12	Line 254-256; Table 2
		c)	Report any assessment of direction of causal relationship (e.g., bidirectional MR)		N/A
		d)	When relevant, report and compare with estimates from non-MR analyses		N/A
		e)	Consider additional plots to visualize results (e.g., leave-one-out analyses)		Additional file 2
	**DISCUSSION**				
14	**Key results**		Summarize key results with reference to study objectives	15	Line 307-310
15	**Limitations**		Discuss limitations of the study, taking into account the validity of the IV assumptions, other sources of potential bias, and imprecision. Discuss both direction and magnitude of any potential bias and any efforts to address them	20-21	Line 434-445

16	**Interpretation**				
		a)	Meaning: Give a cautious overall interpretation of results in the context of their limitations and in comparison with other studies	15-19	Line 313-415
		b)	Mechanism: Discuss underlying biological mechanisms that could drive a potential causal relationship between the investigated exposure and the outcome, and whether the gene-environment equivalence assumption is reasonable. Use causal language carefully, clarifying that IV estimates may provide causal effects only under certain assumptions	15-19	Line 313-415
		c)	Clinical relevance: Discuss whether the results have clinical or public policy relevance, and to what extent they inform effect sizes of possible interventions	15-20	Line 313-433
17	**Generalizability**		Discuss the generalizability of the study results (a) to other populations, (b) across other exposure periods/timings, and (c) across other levels of exposure	19	Line 412-415
	**OTHER INFORMATION**				
18	**Funding**		Describe sources of funding and the role of funders in the present study and, if applicable, sources of funding for the databases and original study or studies on which the present study is based		N/A
19	**Data and data sharing**		Provide the data used to perform all analyses or report where and how the data can be accessed, and reference these sources in the article. Provide the statistical code needed to reproduce the results in the article, or report whether the code is publicly accessible and if so, where	23	Line 482-484
20	**Conflicts of Interest**		All authors should declare all potential conflicts of interest	22	Line 472

This checklist is copyrighted by the Equator Network under the Creative Commons Attribution 3.0 Unported (CC BY 3.0) license.

1. Skrivankova VW, Richmond RC, Woolf BAR, Yarmolinsky J, Davies NM, Swanson SA, et al. Strengthening the Reporting of Observational Studies in Epidemiology using Mendelian Randomization (STROBE-MR) Statement. JAMA. 2021;under review.

2. Skrivankova VW, Richmond RC, Woolf BAR, Davies NM, Swanson SA, VanderWeele TJ, et al. Strengthening the Reporting of Observational Studies in Epidemiology using Mendelian Randomisation (STROBE-MR): Explanation and Elaboration. BMJ. 2021;375:n2233.

Table S1. Details of all the genetic variants of different kinds of human herpesviruses infections.

SNP	r_allele.expo	_allele.exp	pval.exposure	eta.exposu	e.exposure	af.exposur	exposure	keep.expo	origin.expo	ncase	ncontrol	samplesize	R2	F
rs77231357	A	G	1.92E-06	0.227101	0.047696	0.071639	HSV infections	TRUE	reported	3723	396378	400101	0.00686	2763.701
rs59142651	G	A	4.16E-06	-0.45146	0.098077	0.012613	HSV infections	TRUE	reported	3723	396378	400101	0.005077	2041.518
rs9263969	C	T	6.88E-07	-0.13625	0.027444	0.225158	HSV infections	TRUE	reported	3723	396378	400101	0.006478	2608.592
rs75710096	A	G	3.73E-06	0.234185	0.050627	0.060466	HSV infections	TRUE	reported	3723	396378	400101	0.006231	2508.719
rs10234639	T	G	4.55E-06	0.120153	0.026208	0.280967	HSV infections	TRUE	reported	3723	396378	400101	0.005833	2347.532
rs4716482	A	C	7.20E-07	-0.11575	0.023356	0.490196	HSV infections	TRUE	reported	3723	396378	400101	0.006696	2697.261
rs10961236	G	A	2.01E-06	0.125742	0.026458	0.270592	HSV infections	TRUE	reported	3723	396378	400101	0.006241	2512.828
rs138557714	T	A	1.80E-06	-0.49601	0.103882	0.010612	HSV infections	TRUE	reported	3723	396378	400101	0.005166	2077.729
rs2004786	G	T	4.62E-06	-0.11469	0.025034	0.305656	HSV infections	TRUE	reported	3723	396378	400101	0.005583	2246.482
rs71428759	T	G	2.39E-06	0.323057	0.068485	0.033132	HSV infections	TRUE	reported	3723	396378	400101	0.006686	2693.266
rs12457005	T	C	2.32E-07	0.289393	0.055957	0.050226	HSV infections	TRUE	reported	3723	396378	400101	0.00799	3222.612
rs148444866	G	A	3.79E-06	0.31358	0.06784	0.033801	HSV infections	TRUE	reported	3723	396378	400101	0.006423	2586.321
rs75088484	A	G	2.87E-06	-0.53702	0.114745	0.025164	is and keratoco	TRUE	reported	1252	390647	391899	0.014149	5624.552
rs2858331	A	G	3.28E-06	-0.20188	0.043389	0.280351	is and keratoco	TRUE	reported	1252	390647	391899	0.016444	6552.257
rs55848335	A	T	6.89E-07	-0.22866	0.046059	0.234464	is and keratoco	TRUE	reported	1252	390647	391899	0.01877	7496.539
rs78742138	T	C	2.35E-06	0.411086	0.087087	0.062831	is and keratoco	TRUE	reported	1252	390647	391899	0.019902	7957.759
rs4660006	A	G	2.52E-07	-0.19448	0.037718	0.758398	anogenital infe	TRUE	reported	1986	400197	402183	0.01386	5652.645
rs708292	T	C	1.88E-06	0.233202	0.048927	0.887955	anogenital infe	TRUE	reported	1986	400197	402183	0.010821	4399.717
rs62282633	G	C	1.98E-06	0.432679	0.090991	0.036458	anogenital infe	TRUE	reported	1986	400197	402183	0.013153	5360.41
rs58100391	T	A	2.40E-06	-0.18784	0.039824	0.193428	anogenital infe	TRUE	reported	1986	400197	402183	0.01101	4477.113
rs112841441	G	A	5.29E-07	-0.41452	0.08265	0.033302	anogenital infe	TRUE	reported	1986	400197	402183	0.011063	4499.115
rs1738233	A	T	1.45E-06	-0.07238	0.014865	0.573657	HSV-1 IgG	TRUE	reported	645	NA	645	0.002563	1.652151
rs58599785	T	C	4.91E-06	0.086518	0.018756	0.165511	HSV-1 IgG	TRUE	reported	645	NA	645	0.002068	1.332282
rs3132935	G	A	2.76E-06	0.093733	0.019795	0.167244	HSV-1 IgG	TRUE	reported	645	NA	645	0.002447	1.577471
rs10977313	T	G	2.97E-07	-0.12527	0.024149	0.107452	HSV-1 IgG	TRUE	reported	645	NA	645	0.00301	1.941135
rs10888851	G	C	3.27E-06	-0.2401	0.049849	0.109195	HSV-2 IgG	TRUE	reported	208	NA	208	0.011215	2.336417
rs10782620	G	T	2.60E-06	0.162769	0.033434	0.396552	HSV-2 IgG	TRUE	reported	208	NA	208	0.01268	2.64559
rs10174926	C	T	9.72E-07	-0.23643	0.04647	0.126437	HSV-2 IgG	TRUE	reported	208	NA	208	0.012348	2.575488
rs35213774	G	A	1.10E-06	0.265169	0.052409	0.112069	HSV-2 IgG	TRUE	reported	208	NA	208	0.013994	2.923667
rs355547	C	T	2.00E-06	0.17259	0.035026	0.387931	HSV-2 IgG	TRUE	reported	208	NA	208	0.014145	2.955769
rs72804080	G	A	1.92E-07	0.259082	0.047667	0.12931	HSV-2 IgG	TRUE	reported	208	NA	208	0.015115	3.161417
rs10964023	T	G	3.58E-06	-0.19364	0.040373	0.189655	HSV-2 IgG	TRUE	reported	208	NA	208	0.011525	2.401831
rs10790877	G	A	7.82E-07	0.162333	0.031611	0.525862	HSV-2 IgG	TRUE	reported	208	NA	208	0.013141	2.74304
rs699242	A	G	1.01E-06	-0.03528	0.007215	0.826903	VZV IgG	TRUE	reported	8735	NA	8735	0.000356	3.112982
rs75651344	T	A	2.90E-07	-0.06188	0.012064	0.054108	VZV IgG	TRUE	reported	8735	NA	8735	0.000392	3.42473
rs145339536	A	G	4.91E-06	-0.08322	0.018215	0.022893	VZV IgG	TRUE	reported	8735	NA	8735	0.00031	2.706412
rs12491799	C	T	4.77E-06	0.056987	0.012457	0.074448	VZV IgG	TRUE	reported	8735	NA	8735	0.000448	3.91019
rs374456069	G	A	2.82E-07	-0.06894	0.013425	0.044164	VZV IgG	TRUE	reported	8735	NA	8735	0.000401	3.505486
rs573635286	T	G	3.00E-08	0.052227	0.009425	0.190919	VZV IgG	TRUE	reported	8735	NA	8735	0.000843	7.365392
rs1766	G	A	1.14E-11	0.037336	0.005501	0.470281	VZV IgG	TRUE	reported	8735	NA	8735	0.000695	6.069577
rs9295829	G	A	5.02E-07	-0.0306	0.006089	0.274356	VZV IgG	TRUE	reported	8735	NA	8735	0.000373	3.257267

rs62473135	C	G	3.18E-06	-0.04199	0.009013	0.102007	VZV IgG	TRUE	reported	8735	NA	8735	0.000323	2.821613
rs145749545	T	G	4.30E-07	-0.10555	0.02088	0.017379	VZV IgG	TRUE	reported	8735	NA	8735	0.000381	3.324255
rs78192503	A	T	4.19E-06	-0.02745	0.005965	0.336074	VZV IgG	TRUE	reported	8735	NA	8735	0.000336	2.937549
rs77591080	A	G	1.15E-06	-0.08702	0.017891	0.023356	VZV IgG	TRUE	reported	8735	NA	8735	0.000345	3.017745
rs76940747	T	C	4.84E-06	-0.10655	0.023307	0.014042	VZV IgG	TRUE	reported	8735	NA	8735	0.000314	2.746099
rs143711261	T	C	1.51E-07	-0.1454	0.027692	0.010131	VZV IgG	TRUE	reported	8735	NA	8735	0.000424	3.704763
rs10438342	A	G	3.66E-06	0.026839	0.005797	0.343837	VZV IgG	TRUE	reported	8735	NA	8735	0.000325	2.839497
rs117403751	A	T	2.12E-06	-0.08866	0.018698	0.021719	VZV IgG	TRUE	reported	8735	NA	8735	0.000334	2.918253
rs190809282	G	A	4.73E-06	-0.1246	0.027227	0.010097	VZV IgG	TRUE	reported	8735	NA	8735	0.00031	2.711037
rs77266793	T	A	2.58E-06	-0.11923	0.025358	0.011914	VZV IgG	TRUE	reported	8735	NA	8735	0.000335	2.923898
rs7530647	G	A	4.48E-06	-0.1096	0.023891	0.199026	Herpes zoster	TRUE	reported	5488	396478	401966	0.00383	1545.344
rs4451553	C	T	4.79E-06	-0.09344	0.020429	0.326602	Herpes zoster	TRUE	reported	5488	396478	401966	0.00384	1549.656
rs79408779	C	T	1.00E-06	0.182921	0.037398	0.079672	Herpes zoster	TRUE	reported	5488	396478	401966	0.004907	1982.113
rs6446362	G	A	3.61E-06	0.100464	0.021686	0.734572	Herpes zoster	TRUE	reported	5488	396478	401966	0.003936	1588.298
rs81302	A	G	1.72E-06	-0.0955	0.019964	0.359274	Herpes zoster	TRUE	reported	5488	396478	401966	0.004199	1694.8
rs77971737	C	T	2.41E-06	-0.37978	0.080542	0.013165	Herpes zoster	TRUE	reported	5488	396478	401966	0.003748	1512.078
rs7766156	C	G	1.41E-07	-0.11971	0.022743	0.228011	Herpes zoster	TRUE	reported	5488	396478	401966	0.005045	2038.038
rs9266782	G	C	6.08E-14	-0.30811	0.041047	0.053959	Herpes zoster	TRUE	reported	5488	396478	401966	0.009692	3934.061
rs62482377	G	C	1.78E-06	-0.14977	0.031354	0.101248	Herpes zoster	TRUE	reported	5488	396478	401966	0.004082	1647.557
rs8181185	A	C	5.07E-07	0.099107	0.019728	0.619678	Herpes zoster	TRUE	reported	5488	396478	401966	0.00463	1869.628
rs117669514	A	G	2.25E-06	-0.32712	0.06916	0.018305	Herpes zoster	TRUE	reported	5488	396478	401966	0.003846	1551.838
rs2965306	A	G	3.98E-06	0.125661	0.027244	0.858743	Herpes zoster	TRUE	reported	5488	396478	401966	0.003831	1545.818
rs13313427	G	A	3.28E-06	0.129858	0.027913	0.865468	Herpes zoster	TRUE	reported	5488	396478	401966	0.003927	1584.674
rs8054806	G	T	2.05E-06	0.127262	0.0268	0.157895	Herpes zoster	TRUE	reported	5488	396478	401966	0.004307	1738.696
rs59257919	T	C	2.10E-06	0.213345	0.04498	0.099782	EBV infections	TRUE	reported	2979	400974	403953	0.008177	3330.331
rs10174588	G	C	1.59E-06	0.129422	0.026967	0.38396	EBV infections	TRUE	reported	2979	400974	403953	0.007924	3226.449
rs2618374	C	T	3.28E-06	0.132658	0.028514	0.706276	EBV infections	TRUE	reported	2979	400974	403953	0.007301	2971.133
rs28529232	A	G	3.21E-06	0.223307	0.047955	0.08723	EBV infections	TRUE	reported	2979	400974	403953	0.007941	3233.359
rs318497	G	A	5.45E-12	0.183922	0.026681	0.423459	EBV infections	TRUE	reported	2979	400974	403953	0.016517	6784.235
rs192741093	G	A	6.78E-08	-0.53617	0.099347	0.014459	EBV infections	TRUE	reported	2979	400974	403953	0.008193	3337.015
rs3130169	C	T	8.79E-07	0.180085	0.036626	0.156721	EBV infections	TRUE	reported	2979	400974	403953	0.008572	3492.618
rs12358176	C	T	4.32E-06	-0.1247	0.027135	0.373569	EBV infections	TRUE	reported	2979	400974	403953	0.007277	2961.283
rs35170178	C	G	4.35E-06	0.178974	0.038958	0.137362	EBV infections	TRUE	reported	2979	400974	403953	0.007591	3089.891
rs11620121	G	C	2.56E-06	0.125217	0.026622	0.428083	EBV infections	TRUE	reported	2979	400974	403953	0.007677	3125.312
rs143176687	G	C	1.99E-06	1.04499	0.219796	0.005247	EBV infections	TRUE	reported	2979	400974	403953	0.0114	4658.051
rs12598357	A	G	1.63E-07	0.136845	0.02613	0.527544	EBV infections	TRUE	reported	2979	400974	403953	0.009335	3806.359
rs112242506	A	C	1.15E-06	0.392308	0.080658	0.030941	EBV infections	TRUE	reported	2979	400974	403953	0.009229	3762.891
rs10468923	T	C	4.06E-07	-0.14239	0.028107	0.313117	EBV infections	TRUE	reported	2979	400974	403953	0.008721	3553.95
rs76206169	G	A	8.66E-07	-1.4323	0.291127	0.001075	EBV infections	TRUE	reported	2979	400974	403953	0.004405	1787.397
rs56253436	A	G	4.63E-06	-0.18747	0.040925	0.107099	EBV infections	TRUE	reported	2979	400974	403953	0.006721	2733.485
rs6895504	C	T	4.14E-06	0.147586	0.031858	0.206367	EBNA1	TRUE	reported	914	NA	914	0.007135	6.553674
rs4555924	G	A	1.68E-06	0.190281	0.039479	0.132821	EBNA1	TRUE	reported	914	NA	914	0.008341	7.670588

rs74951723	T	A	2.99E-14	0.287162	0.037178	0.135016	EBNA1	TRUE	reported	914	NA	914	0.019261	17.91095
rs6927022	G	A	9.71E-12	-0.17648	0.025573	0.431394	EBNA1	TRUE	reported	914	NA	914	0.01528	14.15153
rs10226349	T	C	1.29E-06	0.225191	0.046207	0.086169	EBNA1	TRUE	reported	914	NA	914	0.007986	7.3422
rs17452718	G	T	3.93E-06	-0.17758	0.038243	0.133919	EBNA1	TRUE	reported	914	NA	914	0.007315	6.720739
rs530411	T	C	5.37E-07	0.139879	0.027704	0.34742	EBNA1	TRUE	reported	914	NA	914	0.008872	8.163732
rs59217282	T	C	2.89E-06	-0.19326	0.041048	0.118002	EBNA1	TRUE	reported	914	NA	914	0.007774	7.14565
rs10158978	A	T	1.86E-06	-0.12683	0.026434	0.192227	VCA	TRUE	reported	956	NA	956	0.004996	4.789923
rs9876198	T	C	5.00E-07	-0.10877	0.021489	0.390756	VCA	TRUE	reported	956	NA	956	0.005633	5.403893
rs245064	T	C	4.39E-06	-0.10073	0.021808	0.425945	VCA	TRUE	reported	956	NA	956	0.004962	4.757426
rs6556882	C	T	1.04E-06	-0.10921	0.022216	0.369748	VCA	TRUE	reported	956	NA	956	0.005558	5.33227
rs6985207	C	A	1.49E-06	-0.10444	0.021562	0.434874	VCA	TRUE	reported	956	NA	956	0.005362	5.142574
rs2163916	A	G	4.78E-06	-0.1212	0.026344	0.209034	VCA	TRUE	reported	956	NA	956	0.004858	4.656953
rs114227815	C	G	4.99E-06	0.33185	0.072587	0.02655	CMV IgG	TRUE	reported	5010	NA	5010	0.005692	28.67051
rs146990284	A	G	1.18E-06	0.416181	0.085515	0.016825	CMV IgG	TRUE	reported	5010	NA	5010	0.00573	28.86294
rs138792449	T	A	1.72E-06	0.475925	0.099346	0.012261	CMV IgG	TRUE	reported	5010	NA	5010	0.005486	27.62668
rs10028968	A	T	9.52E-07	-0.11221	0.022856	0.38811	CMV IgG	TRUE	reported	5010	NA	5010	0.00598	30.12671
rs7715384	T	C	4.93E-06	-0.15364	0.033587	0.14173	CMV IgG	TRUE	reported	5010	NA	5010	0.005743	28.92459
rs7761068	G	A	1.25E-06	-0.11157	0.022979	0.38685	CMV IgG	TRUE	reported	5010	NA	5010	0.005905	29.74728
rs12214648	C	A	2.33E-06	0.123349	0.026082	0.24473	CMV IgG	TRUE	reported	5010	NA	5010	0.005625	28.32727
rs10269595	G	T	4.80E-06	0.1017	0.022206	0.45381	CMV IgG	TRUE	reported	5010	NA	5010	0.005127	25.80991
rs2467112	T	C	4.53E-06	-0.12442	0.027094	0.23559	CMV IgG	TRUE	reported	5010	NA	5010	0.005575	28.07748
rs6562581	C	G	4.22E-06	-0.19539	0.042414	0.074147	CMV IgG	TRUE	reported	5010	NA	5010	0.005242	26.38864
rs59559055	A	G	3.15E-06	-0.14299	0.030635	0.15722	CMV IgG	TRUE	reported	5010	NA	5010	0.005418	27.28307
rs61825717	A	G	1.94E-07	1.05566	0.20282	0.033877	CMV infections	TRUE	reported	487	411593	412080	0.072948	32425.72
rs13156302	C	T	1.21E-06	0.39166	0.080679	0.203624	CMV infections	TRUE	reported	487	411593	412080	0.04975	21574.33
rs118172547	A	G	4.42E-06	1.04225	0.22704	0.026694	CMV infections	TRUE	reported	487	411593	412080	0.056446	24651.86
rs117479615	A	G	1.88E-06	1.03288	0.216721	0.029653	CMV infections	TRUE	reported	487	411593	412080	0.061395	26954.17
rs12438477	C	A	4.32E-06	-0.29335	0.063835	0.357028	CMV infections	TRUE	reported	487	411593	412080	0.03951	16950.75
rs75050044	G	C	1.24E-06	-0.1043	0.021509	0.010264	HHV-6 IgG	TRUE	reported	8735	NA	8735	0.000221	1.930497
rs4360526	T	C	1.67E-06	0.031289	0.006532	0.124368	HHV-6 IgG	TRUE	reported	8735	NA	8735	0.000213	1.862491
rs142041262	T	G	1.51E-06	-0.07364	0.015309	0.020709	HHV-6 IgG	TRUE	reported	8735	NA	8735	0.00022	1.92124
rs2799671	T	C	8.31E-07	-0.06991	0.014187	0.024306	HHV-6 IgG	TRUE	reported	8735	NA	8735	0.000232	2.024935
rs80064372	G	A	3.05E-06	-0.0624	0.013369	0.027368	HHV-6 IgG	TRUE	reported	8735	NA	8735	0.000207	1.810621
rs4372072	C	T	1.23E-06	0.089662	0.018486	0.985853	HHV-6 IgG	TRUE	reported	8735	NA	8735	0.000224	1.958782
rs77328788	T	C	1.88E-06	0.030972	0.006499	0.126841	HHV-6 IgG	TRUE	reported	8735	NA	8735	0.000212	1.856045
rs79076035	C	T	1.01E-07	-0.0909	0.017069	0.016545	HHV-6 IgG	TRUE	reported	8735	NA	8735	0.000269	2.348742
rs12280629	G	A	4.68E-06	-0.04209	0.009193	0.064805	HHV-6 IgG	TRUE	reported	8735	NA	8735	0.000215	1.875701
rs118159495	T	C	3.70E-06	-0.07499	0.016204	0.018203	HHV-6 IgG	TRUE	reported	8735	NA	8735	0.000201	1.755698
rs17719679	A	G	6.75E-07	-0.05694	0.01146	0.037951	HHV-6 IgG	TRUE	reported	8735	NA	8735	0.000237	2.067787
rs79457971	A	G	4.49E-06	-0.05386	0.011742	0.035332	HHV-6 IgG	TRUE	reported	8735	NA	8735	0.000198	1.727505
rs11844740	C	T	4.59E-06	-0.02018	0.004404	0.56486	HHV-6 IgG	TRUE	reported	8735	NA	8735	0.0002	1.749284
rs116845865	A	G	6.88E-07	-0.10539	0.021228	0.010652	HHV-6 IgG	TRUE	reported	8735	NA	8735	0.000234	2.045026

rs60297852	G	A	2.65E-07	-0.05794	0.011257	0.038113	HHV-6 IgG	TRUE	reported	8735	NA	8735	0.000246	2.15002
rs6070170	C	G	7.34E-07	-0.02176	0.004393	0.499764	HHV-6 IgG	TRUE	reported	8735	NA	8735	0.000237	2.067332
rs116323441	T	C	3.82E-06	-0.05536	0.01198	0.019725	HHV-7 IgG	TRUE	reported	8735	NA	8735	0.000119	1.03511
rs75180372	A	G	3.83E-06	-0.03647	0.007893	0.04606	HHV-7 IgG	TRUE	reported	8735	NA	8735	0.000117	1.020702
rs13015747	T	C	3.44E-06	-0.04275	0.009208	0.033085	HHV-7 IgG	TRUE	reported	8735	NA	8735	0.000117	1.021054
rs76147964	T	C	1.59E-06	-0.05849	0.012187	0.019016	HHV-7 IgG	TRUE	reported	8735	NA	8735	0.000128	1.114951
rs374807575	A	T	1.55E-07	-0.05429	0.010348	0.026286	HHV-7 IgG	TRUE	reported	8735	NA	8735	0.000151	1.318044
rs73207403	A	T	1.68E-06	-0.0405	0.008458	0.040014	HHV-7 IgG	TRUE	reported	8735	NA	8735	0.000126	1.100446
rs116744559	A	G	1.60E-06	-0.05184	0.010804	0.024213	HHV-7 IgG	TRUE	reported	8735	NA	8735	0.000127	1.109258
rs141656540	T	C	1.27E-07	0.043718	0.008276	0.956413	HHV-7 IgG	TRUE	reported	8735	NA	8735	0.000159	1.391816
rs10516029	A	G	4.20E-06	-0.04066	0.008838	0.035793	HHV-7 IgG	TRUE	reported	8735	NA	8735	0.000114	0.996815
rs9366191	A	G	8.12E-07	-0.07305	0.01481	0.012856	HHV-7 IgG	TRUE	reported	8735	NA	8735	0.000135	1.18303
rs76325595	A	T	4.97E-07	-0.04467	0.008886	0.036882	HHV-7 IgG	TRUE	reported	8735	NA	8735	0.000142	1.238443
rs9353859	G	T	3.46E-07	0.017809	0.003495	0.34549	HHV-7 IgG	TRUE	reported	8735	NA	8735	0.000143	1.252856
rs113407243	C	T	4.73E-06	-0.02972	0.006495	0.083063	HHV-7 IgG	TRUE	reported	8735	NA	8735	0.000135	1.175475
rs77839024	C	A	1.26E-06	-0.03237	0.00668	0.065065	HHV-7 IgG	TRUE	reported	8735	NA	8735	0.000127	1.113135
rs146737611	C	A	1.28E-06	-0.03342	0.0069	0.064733	HHV-7 IgG	TRUE	reported	8735	NA	8735	0.000135	1.180911
rs118182641	A	T	8.68E-07	-0.06514	0.013242	0.015539	HHV-7 IgG	TRUE	reported	8735	NA	8735	0.00013	1.133887
rs72688492	C	T	2.98E-06	-0.02794	0.00598	0.087	HHV-7 IgG	TRUE	reported	8735	NA	8735	0.000124	1.083014
rs189573254	T	G	1.95E-07	-0.05747	0.011043	0.022793	HHV-7 IgG	TRUE	reported	8735	NA	8735	0.000147	1.285234
rs72937918	T	C	8.99E-07	-0.05254	0.010694	0.024614	HHV-7 IgG	TRUE	reported	8735	NA	8735	0.000133	1.157534
rs78492833	A	G	1.37E-06	-0.06142	0.012716	0.017401	HHV-7 IgG	TRUE	reported	8735	NA	8735	0.000129	1.126707
rs75920137	T	C	2.61E-06	-0.05925	0.01261	0.017424	HHV-7 IgG	TRUE	reported	8735	NA	8735	0.00012	1.050007
rs35348947	G	A	9.95E-07	-0.05445	0.011129	0.02403	HHV-7 IgG	TRUE	reported	8735	NA	8735	0.000139	1.214623
rs11643441	A	G	4.05E-06	-0.03425	0.007432	0.051803	HHV-7 IgG	TRUE	reported	8735	NA	8735	0.000115	1.006645
rs145892114	T	C	4.13E-06	-0.07616	0.016539	0.010102	HHV-7 IgG	TRUE	reported	8735	NA	8735	0.000116	1.013153
rs73387684	C	A	2.42E-06	-0.0376	0.007975	0.045059	HHV-7 IgG	TRUE	reported	8735	NA	8735	0.000122	1.062557
rs78332643	A	T	7.52E-08	-0.08524	0.01585	0.011114	HHV-7 IgG	TRUE	reported	8735	NA	8735	0.00016	1.395093
rs17563060	C	T	4.75E-06	-0.03244	0.00709	0.057902	HHV-7 IgG	TRUE	reported	8735	NA	8735	0.000115	1.00286

Table S2. Details of the instrumental variables after harmonization process.

SNP	effect_alle	other_allel	effect_alle	other_allel	beta.expo	beta.outc	eaf.expos	eaf.outcor	remove	palindrom	ambiguou	id.outcom	se.outcom	samplesiz	pval.outc	outcome	mr_keep.c	pval.expos	se.exposu	exposure	mr_keep.e	pval_origi	id.exposur	action	mr_keep
rs10234639	G	T	G	T	0.12015	0.0143	0.28097	NA	FALSE	FALSE	FALSE	ieu-a-118	0.0144	46351	0.3188	Autism Sp	TRUE	4.55E-06	0.02621	HSV infect	TRUE	reported	XPdJog	2	TRUE
rs10961236	A	G	A	G	0.12574	-0.0042	0.27059	NA	FALSE	FALSE	FALSE	ieu-a-118	0.015	46351	0.7778	Autism Sp	TRUE	2.01E-06	0.02646	HSV infect	TRUE	reported	XPdJog	2	TRUE
rs12457005	C	T	C	T	0.28939	-0.0101	0.05023	NA	FALSE	FALSE	FALSE	ieu-a-118	0.029	46351	0.7287	Autism Sp	TRUE	2.32E-07	0.05596	HSV infect	TRUE	reported	XPdJog	2	TRUE
rs148444866	A	G	A	G	0.31358	-0.10649	0.0338	NA	FALSE	FALSE	FALSE	ieu-a-118	0.0678	46351	0.1166	Autism Sp	TRUE	3.79E-06	0.06784	HSV infect	TRUE	reported	XPdJog	2	TRUE
rs2004786	T	G	T	G	-0.11469	-0.0082	0.30566	NA	FALSE	FALSE	FALSE	ieu-a-118	0.0147	46351	0.5776	Autism Sp	TRUE	4.62E-06	0.02503	HSV infect	TRUE	reported	XPdJog	2	TRUE
rs4716482	C	A	C	A	-0.11575	-0.0246	0.4902	NA	FALSE	FALSE	FALSE	ieu-a-118	0.0141	46351	0.08172	Autism Sp	TRUE	7.20E-07	0.02336	HSV infect	TRUE	reported	XPdJog	2	TRUE
rs59142651	A	G	A	G	-0.45146	-0.096	0.01261	NA	FALSE	FALSE	FALSE	ieu-a-118	0.0441	46351	0.02949	Autism Sp	TRUE	4.16E-06	0.09808	HSV infect	TRUE	reported	XPdJog	2	TRUE
rs71428759	G	T	G	T	0.32306	-0.0178	0.03313	NA	FALSE	FALSE	FALSE	ieu-a-118	0.048	46351	0.7103	Autism Sp	TRUE	2.39E-06	0.06849	HSV infect	TRUE	reported	XPdJog	2	TRUE
rs75710096	G	A	G	A	0.23419	-0.011	0.06047	NA	FALSE	FALSE	FALSE	ieu-a-118	0.0258	46351	0.6703	Autism Sp	TRUE	3.73E-06	0.05063	HSV infect	TRUE	reported	XPdJog	2	TRUE
rs77231357	G	A	G	A	0.2271	-0.0013	0.07164	NA	FALSE	FALSE	FALSE	ieu-a-118	0.0342	46351	0.9704	Autism Sp	TRUE	1.92E-06	0.0477	HSV infect	TRUE	reported	XPdJog	2	TRUE
rs9263969	T	C	T	C	-0.13625	-0.009	0.22516	NA	FALSE	FALSE	FALSE	ieu-a-118	0.0168	46351	0.593	Autism Sp	TRUE	6.88E-07	0.02744	HSV infect	TRUE	reported	XPdJog	2	TRUE
rs10234639	G	T	G	T	0.12015	0.0079	0.28097	NA	FALSE	FALSE	FALSE	z10Ukz	0.0097	225534	0.4164	Attention	TRUE	4.55E-06	0.02621	HSV infect	TRUE	reported	XPdJog	2	TRUE
rs12457005	C	T	C	T	0.28939	-0.0242	0.05023	NA	FALSE	FALSE	FALSE	z10Ukz	0.0253	225534	0.339	Attention	TRUE	2.32E-07	0.05596	HSV infect	TRUE	reported	XPdJog	2	TRUE
rs148444866	A	G	A	G	0.31358	0.035	0.0338	NA	FALSE	FALSE	FALSE	z10Ukz	0.0423	225534	0.4074	Attention	TRUE	3.79E-06	0.06784	HSV infect	TRUE	reported	XPdJog	2	TRUE
rs2004786	T	G	T	G	-0.11469	0.0122	0.30566	NA	FALSE	FALSE	FALSE	z10Ukz	0.01	225534	0.2224	Attention	TRUE	4.62E-06	0.02503	HSV infect	TRUE	reported	XPdJog	2	TRUE
rs4716482	C	A	C	A	-0.11575	0.0156	0.4902	NA	FALSE	FALSE	FALSE	z10Ukz	0.0096	225534	0.1044	Attention	TRUE	7.20E-07	0.02336	HSV infect	TRUE	reported	XPdJog	2	TRUE
rs59142651	A	G	A	G	-0.45146	-0.0211	0.01261	NA	FALSE	FALSE	FALSE	z10Ukz	0.0287	225534	0.4639	Attention	TRUE	4.16E-06	0.09808	HSV infect	TRUE	reported	XPdJog	2	TRUE
rs71428759	G	T	G	T	0.32306	-0.0022	0.03313	NA	FALSE	FALSE	FALSE	z10Ukz	0.0318	225534	0.9455	Attention	TRUE	2.39E-06	0.06849	HSV infect	TRUE	reported	XPdJog	2	TRUE
rs75710096	G	A	G	A	0.23419	0.0236	0.06047	NA	FALSE	FALSE	FALSE	z10Ukz	0.0177	225534	0.1824	Attention	TRUE	3.73E-06	0.05063	HSV infect	TRUE	reported	XPdJog	2	TRUE
rs77231357	G	A	G	A	0.2271	-0.0149	0.07164	NA	FALSE	FALSE	FALSE	z10Ukz	0.0214	225534	0.4857	Attention	TRUE	1.92E-06	0.0477	HSV infect	TRUE	reported	XPdJog	2	TRUE
rs9263969	T	C	T	C	-0.13625	0.005	0.22516	NA	FALSE	FALSE	FALSE	z10Ukz	0.012	225534	0.6765	Attention	TRUE	6.88E-07	0.02744	HSV infect	TRUE	reported	XPdJog	2	TRUE
rs10234639	G	T	G	T	0.12015	-0.0142	0.28097	NA	FALSE	FALSE	FALSE	a8axBA	0.0275	14307	0.605	Tourette s	TRUE	4.55E-06	0.02621	HSV infect	TRUE	reported	XPdJog	2	TRUE
rs10961236	A	G	A	G	0.12574	0.0188	0.27059	NA	FALSE	FALSE	FALSE	a8axBA	0.028	14307	0.5016	Tourette s	TRUE	2.01E-06	0.02646	HSV infect	TRUE	reported	XPdJog	2	TRUE
rs12457005	C	T	C	T	0.28939	-0.0384	0.05023	NA	FALSE	FALSE	FALSE	a8axBA	0.0509	14307	0.4506	Tourette s	TRUE	2.32E-07	0.05596	HSV infect	TRUE	reported	XPdJog	2	TRUE
rs148444866	A	G	A	G	0.31358	0.0283	0.0338	NA	FALSE	FALSE	FALSE	a8axBA	0.1169	14307	0.8088	Tourette s	TRUE	3.79E-06	0.06784	HSV infect	TRUE	reported	XPdJog	2	TRUE
rs2004786	T	G	T	G	-0.11469	-0.0202	0.30566	NA	FALSE	FALSE	FALSE	a8axBA	0.0275	14307	0.4622	Tourette s	TRUE	4.62E-06	0.02503	HSV infect	TRUE	reported	XPdJog	2	TRUE
rs4716482	C	A	C	A	-0.11575	0.0168	0.4902	NA	FALSE	FALSE	FALSE	a8axBA	0.0261	14307	0.5215	Tourette s	TRUE	7.20E-07	0.02336	HSV infect	TRUE	reported	XPdJog	2	TRUE
rs59142651	A	G	A	G	-0.45146	-0.0213	0.01261	NA	FALSE	FALSE	FALSE	a8axBA	0.0717	14307	0.7665	Tourette s	TRUE	4.16E-06	0.09808	HSV infect	TRUE	reported	XPdJog	2	TRUE
rs71428759	G	T	G	T	0.32306	0.0901	0.03313	NA	FALSE	FALSE	FALSE	a8axBA	0.092	14307	0.3276	Tourette s	TRUE	2.39E-06	0.06849	HSV infect	TRUE	reported	XPdJog	2	TRUE
rs75710096	G	A	G	A	0.23419	0.034	0.06047	NA	FALSE	FALSE	FALSE	a8axBA	0.0492	14307	0.4893	Tourette s	TRUE	3.73E-06	0.05063	HSV infect	TRUE	reported	XPdJog	2	TRUE
rs77231357	G	A	G	A	0.2271	0.0191	0.07164	NA	FALSE	FALSE	FALSE	a8axBA	0.0646	14307	0.7674	Tourette s	TRUE	1.92E-06	0.0477	HSV infect	TRUE	reported	XPdJog	2	TRUE
rs117669514	G	A	G	A	-0.32712	0.0897	0.0183	NA	FALSE	FALSE	FALSE	ieu-a-118	0.0612	46351	0.1428	Autism Sp	TRUE	2.25E-06	0.06916	Herpes zo	TRUE	reported	7eqbIM	2	TRUE
rs13313427	A	G	A	G	0.12986	0.0167	0.86547	NA	FALSE	FALSE	FALSE	ieu-a-118	0.0191	46351	0.383	Autism Sp	TRUE	3.28E-06	0.02791	Herpes zo	TRUE	reported	7eqbIM	2	TRUE
rs2965306	G	A	G	A	0.12566	0.0058	0.85874	NA	FALSE	FALSE	FALSE	ieu-a-118	0.0208	46351	0.7798	Autism Sp	TRUE	3.98E-06	0.02724	Herpes zo	TRUE	reported	7eqbIM	2	TRUE
rs4451553	T	C	T	C	-0.09344	0.004	0.3266	NA	FALSE	FALSE	FALSE	ieu-a-118	0.0147	46351	0.7868	Autism Sp	TRUE	4.79E-06	0.02043	Herpes zo	TRUE	reported	7eqbIM	2	TRUE
rs6446362	A	G	A	G	0.10046	0.0047	0.73457	NA	FALSE	FALSE	FALSE	ieu-a-118	0.0147	46351	0.7484	Autism Sp	TRUE	3.61E-06	0.02169	Herpes zo	TRUE	reported	7eqbIM	2	TRUE
rs7530647	A	G	A	G	-0.1096	0.0058	0.19903	NA	FALSE	FALSE	FALSE	ieu-a-118	0.0159	46351	0.715	Autism Sp	TRUE	4.48E-06	0.02389	Herpes zo	TRUE	reported	7eqbIM	2	TRUE
rs8054806	T	G	T	G	0.12726	-0.0136	0.1579	NA	FALSE	FALSE	FALSE	ieu-a-118	0.0178	46351	0.4448	Autism Sp	TRUE	2.05E-06	0.0268	Herpes zo	TRUE	reported	7eqbIM	2	TRUE
rs81302	G	A	G	A	-0.0955	-0.0279	0.35927	NA	FALSE	FALSE	FALSE	ieu-a-118	0.0149	46351	0.0609	Autism Sp	TRUE	1.72E-06	0.01996	Herpes zo	TRUE	reported	7eqbIM	2	TRUE
rs8181185	C	A	C	A	0.09911	-0.0368	0.61968	NA	FALSE	FALSE	FALSE	ieu-a-118	0.0142	46351	0.00966	Autism Sp	TRUE	5.07E-07	0.01973	Herpes zo	TRUE	reported	7eqbIM	2	TRUE
rs117669514	G	A	G	A	-0.32712	0.0248	0.0183	NA	FALSE	FALSE	FALSE	G33A3i	0.0302	225534	0.413	Attention	TRUE	2.25E-06	0.06916	Herpes zo	TRUE	reported	8eqbIM	2	TRUE
rs13313427	A	G	A	G	0.12986	-0.0043	0.86547	NA	FALSE	FALSE	FALSE	G33A3i	0.013	225534	0.7388	Attention	TRUE	3.28E-06	0.02791	Herpes zo	TRUE	reported	9eqbIM	2	TRUE
rs2965306	G	A	G	A	0.12566	-0.0037	0.85874	NA	FALSE	FALSE	FALSE	G33A3i	0.0134	225534	0.7809	Attention	TRUE	3.98E-06	0.02724	Herpes zo	TRUE	reported	10eqbIM	2	TRUE
rs4451553	T	C	T	C	-0.09344	0.001	0.3266	NA	FALSE	FALSE	FALSE	G33A3i	0.0101	225534	0.9196	Attention	TRUE	4.79E-06	0.02043	Herpes zo	TRUE	reported	11eqbIM	2	TRUE
rs6446362	A	G	A	G	0.10046	0.0024	0.73457	NA	FALSE	FALSE	FALSE	G33A3i	0.0098	225534	0.8085	Attention	TRUE	3.61E-06	0.02169	Herpes zo	TRUE	reported	12eqbIM	2	TRUE
rs7530647	A	G	A	G	-0.1096	0.0056	0.19903	NA	FALSE	FALSE	FALSE	G33A3i	0.0107	225534	0.6012	Attention	TRUE	4.48E-06	0.02389	Herpes zo	TRUE	reported	13eqbIM	2	TRUE
rs8054806	T	G	T	G	0.12726	0.0095	0.1579	NA	FALSE	FALSE	FALSE	G33A3i	0.012	225534	0.4271	Attention	TRUE	2.05E-06	0.0268	Herpes zo	TRUE	reported	14eqbIM	2	TRUE
rs81302	G	A	G	A	-0.0955	0.009	0.35927	NA	FALSE	FALSE	FALSE	G33A3i	0.01	225534	0.3672	Attention	TRUE	1.72E-06	0.01996	Herpes zo	TRUE	reported	15eqbIM	2	TRUE
rs8181185	C	A	C	A	0.09911	0.0044	0.61968	NA	FALSE	FALSE	FALSE	G33A3i	0.0103	225534	0.6718	Attention	TRUE	5.07E-07	0.01973	Herpes zo	TRUE	reported	16eqbIM	2	TRUE
rs117669514	G	A	G	A	-0.32712	-0.1856	0.0183	NA	FALSE	FALSE	FALSE	zwPrjc	0.1342	14307	0.1668	Tourette s	TRUE	2.25E-06	0.06916	Herpes zo	TRUE	reported	17eqbIM	2	TRUE
rs13313427	A	G	A	G	0.12986	-0.0292	0.86547	NA	FALSE	FALSE	FALSE	zwPrjc	0.0369	14307	0.4296	Tourette s	TRUE	3.28E-06	0.02791	Herpes zo	TRUE	reported	18eqbIM	2	TRUE
rs2965306	G	A	G	A	0.12566	-0.0656	0.85874	NA	FALSE	FALSE	FALSE	zwPrjc	0.039	14307	0.09256	Tourette s	TRUE	3.98E-06	0.02724	Herpes zo	TRUE	reported	19eqbIM	2	TRUE
rs4451553	T	C	T	C	-0.09344	0.0004	0.3266	NA	FALSE	FALSE	FALSE	zwPrjc	0.027	14307	0.9888	Tourette s	TRUE	4.79E-06	0.02043	Herpes zo	TRUE	reported	20eqbIM	2	TRUE
rs6446362	A	G	A	G	0.10046	-0.0386	0.73457	NA	FALSE	FALSE	FALSE	zwPrjc	0.0271	14307	0.1542	Tourette s	TRUE	3.61E-06	0.02169	Herpes zo	TRUE	reported	21eqbIM	2	TRUE
rs7530647	A	G	A	G	-0.1096	0.0198	0.19903	NA	FALSE	FALSE	FALSE	zwPrjc	0.031	14307	0.5232	Tourette s	TRUE	4.48E-06	0.02389	Herpes zo	TRUE	reported	22eqbIM	2	TRUE
rs77971737	T	C	T	C	-0.37978	0.0872	0.01316	NA	FALSE	FALSE	FALSE	zwPrjc	0.1291	14307	0.4998	Tourette s	TRUE	2.41E-06	0.08054	Herpes zo	TRUE	reported	23eqbIM	2	TRUE
rs8054806	T	G	T	G	0.12726	0.0212	0.1579	NA	FALSE	FALSE	FALSE	zwPrjc	0.0325	14307	0.5133	Tourette s	TRUE	2.05E-06	0.0268	Herpes zo	TRUE	reported	24eqbIM	2	TRUE
rs81302	G	A	G	A	-0.0955	0.0067	0.35927	NA	FALSE	FALSE	FALSE	zwPrjc	0.0277	14307	0.8082	Tourette s	TRUE	1.72E-06	0.01996	Herpes zo	TRUE	reported	25eqbIM	2	TRUE
rs8181185	C	A	C	A	0.09911	0.0059	0.61968	NA	FALSE	FALSE	FALSE	zwPrjc	0.0264	14307	0.8221	Tourette s	TRUE	5.07E-07	0.01973	Herpes zo	TRUE	reported	26eqbIM	2	TRUE
rs10468923	C	T	C	T	-0.14239	-0.0054	0.31312	NA	FALSE	FALSE	FALSE	ieu-a-118	0.0167	46351	0.745	Autism Sp	TRUE	4.06E-07	0.02811	EBV infect	TRUE	reported	htRGGG	2	TRUE
rs112242506	C	A	C	A	0.39231	0.06	0.03094	NA	FALSE	FALSE	FALSE	ieu-a-118	0.057	46351	0.2923	Autism Sp	TRUE	1.15E-06	0.08066	EBV infect	TRUE	reported	htRGGG	2	TRUE
rs12358176	T	C	T	C	-0.1247	0.0117	0.37357	NA	FALSE	FALSE	FALSE	ieu-a-118	0.0152	46351	0.4413	Autism Sp	TRUE	4.32E-06	0.02713	EBV infect	TRUE	reported	htRGGG	2	TRUE
rs12598357	G	A	G	A	0.13685	0.0136	0.52754	NA	FALSE	FALSE	FALSE	ieu-a-118	0.0143	46351	0.3416	Autism Sp	TRUE	1.63E-07	0.02613	EBV infect	TRUE	reported	htRGGG	2	TRUE
rs192741093	A	G	A	G	-0.53617	0.0961	0.01446	NA	FALSE	FALSE	FALSE	ieu-a-118	0.0519	46351	0.06424	Autism Sp	TRUE	6.78E-08	0.09935	EBV infect	TRUE	reported	htRGGG	2	TRUE
rs2618374	T	C	T	C	0.13266	-0.0101	0.70628	NA	FALSE	FALSE	FALSE	ieu-a-118	0.0173	46351	0.5587	Autism Sp	TRUE	3.28E-06	0.02851	EBV infect	TRUE	reported	htRGGG	2	TRUE
rs28529232	G	A	G	A	0.22331	0.0763	0.08723	NA	FALSE	FALSE	FALSE	ieu-a-118	0.0212	46351	0.00032	Autism Sp	TRUE	3.21E-06	0.04795	EBV infect	TRUE	reported	htRGGG	2	TRUE
rs3130169	T	C	T	C	0.18009	0.0202	0.15672	NA	FALSE	FALSE	FALSE	ieu-a-118	0.0215	46351	0.346	Autism Sp	TRUE	8.79E-07	0.03663	EBV infect	TRUE	reported	htRGGG	2	TRUE
rs318497	A	G	A	G	0.18392	0.0152	0.42346	NA	FALSE	FALSE	FALSE	ieu-a-118	0.0147	46351	0.3033	Autism Sp	TRUE	5.45E-12	0.02668	EBV infect	TRUE	reported	htRGGG	2	TRUE

rs56253436	G	A	G	A	-0.18747	-0.0024	0.1071	NA	FALSE	FALSE	FALSE	ieu-a-118	0.0285	46351	0.9334	Autism Sp	TRUE	4.63E-06	0.04092	EBV infect	TRUE	reported	htRGGG	2	TRUE
rs59257919	C	T	C	T	0.21335	0.0391	0.09978	NA	FALSE	FALSE	FALSE	ieu-a-118	0.0227	46351	0.0843	Autism Sp	TRUE	2.10E-06	0.04498	EBV infect	TRUE	reported	htRGGG	2	TRUE
rs76206169	A	G	A	G	-1.4323	0.0174	0.00107	NA	FALSE	FALSE	FALSE	ieu-a-118	0.0582	46351	0.7653	Autism Sp	TRUE	8.66E-07	0.29113	EBV infect	TRUE	reported	htRGGG	2	TRUE
rs10468923	C	T	C	T	-0.14239	-0.0039	0.31312	NA	FALSE	FALSE	FALSE	3kEFYN	0.0126	225534	0.757	Attention	TRUE	4.06E-07	0.02811	EBV infect	TRUE	reported	htRGGG	2	TRUE
rs12358176	T	C	T	C	-0.1247	-0.0169	0.37357	NA	FALSE	FALSE	FALSE	3kEFYN	0.0104	225534	0.1037	Attention	TRUE	4.32E-06	0.02713	EBV infect	TRUE	reported	htRGGG	2	TRUE
rs12598357	G	A	G	A	0.13685	-0.0034	0.52754	NA	FALSE	FALSE	FALSE	3kEFYN	0.0101	225534	0.733	Attention	TRUE	1.63E-07	0.02613	EBV infect	TRUE	reported	htRGGG	2	TRUE
rs192741093	A	G	A	G	-0.53617	-0.0042	0.01446	NA	FALSE	FALSE	FALSE	3kEFYN	0.0345	225534	0.9041	Attention	TRUE	6.78E-08	0.09935	EBV infect	TRUE	reported	htRGGG	2	TRUE
rs2618374	T	C	T	C	0.13266	-0.017	0.70628	NA	FALSE	FALSE	FALSE	3kEFYN	0.0115	225534	0.1392	Attention	TRUE	3.28E-06	0.02851	EBV infect	TRUE	reported	htRGGG	2	TRUE
rs28529232	G	A	G	A	0.22331	-0.0161	0.08723	NA	FALSE	FALSE	FALSE	3kEFYN	0.0142	225534	0.2586	Attention	TRUE	3.21E-06	0.04795	EBV infect	TRUE	reported	htRGGG	2	TRUE
rs3130169	T	C	T	C	0.18009	-0.013	0.15672	NA	FALSE	FALSE	FALSE	3kEFYN	0.014	225534	0.3513	Attention	TRUE	8.79E-07	0.03663	EBV infect	TRUE	reported	htRGGG	2	TRUE
rs318497	A	G	A	G	0.18392	0.009	0.42346	NA	FALSE	FALSE	FALSE	3kEFYN	0.0097	225534	0.3553	Attention	TRUE	5.45E-12	0.02668	EBV infect	TRUE	reported	htRGGG	2	TRUE
rs56253436	G	A	G	A	-0.18747	-0.0432	0.1071	NA	FALSE	FALSE	FALSE	3kEFYN	0.0169	225534	0.01047	Attention	TRUE	4.63E-06	0.04092	EBV infect	TRUE	reported	htRGGG	2	TRUE
rs59257919	C	T	C	T	0.21335	-0.008	0.09978	NA	FALSE	FALSE	FALSE	3kEFYN	0.0161	225534	0.6188	Attention	TRUE	2.10E-06	0.04498	EBV infect	TRUE	reported	htRGGG	2	TRUE
rs76206169	A	G	A	G	-1.4323	0.0017	0.00107	NA	FALSE	FALSE	FALSE	3kEFYN	0.0333	225534	0.9592	Attention	TRUE	8.66E-07	0.29113	EBV infect	TRUE	reported	htRGGG	2	TRUE
rs10468923	C	T	C	T	-0.14239	0.0164	0.31312	NA	FALSE	FALSE	FALSE	6T3jt2	0.0538	14307	0.7603	Tourette s	TRUE	4.06E-07	0.02811	EBV infect	TRUE	reported	htRGGG	2	TRUE
rs112242506	C	A	C	A	0.39231	-0.015	0.03094	NA	FALSE	FALSE	FALSE	6T3jt2	0.1099	14307	0.8911	Tourette s	TRUE	1.15E-06	0.08066	EBV infect	TRUE	reported	htRGGG	2	TRUE
rs12358176	T	C	T	C	-0.1247	-0.0354	0.37357	NA	FALSE	FALSE	FALSE	6T3jt2	0.0308	14307	0.2498	Tourette s	TRUE	4.32E-06	0.02713	EBV infect	TRUE	reported	htRGGG	2	TRUE
rs12598357	G	A	G	A	0.13685	-0.0019	0.52754	NA	FALSE	FALSE	FALSE	6T3jt2	0.0283	14307	0.9455	Tourette s	TRUE	1.63E-07	0.02613	EBV infect	TRUE	reported	htRGGG	2	TRUE
rs2618374	T	C	T	C	0.13266	0.0382	0.70628	NA	FALSE	FALSE	FALSE	6T3jt2	0.0329	14307	0.245	Tourette s	TRUE	3.28E-06	0.02851	EBV infect	TRUE	reported	htRGGG	2	TRUE
rs28529232	G	A	G	A	0.22331	-0.0288	0.08723	NA	FALSE	FALSE	FALSE	6T3jt2	0.0386	14307	0.4566	Tourette s	TRUE	3.21E-06	0.04795	EBV infect	TRUE	reported	htRGGG	2	TRUE
rs318497	A	G	A	G	0.18392	-0.0132	0.42346	NA	FALSE	FALSE	FALSE	6T3jt2	0.0258	14307	0.6097	Tourette s	TRUE	5.45E-12	0.02668	EBV infect	TRUE	reported	htRGGG	2	TRUE
rs56253436	G	A	G	A	-0.18747	-0.0244	0.1071	NA	FALSE	FALSE	FALSE	6T3jt2	0.0566	14307	0.6671	Tourette s	TRUE	4.63E-06	0.04092	EBV infect	TRUE	reported	htRGGG	2	TRUE
rs59257919	C	T	C	T	0.21335	-0.061	0.09978	NA	FALSE	FALSE	FALSE	6T3jt2	0.0387	14307	0.1145	Tourette s	TRUE	2.10E-06	0.04498	EBV infect	TRUE	reported	htRGGG	2	TRUE
rs76206169	A	G	A	G	-1.4323	-0.0238	0.00107	NA	FALSE	FALSE	FALSE	6T3jt2	0.11	14307	0.8289	Tourette s	TRUE	8.66E-07	0.29113	EBV infect	TRUE	reported	htRGGG	2	TRUE
rs10269595	G	T	G	T	0.1017	-0.0075	0.45381	NA	FALSE	FALSE	FALSE	ieu-a-118	0.0141	46351	0.5952	Autism Sp	TRUE	4.80E-06	0.02221	CMV IgG	TRUE	reported	igd	2	TRUE
rs12214648	C	A	C	A	0.12335	0.0097	0.24473	NA	FALSE	FALSE	FALSE	ieu-a-118	0.0167	46351	0.5634	Autism Sp	TRUE	2.33E-06	0.02608	CMV IgG	TRUE	reported	igd	2	TRUE
rs146990284	A	G	A	G	0.41618	0.1415	0.01683	NA	FALSE	FALSE	FALSE	ieu-a-118	0.0585	46351	0.01561	Autism Sp	TRUE	1.18E-06	0.08552	CMV IgG	TRUE	reported	igd	2	TRUE
rs2467112	T	C	T	C	-0.12442	-0.0051	0.23559	NA	FALSE	FALSE	FALSE	ieu-a-118	0.0167	46351	0.7599	Autism Sp	TRUE	4.53E-06	0.02709	CMV IgG	TRUE	reported	igd	2	TRUE
rs59559055	A	G	A	G	-0.14299	0.0229	0.15722	NA	FALSE	FALSE	FALSE	ieu-a-118	0.0189	46351	0.2262	Autism Sp	TRUE	3.15E-06	0.03064	CMV IgG	TRUE	reported	igd	2	TRUE
rs7715384	T	C	T	C	-0.15364	0.0169	0.14173	NA	FALSE	FALSE	FALSE	ieu-a-118	0.0195	46351	0.3852	Autism Sp	TRUE	4.93E-06	0.03359	CMV IgG	TRUE	reported	igd	2	TRUE
rs7761068	G	A	G	A	-0.11157	0.0395	0.38685	NA	FALSE	FALSE	FALSE	ieu-a-118	0.0139	46351	0.00463	Autism Sp	TRUE	1.25E-06	0.02298	CMV IgG	TRUE	reported	igd	2	TRUE
rs10269595	G	T	G	T	0.1017	0.0091	0.45381	NA	FALSE	FALSE	FALSE	QYMDBd	0.0096	225534	0.3436	Attention	TRUE	4.80E-06	0.02221	CMV IgG	TRUE	reported	igd	2	TRUE
rs12214648	C	A	C	A	0.12335	0.0033	0.24473	NA	FALSE	FALSE	FALSE	QYMDBd	0.0112	225534	0.7683	Attention	TRUE	2.33E-06	0.02608	CMV IgG	TRUE	reported	igd	2	TRUE
rs146990284	A	G	A	G	0.41618	-0.0186	0.01683	NA	FALSE	FALSE	FALSE	QYMDBd	0.0369	225534	0.6146	Attention	TRUE	1.18E-06	0.08552	CMV IgG	TRUE	reported	igd	2	TRUE
rs2467112	T	C	T	C	-0.12442	-0.0092	0.23559	NA	FALSE	FALSE	FALSE	QYMDBd	0.0111	225534	0.4045	Attention	TRUE	4.53E-06	0.02709	CMV IgG	TRUE	reported	igd	2	TRUE
rs59559055	A	G	A	G	-0.14299	-0.0352	0.15722	NA	FALSE	FALSE	FALSE	QYMDBd	0.0128	225534	0.006	Attention	TRUE	3.15E-06	0.03064	CMV IgG	TRUE	reported	igd	2	TRUE
rs7715384	T	C	T	C	-0.15364	-0.0217	0.14173	NA	FALSE	FALSE	FALSE	QYMDBd	0.0132	225534	0.1004	Attention	TRUE	4.93E-06	0.03359	CMV IgG	TRUE	reported	igd	2	TRUE
rs7761068	G	A	G	A	-0.11157	0.0108	0.38685	NA	FALSE	FALSE	FALSE	QYMDBd	0.0094	225534	0.2504	Attention	TRUE	1.25E-06	0.02298	CMV IgG	TRUE	reported	igd	2	TRUE
rs10269595	G	T	G	T	0.1017	-0.001	0.45381	NA	FALSE	FALSE	FALSE	tc0dZp	0.026	14307	0.969	Tourette s	TRUE	4.80E-06	0.02221	CMV IgG	TRUE	reported	igd	2	TRUE
rs12214648	C	A	C	A	0.12335	-0.0399	0.24473	NA	FALSE	FALSE	FALSE	tc0dZp	0.0305	14307	0.1906	Tourette s	TRUE	2.33E-06	0.02608	CMV IgG	TRUE	reported	igd	2	TRUE
rs146990284	A	G	A	G	0.41618	-0.0548	0.01683	NA	FALSE	FALSE	FALSE	tc0dZp	0.1273	14307	0.6672	Tourette s	TRUE	1.18E-06	0.08552	CMV IgG	TRUE	reported	igd	2	TRUE
rs2467112	T	C	T	C	-0.12442	-0.0563	0.23559	NA	FALSE	FALSE	FALSE	tc0dZp	0.0319	14307	0.07771	Tourette s	TRUE	4.53E-06	0.02709	CMV IgG	TRUE	reported	igd	2	TRUE
rs59559055	A	G	A	G	-0.14299	-0.0132	0.15722	NA	FALSE	FALSE	FALSE	tc0dZp	0.0366	14307	0.7186	Tourette s	TRUE	3.15E-06	0.03064	CMV IgG	TRUE	reported	igd	2	TRUE
rs7715384	T	C	T	C	-0.15364	-0.0901	0.14173	NA	FALSE	FALSE	FALSE	tc0dZp	0.0374	14307	0.01603	Tourette s	TRUE	4.93E-06	0.03359	CMV IgG	TRUE	reported	igd	2	TRUE
rs118172547	G	A	G	A	1.04225	0.0217	0.02669	NA	FALSE	FALSE	FALSE	ieu-a-118	0.0777	46351	0.7798	Autism Sp	TRUE	4.42E-06	0.22704	CMV infec	TRUE	reported	mxSHnU	2	TRUE
rs12438477	A	C	A	C	-0.29335	0.0048	0.35703	NA	FALSE	FALSE	FALSE	ieu-a-118	0.0143	46351	0.7367	Autism Sp	TRUE	4.32E-06	0.06383	CMV infec	TRUE	reported	mxSHnU	2	TRUE
rs13156302	T	C	T	C	0.39166	-0.0093	0.20362	NA	FALSE	FALSE	FALSE	ieu-a-118	0.0177	46351	0.5995	Autism Sp	TRUE	1.21E-06	0.08068	CMV infec	TRUE	reported	mxSHnU	2	TRUE
rs61825717	G	A	G	A	1.05566	0.0771	0.03388	NA	FALSE	FALSE	FALSE	ieu-a-118	0.046	46351	0.0942	Autism Sp	TRUE	1.94E-07	0.20282	CMV infec	TRUE	reported	mxSHnU	2	TRUE
rs118172547	G	A	G	A	1.04225	0.0156	0.02669	NA	FALSE	FALSE	FALSE	jtl9Lb	0.0474	225534	0.7427	Attention	TRUE	4.42E-06	0.22704	CMV infec	TRUE	reported	mxSHnU	2	TRUE
rs12438477	A	C	A	C	-0.29335	-0.0015	0.35703	NA	FALSE	FALSE	FALSE	jtl9Lb	0.011	225534	0.89	Attention	TRUE	4.32E-06	0.06383	CMV infec	TRUE	reported	mxSHnU	2	TRUE
rs13156302	T	C	T	C	0.39166	-0.0068	0.20362	NA	FALSE	FALSE	FALSE	jtl9Lb	0.012	225534	0.5692	Attention	TRUE	1.21E-06	0.08068	CMV infec	TRUE	reported	mxSHnU	2	TRUE
rs61825717	G	A	G	A	1.05566	0.0061	0.03388	NA	FALSE	FALSE	FALSE	jtl9Lb	0.0306	225534	0.8417	Attention	TRUE	1.94E-07	0.20282	CMV infec	TRUE	reported	mxSHnU	2	TRUE
rs117479615	G	A	G	A	1.03288	0.1536	0.02965	NA	FALSE	FALSE	FALSE	cSC9qG	0.1503	14307	0.307	Tourette s	TRUE	1.88E-06	0.21672	CMV infec	TRUE	reported	mxSHnU	2	TRUE
rs12438477	A	C	A	C	-0.29335	-0.0013	0.35703	NA	FALSE	FALSE	FALSE	cSC9qG	0.0267	14307	0.9623	Tourette s	TRUE	4.32E-06	0.06383	CMV infec	TRUE	reported	mxSHnU	2	TRUE
rs13156302	T	C	T	C	0.39166	-0.0303	0.20362	NA	FALSE	FALSE	FALSE	cSC9qG	0.0327	14307	0.3541	Tourette s	TRUE	1.21E-06	0.08068	CMV infec	TRUE	reported	mxSHnU	2	TRUE
rs61825717	G	A	G	A	1.05566	0.0002	0.03388	NA	FALSE	FALSE	FALSE	cSC9qG	0.0995	14307	0.9985	Tourette s	TRUE	1.94E-07	0.20282	CMV infec	TRUE	reported	mxSHnU	2	TRUE

Table S3. Details of the genetic variants with potential pleiotropy among instrumental variables by GWAS catalog searching.

riskAllele	pValue	pValueAn	riskFreque	orValue	beta	ci	mappedG	traitName	efoTraits	bgTraits	accessionI	locations	pubmedId	author
rs2858331-G	3.00E-07	-	NR	1.16252	-	NR	MTCO3P1	Non-Hodgkin's lymphoma	non-Hodg	-	GCST9001	6:3271350	32887889	Rashkin SR
rs2858331-G	1.00E-08	-	NR	-	0.04 unit decrease	[NR]	MTCO3P1	IgE levels	serum IgE	-	GCST0013	6:3271350	22075330	Granada M
rs2858331-G	6.00E-06	-	NR	-	-	-	MTCO3P1	IgE levels	serum IgE	-	GCST0017	6:3271350	23146381	Levin AM
rs2858331-G	4.00E-08	-	0.33	3.82	-	[2.93-4.97]	MTCO3P1	Immunoglobulin A vasculitis	Henoch-S	-	GCST0115	6:3271350	33591409	Koskela M
rs2858331-G	3.00E-10	-	NR	-	-	-	MTCO3P1	Alzheimer's disease or gastro	Alzheimer	-	GCST9013	6:3271350	35851147	Adewuyi EO
rs2858331-G	8.00E-26	-	0.366737	1.19	-	[1.15-1.22]	MTCO3P1	Anti-SARS-CoV-2 spike prote	anti-SARS	-	GCST9029	6:3271350	38181733	Bian S
rs78742138-T	5.00E-24	(SVEP1, 11	0.963906	-	0.61348087 unit de	[0.5-0.73]	SVEP1	Blood protein levels	blood pro	-	GCST0065	9:1104984	30072576	Emilsson V
rs78742138-T	8.00E-28	(SVEP1, 11	0.963906	-	0.6651358 unit dec	[0.55-0.78]	SVEP1	Blood protein levels	blood pro	-	GCST0065	9:1104984	30072576	Emilsson V
rs78742138-C	2.00E-14	-	0.03952	-	0.361542 unit incre	[0.27-0.45]	SVEP1	Serum levels of protein BID	blood pro	-	GCST9008	9:1104984	35078996	Gudjonsson A
rs78742138-C	7.00E-48	-	0.03952	-	0.643837 unit incre	[0.56-0.73]	SVEP1	Serum levels of protein SVEP1	blood pro	-	GCST9008	9:1104984	35078996	Gudjonsson A
rs78742138-C	2.00E-56	-	0.03952	-	0.701953 unit incre	[0.62-0.79]	SVEP1	Serum levels of protein SVEP1	blood pro	-	GCST9008	9:1104984	35078996	Gudjonsson A
rs3130169-?	2.00E-08	-	NR	-	-	-	HLA-DPB1	Asthma (childhood onset)	childhood	-	GCST0098	6:3308032	31669095	Zhu Z
rs78742138-T	5.00E-24	(SVEP1, 11	0.963906	-	0.61348087 unit de	[0.5-0.73]	SVEP1	Blood protein levels	blood pro	-	GCST0065	9:1104984	30072576	Emilsson V
rs78742138-T	8.00E-28	(SVEP1, 11	0.963906	-	0.6651358 unit dec	[0.55-0.78]	SVEP1	Blood protein levels	blood pro	-	GCST0065	9:1104984	30072576	Emilsson V
rs78742138-C	2.00E-14	-	0.03952	-	0.361542 unit incre	[0.27-0.45]	SVEP1	Serum levels of protein BID	blood pro	-	GCST9008	9:1104984	35078996	Gudjonsson A
rs78742138-C	7.00E-48	-	0.03952	-	0.643837 unit incre	[0.56-0.73]	SVEP1	Serum levels of protein SVEP1	blood pro	-	GCST9008	9:1104984	35078996	Gudjonsson A
rs78742138-C	2.00E-56	-	0.03952	-	0.701953 unit incre	[0.62-0.79]	SVEP1	Serum levels of protein SVEP1	blood pro	-	GCST9008	9:1104984	35078996	Gudjonsson A
