# Supplementary material for: Association between human herpesviruses infections and childhood neurodevelopmental disorders: insights from two-sample mendelian randomization analyses and systematic review with meta-analysis
Source: Ital J Pediatr. 2024 Nov 20;50:248. doi: 10.1186/s13052-024-01820-9 (PMC11580506; doi:10.1186/s13052-024-01820-9)
Supplement: Supplementary file 2 [file 13052_2024_1820_MOESM2_ESM.pdf]

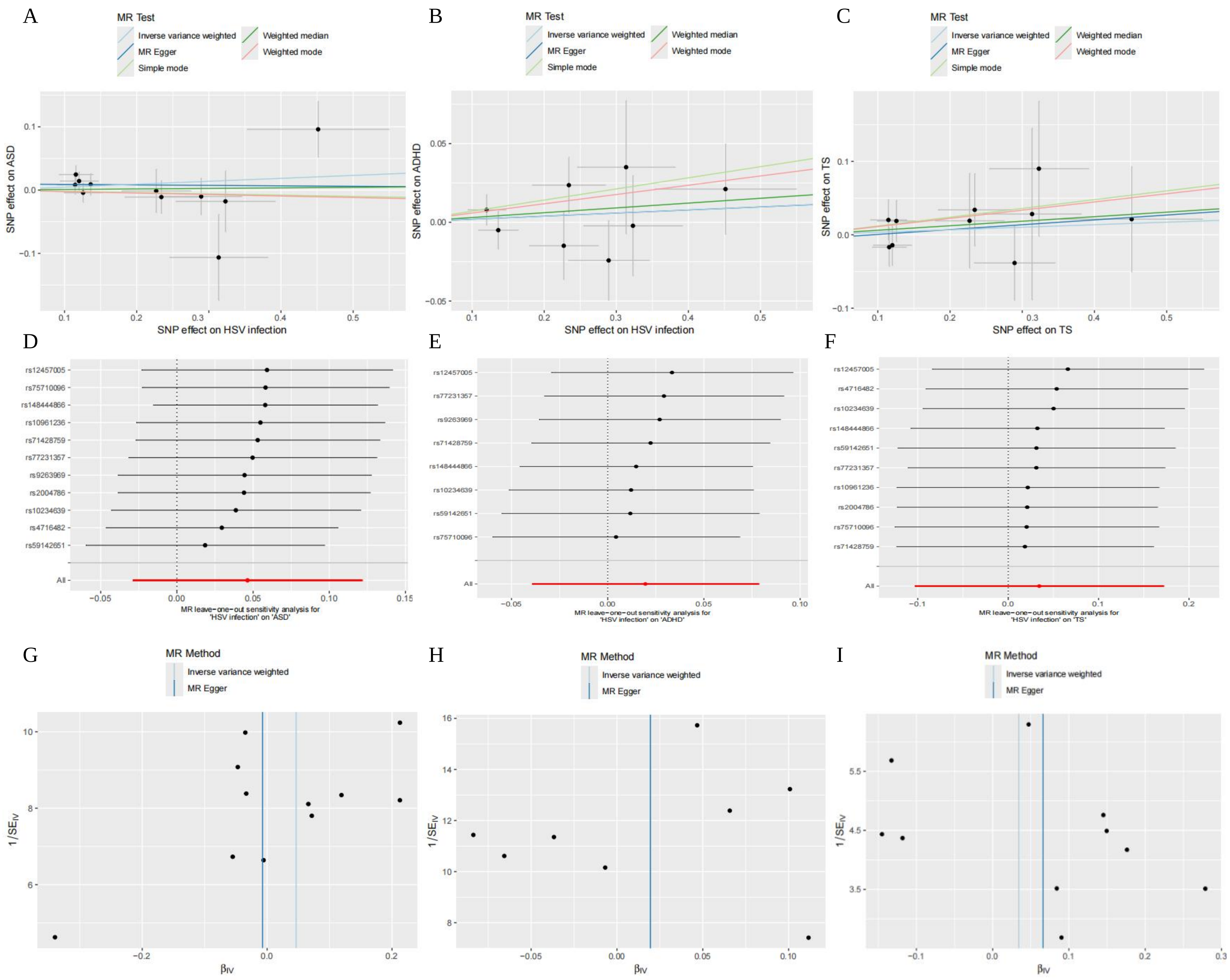

Figure S1. Scatter plots, leave-one-out analysis, and funnel plots of genetic associations between HSV infections and ASD, ADHD, TS using different MR methods. A-C: Scatter plots of IVs on ASD, ADHD, and TS; D-F: Leave-one-out analysis of IVs on ASD, ADHD, and TS; G-I: Funnel plots of IVs on ASD, ADHD, and TS. HSV: herpes simplex virus; ASD: autism spectrum disorders; ADHD: attention deficit hyperactivity disorder; TS: tourette syndrome.

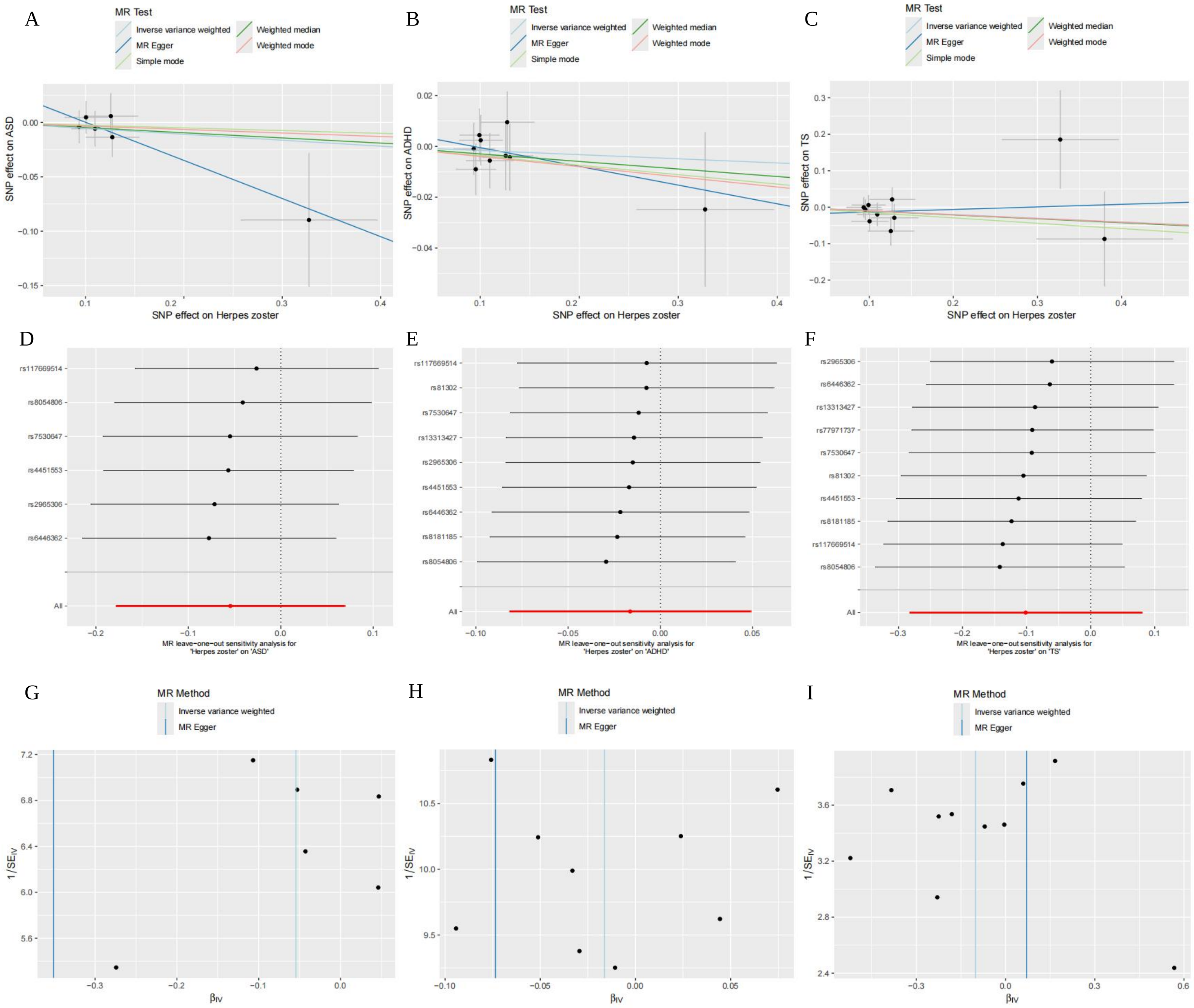

Figure S2. Scatter plots, leave-one-out analysis, and funnel plots of genetic associations between Herpes zoster and ASD, ADHD, TS using different MR methods. A-C: Scatter plots of IVs on ASD, ADHD, and TS; D-F: Leave-one-out analysis of IVs on ASD, ADHD, and TS; G-I: Funnel plots of IVs on ASD, ADHD, and TS. ASD: autism spectrum disorders; ADHD: attention deficit hyperactivity disorder; TS: tourette syndrome.

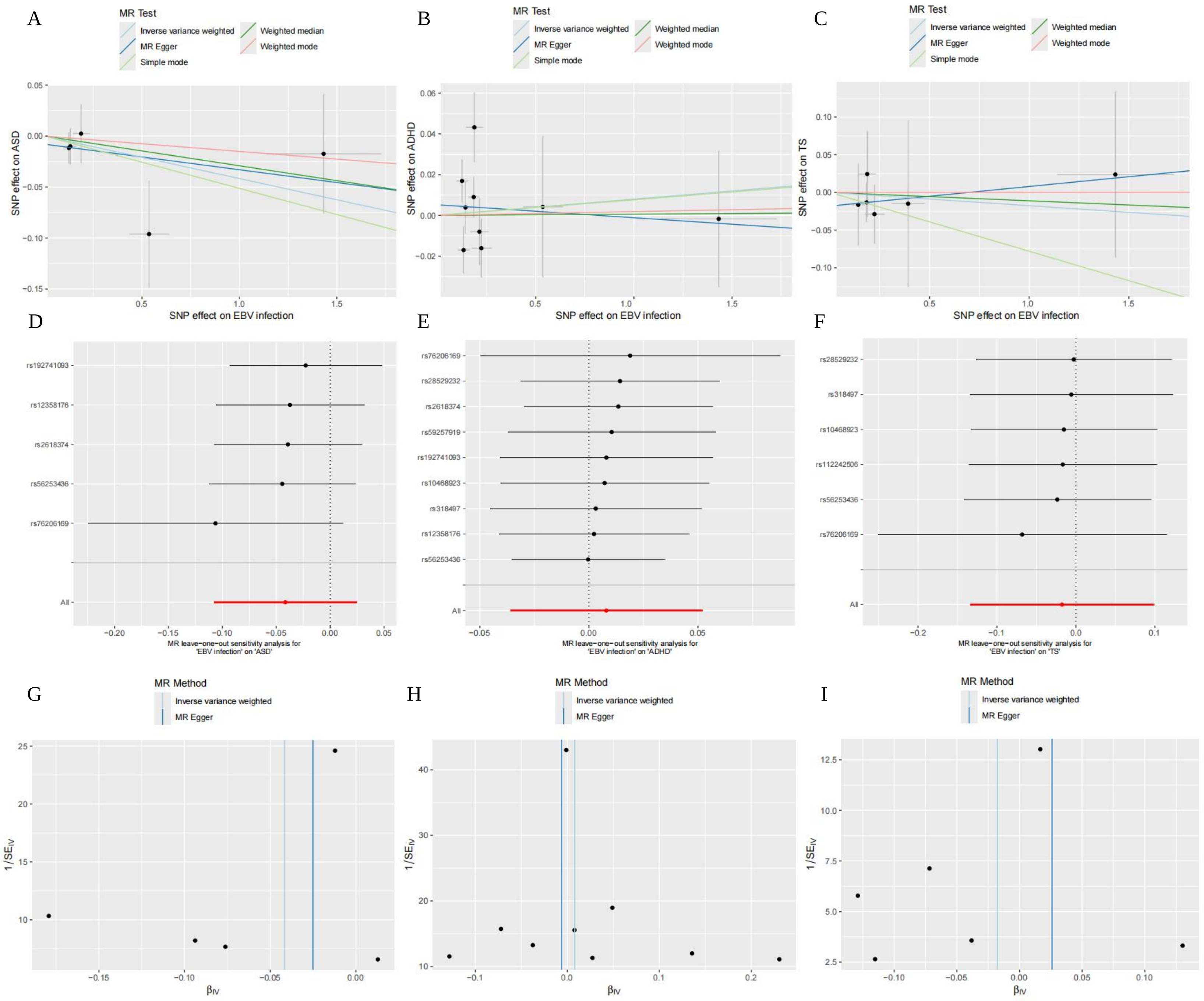

Figure S3. Scatter plots, leave-one-out analysis, and funnel plots of genetic associations between EBV infections and ASD, ADHD, TS using different MR methods. A-C: Scatter plots of IVs on ASD, ADHD, and TS; D-F: Leave-one-out analysis of IVs on ASD, ADHD, and TS; G-I: Funnel plots of IVs on ASD, ADHD, and TS. EBV: Epstein-Barr virus; ASD: autism spectrum disorders; ADHD: attention deficit hyperactivity disorder; TS: tourette syndrome.

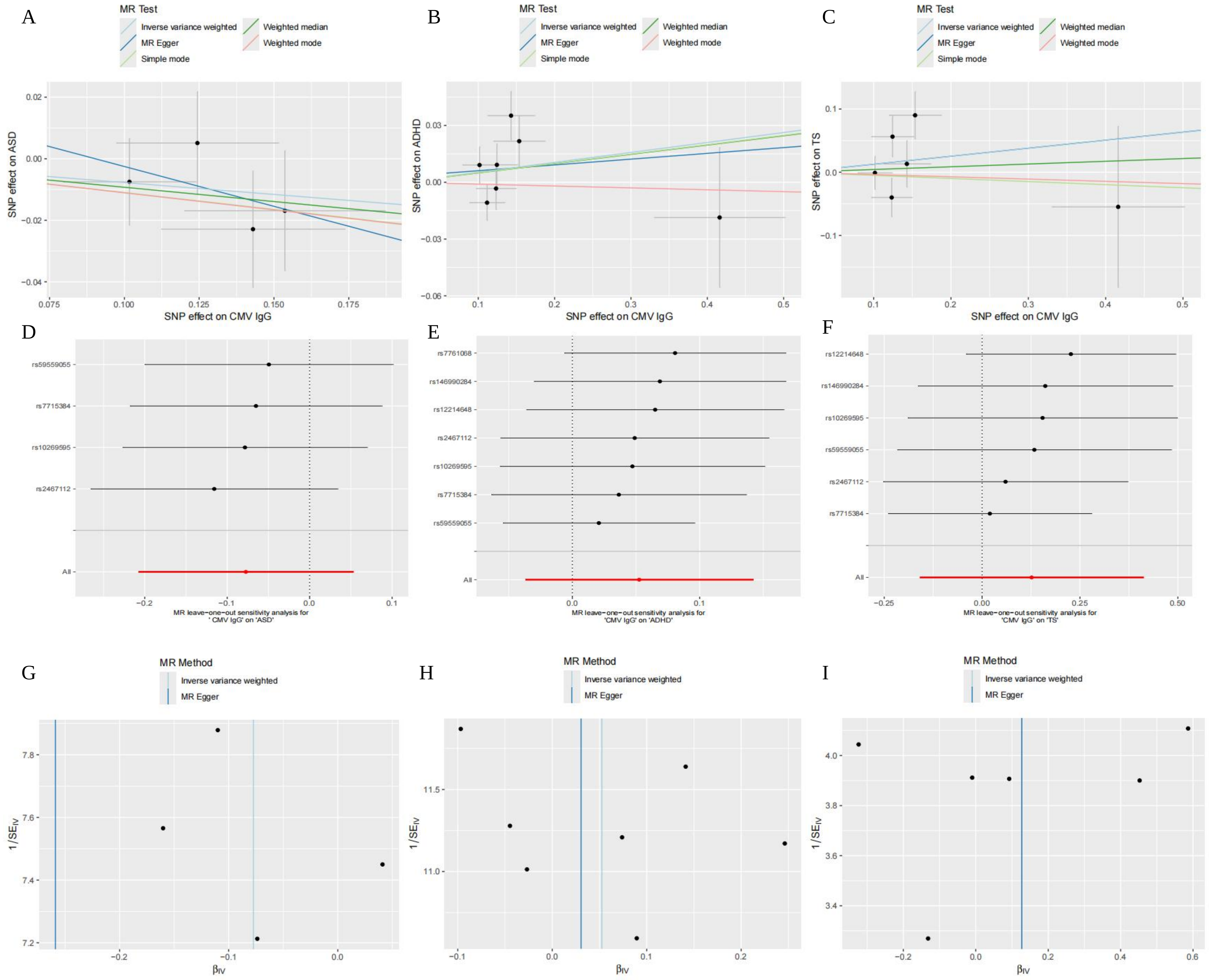

Figure S4. Scatter plots, leave-one-out analysis, and funnel plots of genetic associations between CMV IgG and ASD, ADHD, TS using different MR methods. A-C: Scatter plots of IVs on ASD, ADHD, and TS; D-F: Leave-one-out analysis of IVs on ASD, ADHD, and TS; G-I: Funnel plots of IVs on ASD, ADHD, and TS. CMV: cytomegalovirus; ASD: autism spectrum disorders; ADHD: attention deficit hyperactivity disorder; TS: tourette syndrome.

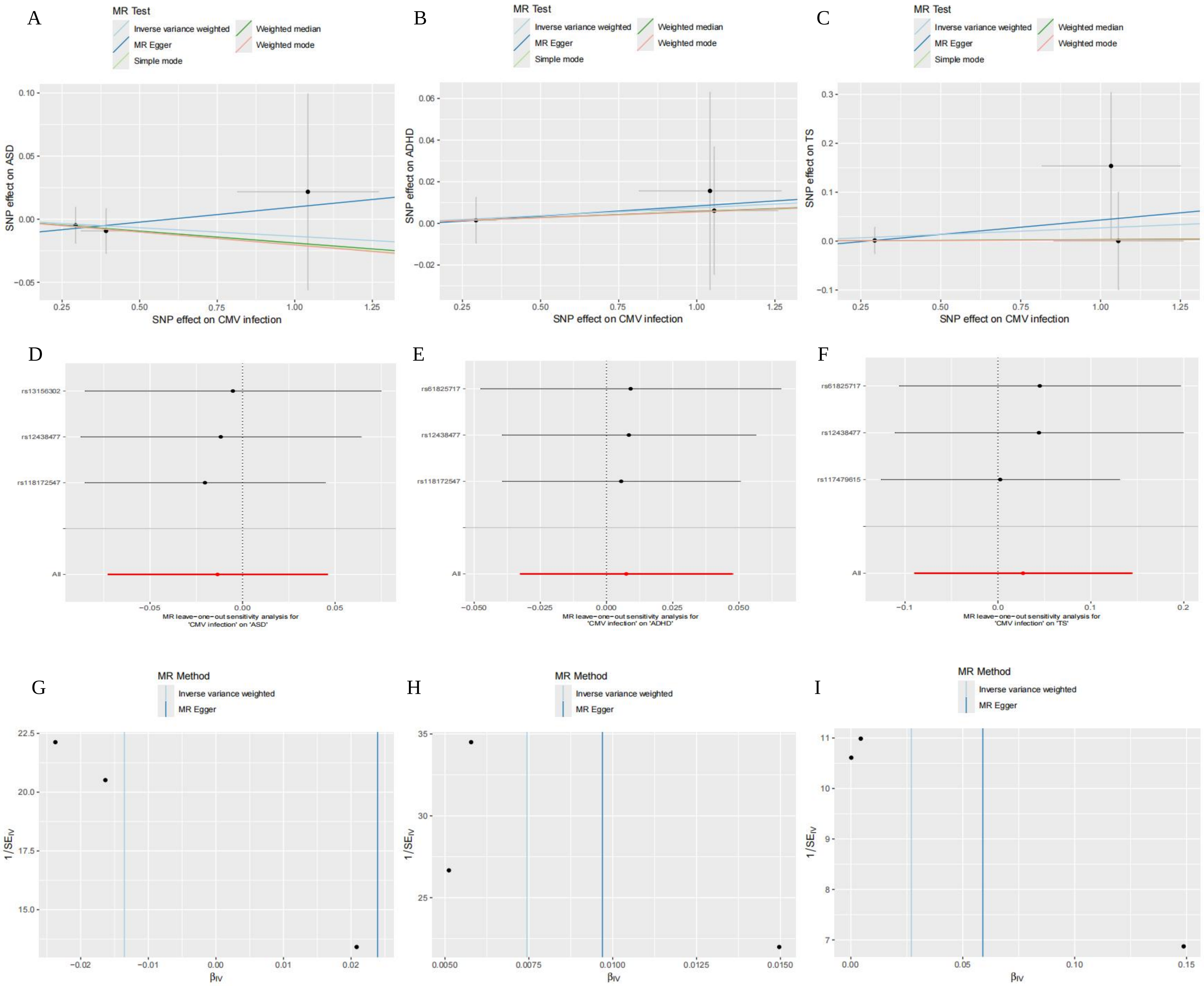

Figure S5. Scatter plots, leave-one-out analysis, and funnel plots of genetic associations between CMV infections and ASD, ADHD, TS using different MR methods.
A-C: Scatter plots of IVs on ASD, ADHD, and TS; D-F: Leave-one-out analysis of IVs on ASD, ADHD, and TS; G-I: Funnel plots of IVs on ASD, ADHD, and TS. CMV: cytomegalovirus; ASD: autism spectrum disorders; ADHD: attention deficit hyperactivity disorder; TS: tourette syndrome.
