# Supplementary material for: Association between human herpesviruses infections and childhood neurodevelopmental disorders: insights from two-sample mendelian randomization analyses and systematic review with meta-analysis
Source: Ital J Pediatr. 2024 Nov 20;50:248. doi: 10.1186/s13052-024-01820-9 (PMC11580506; doi:10.1186/s13052-024-01820-9)
Supplement: Supplementary file 3 [file 13052_2024_1820_MOESM3_ESM.pdf]

Figure S1. Meta-analysis for the association between HSV infection and ASD

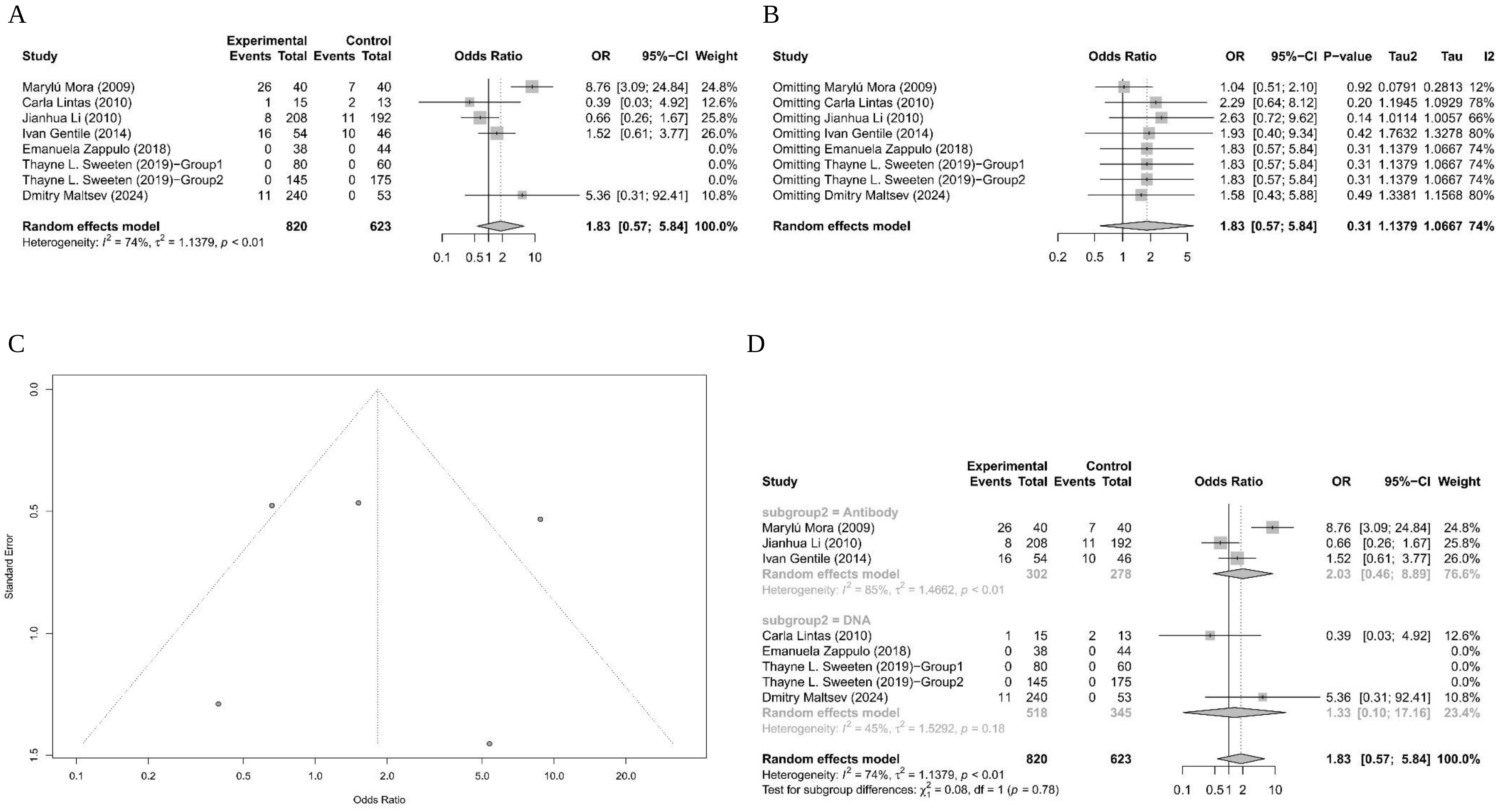

A. forest plot of meta-analysis; B. sensitivity test (leave-one-out plot); C. funnel plot; D. subgroup analysis of detection indicator.

Figure S2. Meta-analysis for the association between EBV infection and ASD

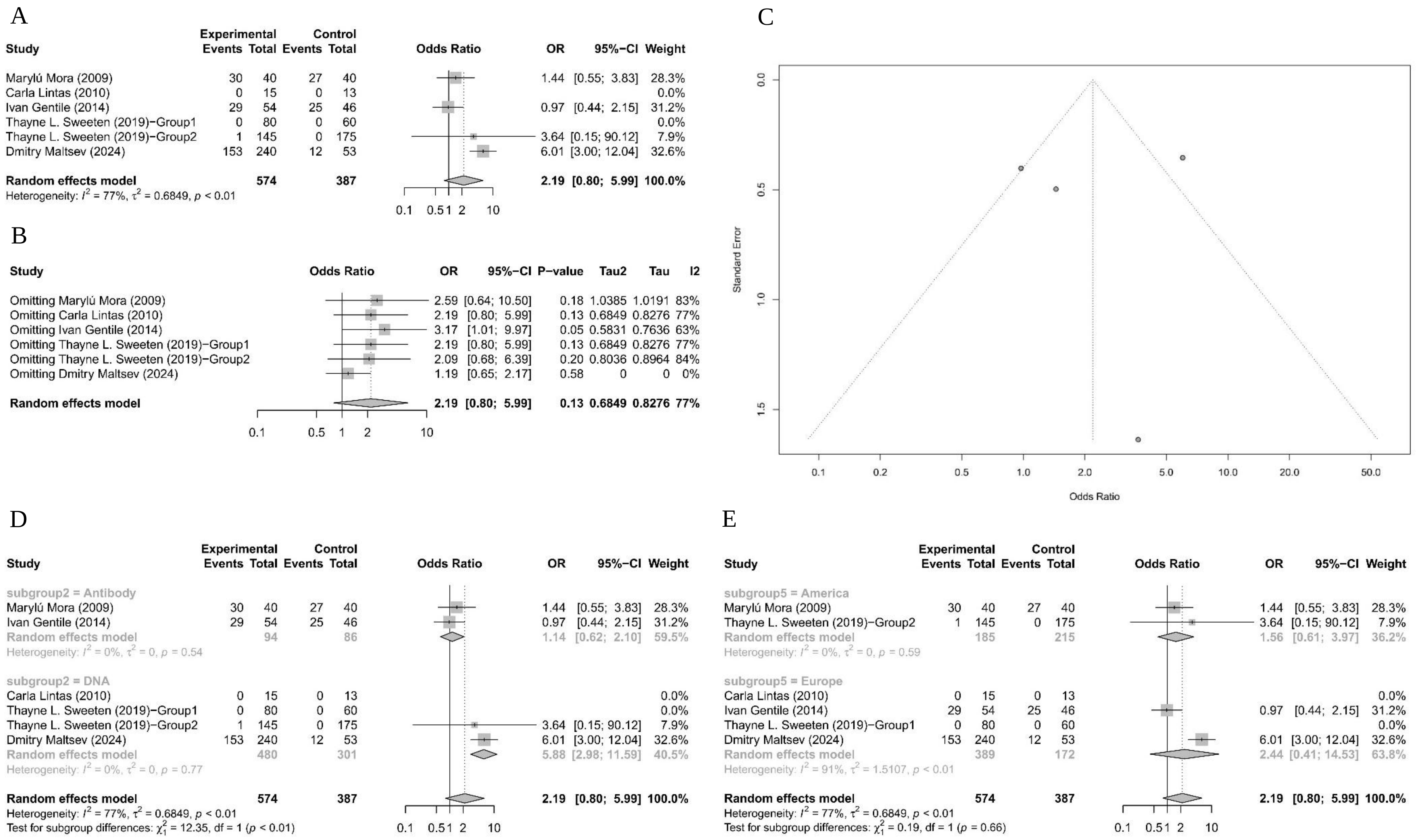

A. forest plot of meta-analysis; B. sensitivity test (leave-one-out plot); C. funnel plot; D. subgroup analysis of detection indicator; E. subgroup analysis of region

Figure S3. Meta-analysis for the association between CMV infection and ASD

A

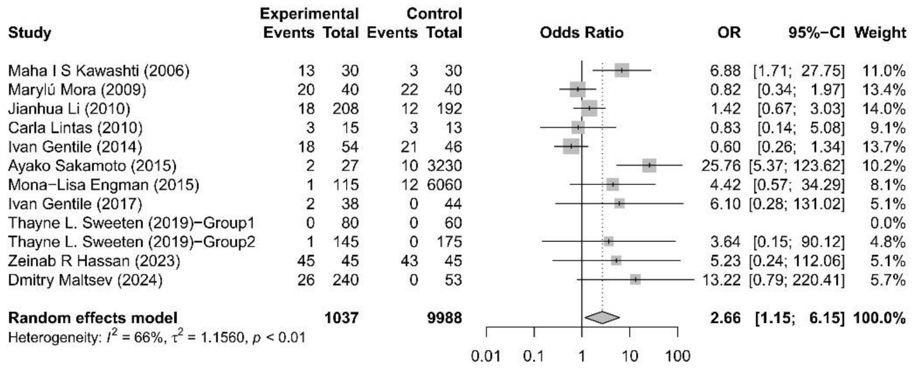

B

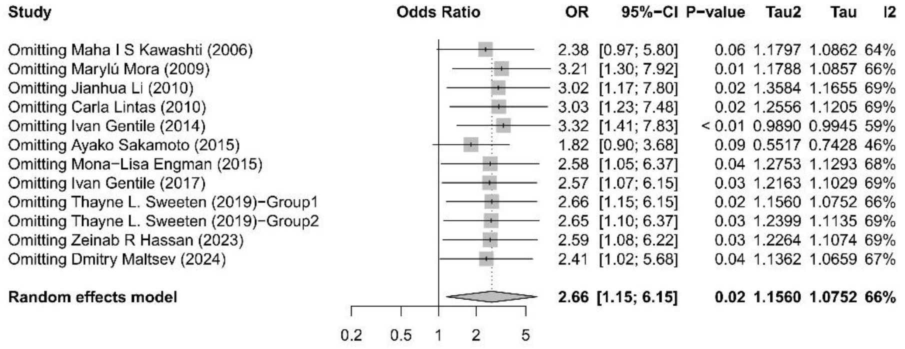

C

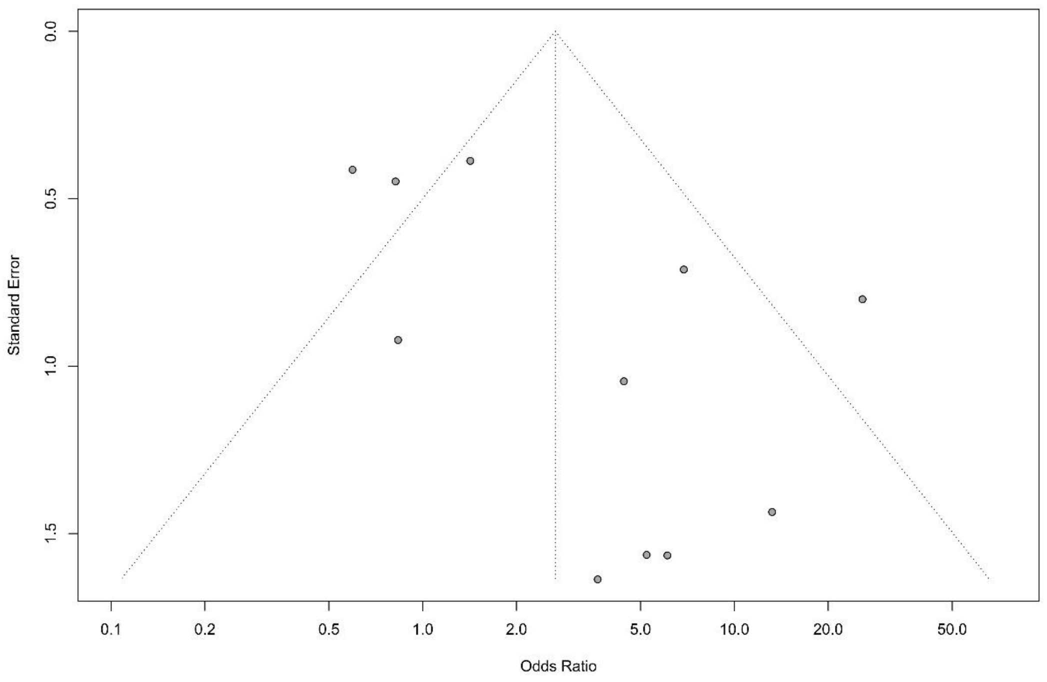

A. forest plot of meta-analysis; B. sensitivity test (leave-one-out plot); C. funnel plot

Figure S4. Subgroup analysis of the association between CMV infection and ASD (1)

A

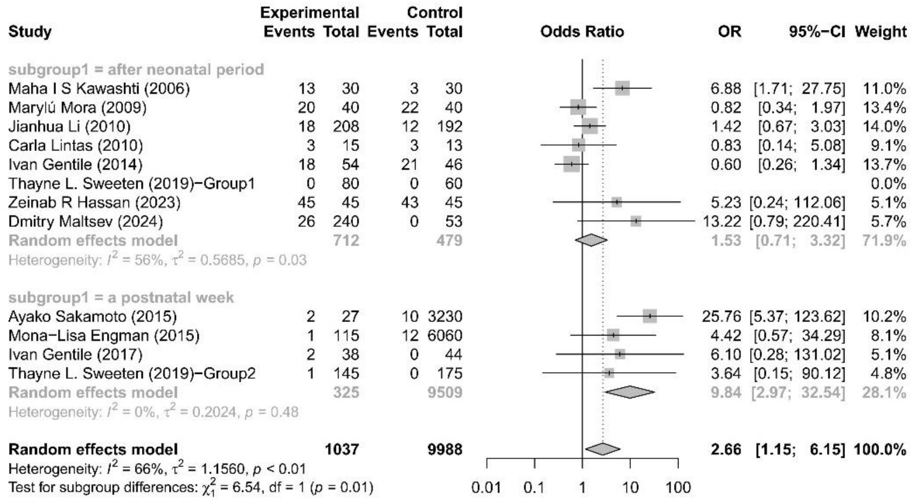

B

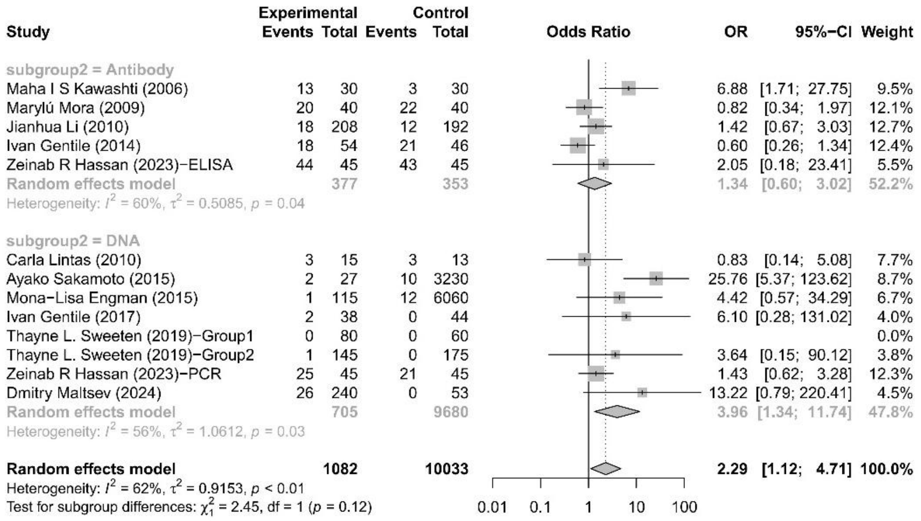

A. subgroup analysis of sample collection time; B. subgroup analysis of detection indicator

Figure S5. Subgroup analysis of the association between CMV infection and ASD (2)

A

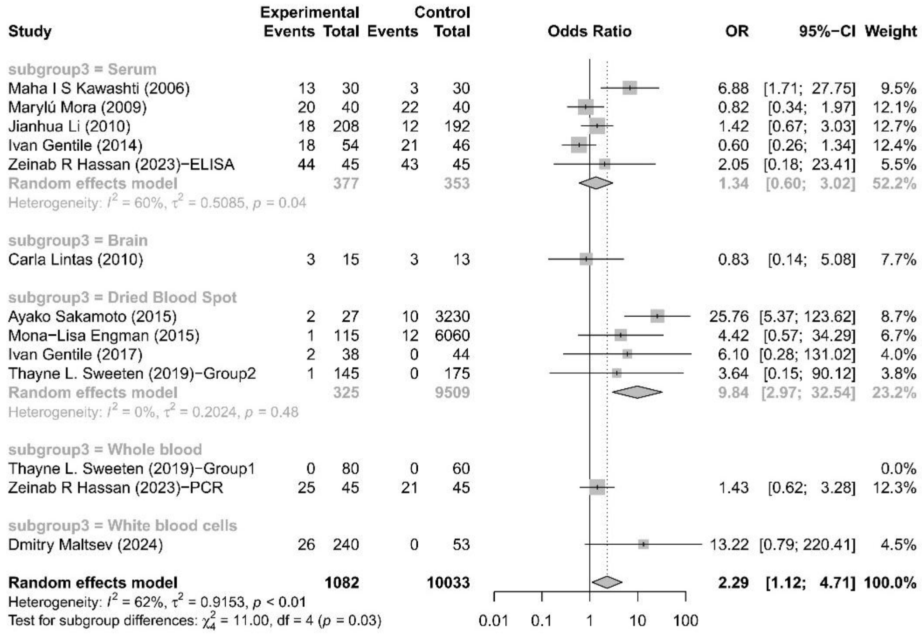

B

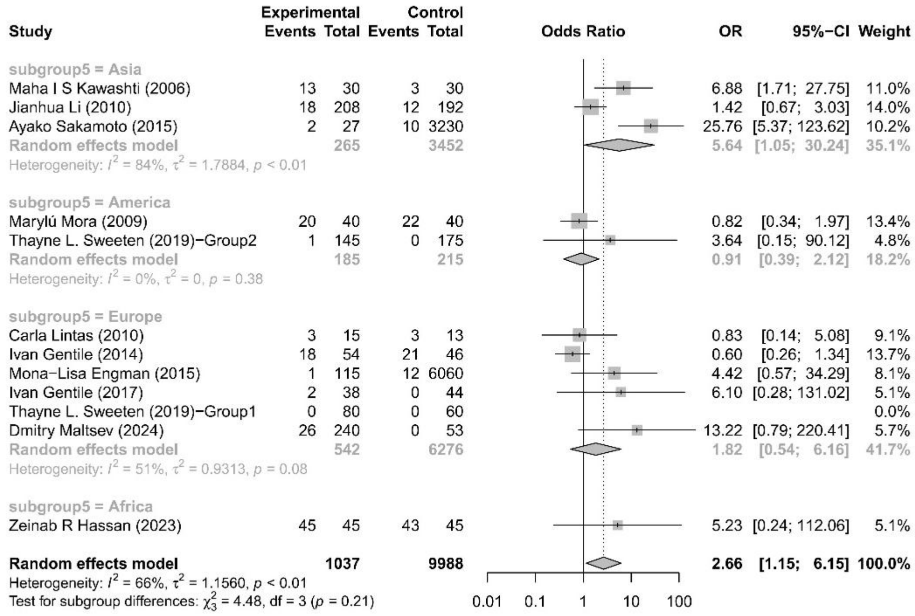

A. subgroup analysis of sample source; B. subgroup analysis of region

Figure S6. Meta-analysis for the association between HHV-6 infection and ASD

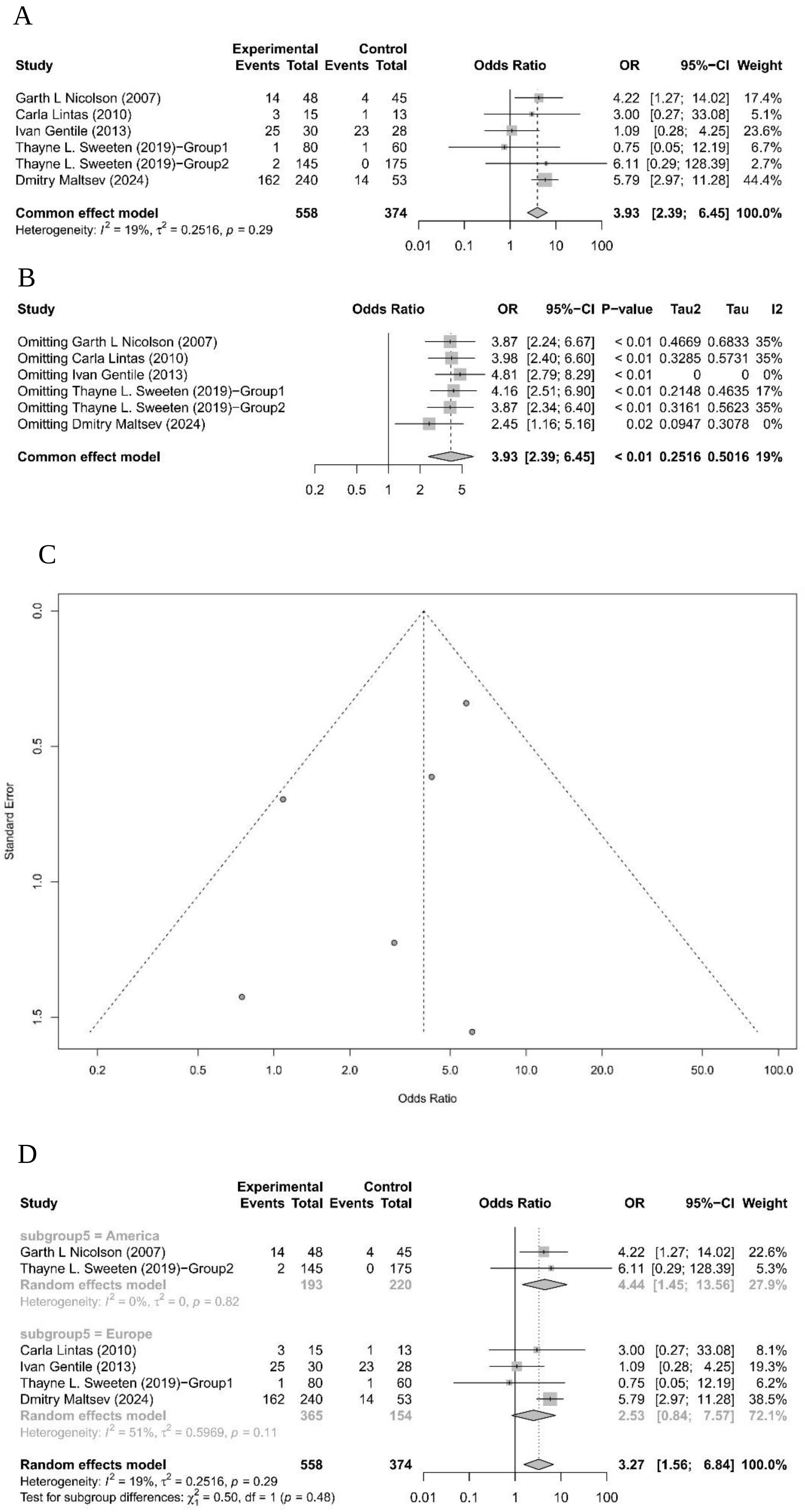

A. forest plot of meta-analysis; B. sensitivity test (leave-one-out plot); C. funnel plot; D. subgroup analysis of region

Figure S7. Meta-analysis for the association between HHV-7 infection and ASD

A

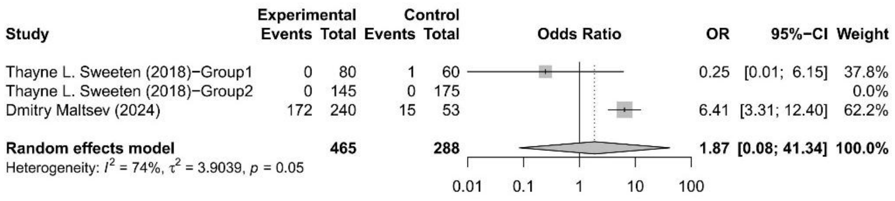

B

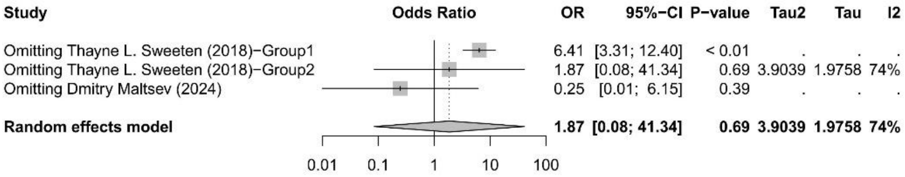

C

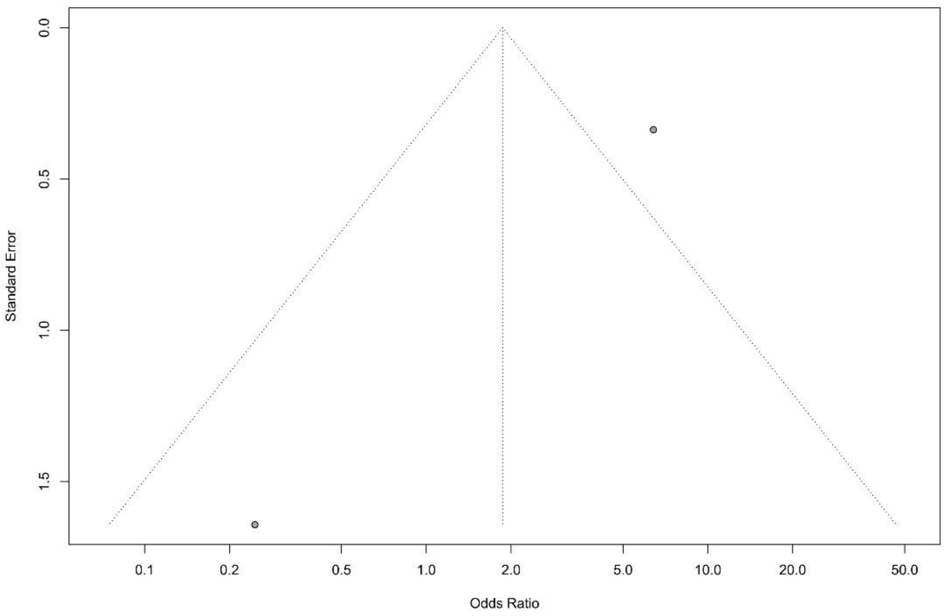

A. forest plot of meta-analysis; B. sensitivity test (leave-one-out plot); C. funnel plot

Figure S8. Meta-analysis for the association between CMV infection and TD

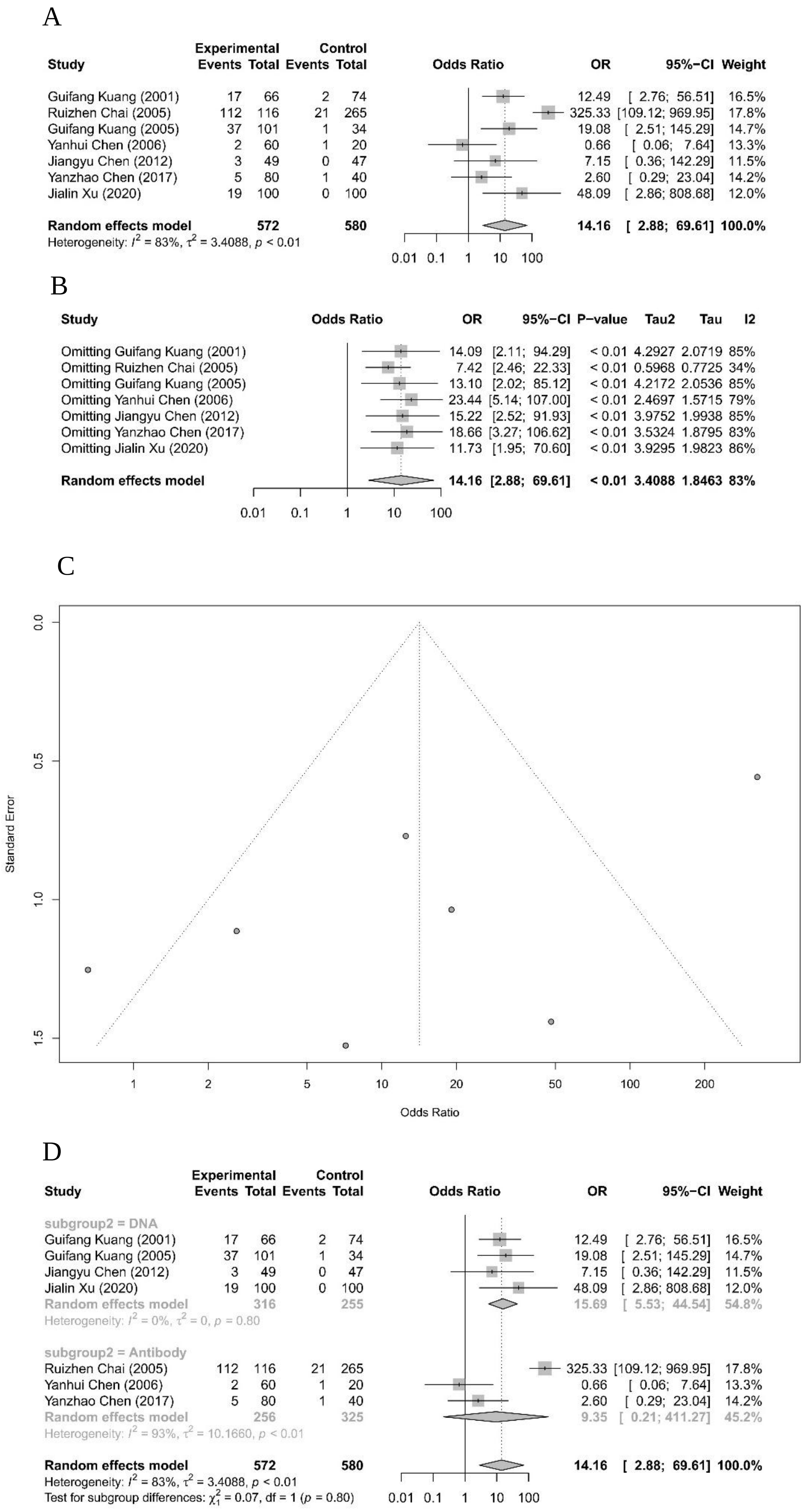

A. forest plot of meta-analysis; B. sensitivity test (leave-one-out plot); C. funnel plot; D. subgroup analysis of detection indicator.

Figure S9. Meta-analysis for the association between EBV infection and TD

A

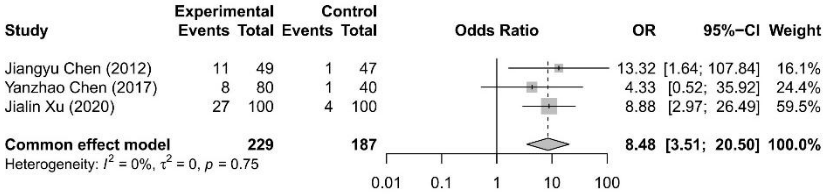

B

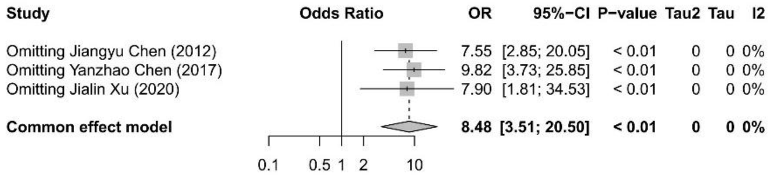

C

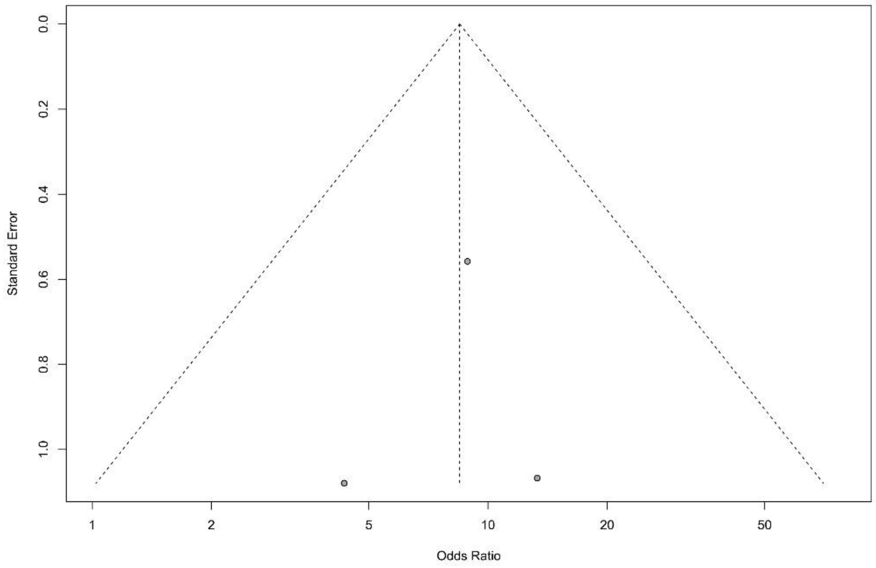

A. forest plot of meta-analysis; B. sensitivity test (leave-one-out plot); C. funnel plot

Figure S10. Meta-analysis for the association between CMV infection and ADHD

A

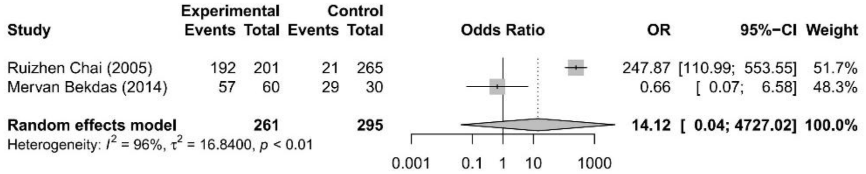

B

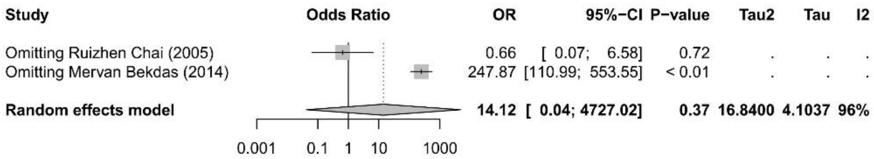

C

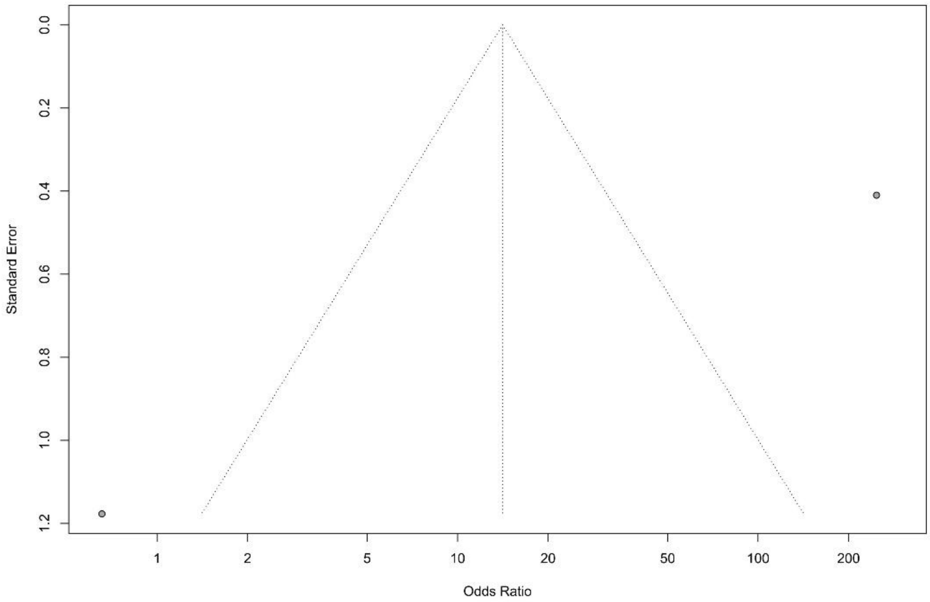

A. forest plot of meta-analysis; B. sensitivity test (leave-one-out plot); C. funnel plot

Figure S11. Meta-analysis for the association between CMV infection and ADHD (retrospective cohort study)

A

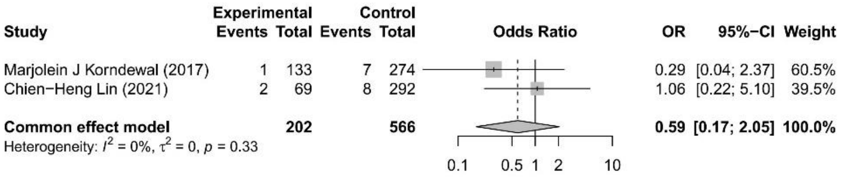

B

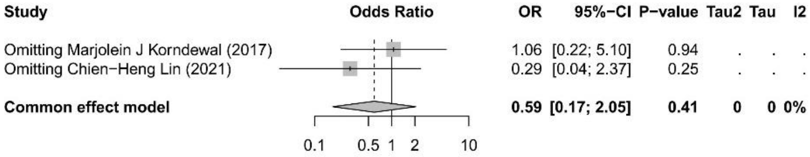

C

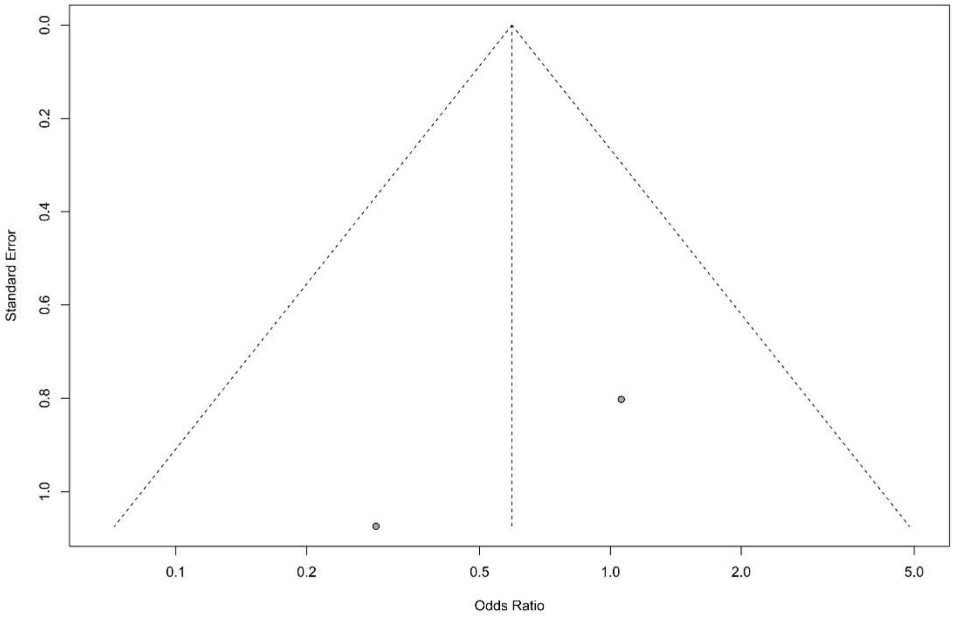

A. forest plot of meta-analysis; B. sensitivity test (leave-one-out plot); C. funnel plot

Figure S12. Meta-analysis for the association between CMV infection and ASD (retrospective cohort study)

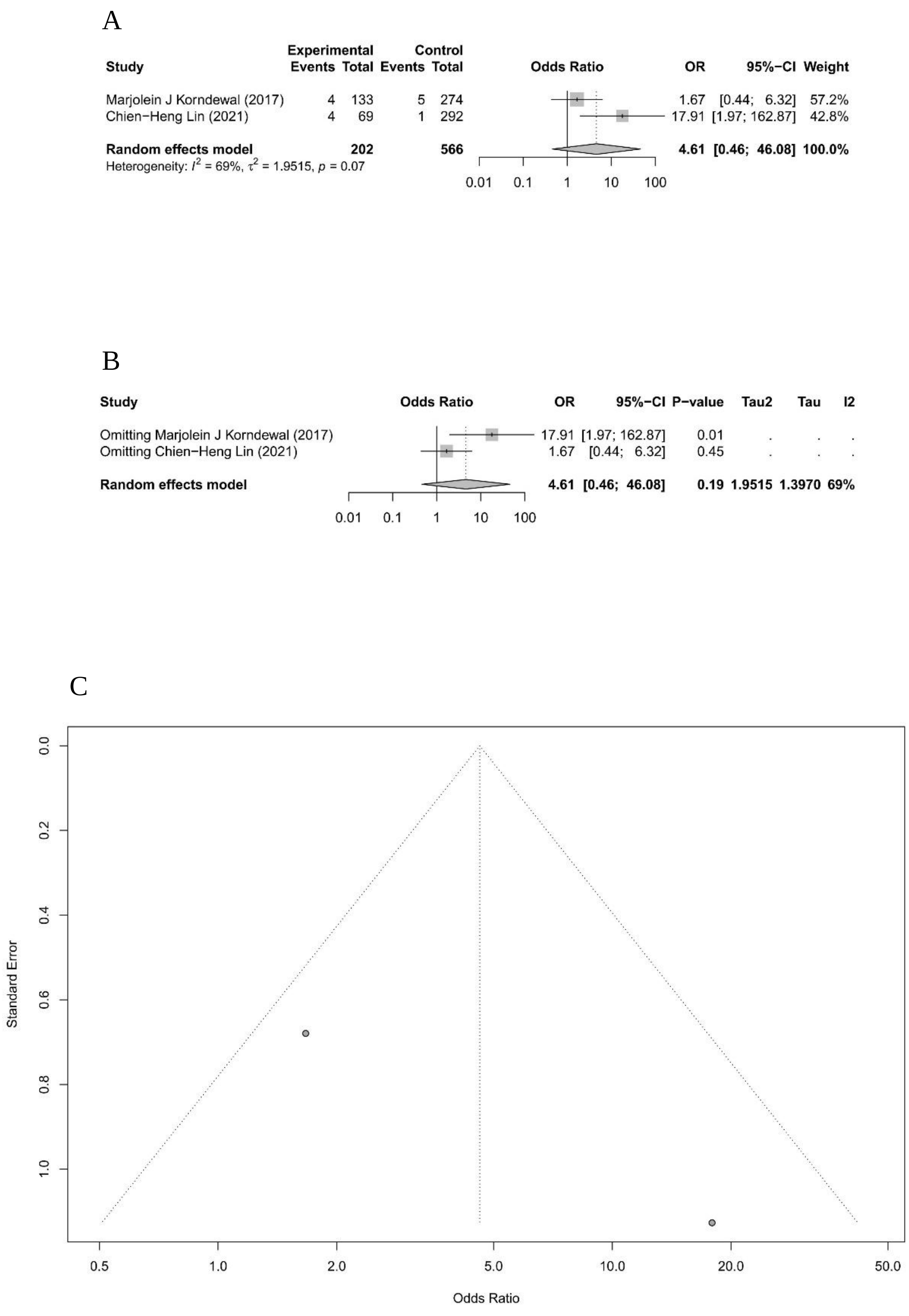

A. forest plot of meta-analysis; B. sensitivity test (leave-one-out plot); C. funnel plot
