# Supplementary material for: Association between human herpesviruses infections and childhood neurodevelopmental disorders: insights from two-sample mendelian randomization analyses and systematic review with meta-analysis
Source: Ital J Pediatr. 2024 Nov 20;50:248. doi: 10.1186/s13052-024-01820-9 (PMC11580506; doi:10.1186/s13052-024-01820-9)
Supplement: Supplementary file 4 [file 13052_2024_1820_MOESM4_ESM.pdf]

PRISMA 2020 Checklist

Section and Topic	Item #	Checklist item	Location where item is reported
**TITLE**			
Title	1	Identify the report as a systematic review.	Line 1-3
**ABSTRACT**			
Abstract	2	See the PRISMA 2020 for Abstracts checklist.	Line 20-40
**INTRODUCTION**			
Rationale	3	Describe the rationale for the review in the context of existing knowledge.	Line 46-83
Objectives	4	Provide an explicit statement of the objective(s) or question(s) the review addresses.	Line 94-97
**METHODS**			
Eligibility criteria	5	Specify the inclusion and exclusion criteria for the review and how studies were grouped for the syntheses.	Line 174-182
Information sources	6	Specify all databases, registers, websites, organisations, reference lists and other sources searched or consulted to identify studies. Specify the date when each source was last searched or consulted.	Line 164-172
Search strategy	7	Present the full search strategies for all databases, registers and websites, including any filters and limits used.	Additional file 4
Selection process	8	Specify the methods used to decide whether a study met the inclusion criteria of the review, including how many reviewers screened each record and each report retrieved, whether they worked independently, and if applicable, details of automation tools used in the process.	Line 184-187<br>Additional file 3: figure S1
Data collection process	9	Specify the methods used to collect data from reports, including how many reviewers collected data from each report, whether they worked independently, any processes for obtaining or confirming data from study investigators, and if applicable, details of automation tools used in the process.	Line 195-199
Data items	10a	List and define all outcomes for which data were sought. Specify whether all results that were compatible with each outcome domain in each study were sought (e.g. for all measures, time points, analyses), and if not, the methods used to decide which results to collect.	Line 195-199
	10b	List and define all other variables for which data were sought (e.g. participant and intervention characteristics, funding sources). Describe any assumptions made about any missing or unclear information.	Line 195-199
Study risk of bias assessment	11	Specify the methods used to assess risk of bias in the included studies, including details of the tool(s) used, how many reviewers assessed each study and whether they worked independently, and if applicable, details of automation tools used in the process.	Line 188-193
Effect measures	12	Specify for each outcome the effect measure(s) (e.g. risk ratio, mean difference) used in the synthesis or presentation of results.	Line 212-214
Synthesis methods	13a	Describe the processes used to decide which studies were eligible for each synthesis (e.g. tabulating the study intervention characteristics and comparing against the planned groups for each synthesis (item #5)).	Line 184-187
	13b	Describe any methods required to prepare the data for presentation or synthesis, such as handling of missing summary statistics, or data conversions.	Line 207-218
	13c	Describe any methods used to tabulate or visually display results of individual studies and syntheses.	Line 207-218
	13d	Describe any methods used to synthesize results and provide a rationale for the choice(s). If meta-analysis was performed, describe the model(s), method(s) to identify the presence and extent of statistical heterogeneity, and software package(s) used.	Line 207-218
	13e	Describe any methods used to explore possible causes of heterogeneity among study results (e.g. subgroup analysis, meta-regression).	Line 207-218
	13f	Describe any sensitivity analyses conducted to assess robustness of the synthesized results.	Line 207-218
Reporting bias assessment	14	Describe any methods used to assess risk of bias due to missing results in a synthesis (arising from reporting biases).	Line 188-193 and Addition file 4
Certainty assessment	15	Describe any methods used to assess certainty (or confidence) in the body of evidence for an outcome.	Line 207-218

PRISMA 2020 Checklist

Section and Topic	Item #	Checklist item	Location where item is reported
**RESULTS**			
Study selection	16a	Describe the results of the search and selection process, from the number of records identified in the search to the number of studies included in the review, ideally using a flow diagram.	Line 254-257
	16b	Cite studies that might appear to meet the inclusion criteria, but which were excluded, and explain why they were excluded.	Additional file 3: figure S1
Study characteristics	17	Cite each included study and present its characteristics.	Table 3
Risk of bias in studies	18	Present assessments of risk of bias for each included study.	Additional file 4
Results of individual studies	19	For all outcomes, present, for each study: (a) summary statistics for each group (where appropriate) and (b) an effect estimate and its precision (e.g. confidence/credible interval), ideally using structured tables or plots.	Table 4 and Additional file 3
Results of syntheses	20a	For each synthesis, briefly summarise the characteristics and risk of bias among contributing studies.	Line 258-268
	20b	Present results of all statistical syntheses conducted. If meta-analysis was done, present for each the summary estimate and its precision (e.g. confidence/credible interval) and measures of statistical heterogeneity. If comparing groups, describe the direction of the effect.	Table 4 and Additional file 3
	20c	Present results of all investigations of possible causes of heterogeneity among study results.	Line 320-321, 347-350
	20d	Present results of all sensitivity analyses conducted to assess the robustness of the synthesized results.	Additional file 3
Reporting biases	21	Present assessments of risk of bias due to missing results (arising from reporting biases) for each synthesis assessed.	Additional file 4
Certainty of evidence	22	Present assessments of certainty (or confidence) in the body of evidence for each outcome assessed.	Table 4 and Additional file 3
**DISCUSSION**			
Discussion	23a	Provide a general interpretation of the results in the context of other evidence.	Line 303-305
	23b	Discuss any limitations of the evidence included in the review.	Line 411-426
	23c	Discuss any limitations of the review processes used.	Line 411-426
	23d	Discuss implications of the results for practice, policy, and future research.	Line 314-408
**OTHER INFORMATION**			
Registration and protocol	24a	Provide registration information for the review, including register name and registration number, or state that the review was not registered.	Register ongoing
	24b	Indicate where the review protocol can be accessed, or state that a protocol was not prepared.	Protocol preparing
	24c	Describe and explain any amendments to information provided at registration or in the protocol.	/
Support	25	Describe sources of financial or non-financial support for the review, and the role of the funders or sponsors in the review.	Not applicable
Competing interests	26	Declare any competing interests of review authors.	Line 464
Availability of data, code and other materials	27	Report which of the following are publicly available and where they can be found: template data collection forms; data extracted from included studies; data used for all analyses; analytic code; any other materials used in the review.	Line 467-469

From: Page MJ, McKenzie JE, Bossuyt PM, Boutron I, Hoffmann TC, Mulrow CD, et al. The PRISMA 2020 statement: an updated guideline for reporting systematic reviews. BMJ 2021;372:n71. doi: 10.1136/bmj.n71

Search terms and strategy

Summary of search terms and strategy in English databases

1. ASD AND HHVs

PubMed: 441

Web of Science: 230

("Autistic Disorder" or "Disorder, Autistic” or “Disorders, Autistic" or "Autism” or “Autism, Early Infantile" or "Early Infantile Autism” or “Infantile Autism, Early" or "Autism, Infantile" or "Infantile Autism" or "Kanner's Syndrome" or "Kanners Syndrome" or "Kanner Syndrome" or "Autism Spectrum Disorder" or" Autistic Spectrum Disorder" or "Autistic Spectrum Disorders" or "Disorder, Autistic Spectrum" or "Autism Spectrum Disorders") AND ("HSV" or "Simplexvirus" or "Simplexviruses" or "Herpes Simplex Virus" or "Herpes Simplex Viruses" or "Herpes Labialis Virus" or "Herpes Labialis Viruses" or "Labialis Viruses, Herpes" or "Labialis Virus, Herpes" or "Viruses, Herpes Labialis" or "Virus, Herpes Labialis" or "Herpesvirus Hominis" or "Herpesvirus Homini" or "Homini, Herpesvirus" or "Hominis, Herpesvirus" or "Herpesvirus 1, Saimiriine" or "Marmoset Virus" or "Marmoset Viruses" or "Marmoset Herpesvirus" or "Herpesviruses, Marmoset" or "Herpesvirus, Marmoset" or "Marmoset Herpesviruses" or "Herpesvirus 1 (alpha), Saimirine" or "Herpesvirus 1, Saimirine" or "Saimirine Herpesvirus 1" or "Herpesvirus Platyrhinae" or "Platyrhinae, Herpesvirus" or "Herpes-T Virus" or "Herpes T Virus" or "Herpes-T Viruses" or "Saimiriine Herpesvirus 1" or "Herpesvirus 16, Cercopithecine" or "Herpesvirus Papio 2" or "Cercopithecine Herpesvirus 16" OR "VZV" or "Herpesvirus 3, Human" or "Herpesvirus Varicellae" or "Varicellae, Herpesvirus" or "Human Herpesvirus 3" or "Chickenpox Virus" or "Chickenpox Viruses" or "Herpes zoster Virus" or "Herpes zoster Viruses" or "Ocular Herpes zoster Virus" or "Varicella-Zoster Virus" or "Varicella Zoster Virus" or "Varicella-Zoster Viruses" or "Herpesvirus 3 (alpha), Human" or "VZ Virus" or "VZ Viruses" or "HHV-3" OR "Herpesvirus 4, Human" or "Burkitt Herpesvirus" or "Herpesvirus, Burkitt" or "Burkitt Lymphoma Virus" or "Lymphoma Virus, Burkitt or Burkitt's Lymphoma Virus" or "Burkitts Lymphoma Virus" or "E-B Virus" or "E-B Virus" or "E-B Viruses" or "EBV" or "Epstein-Barr Virus" or "Epstein Barr Virus" or "Herpesvirus 4 (gamma), Human" or "HHV-4" or "Human Herpesvirus 4" or "Infectious Mononucleosis Virus" or "Infectious Mononucleosis Viruses" or "Mononucleosis Viruses, Infectious" or "Mononucleosis Virus, Infectious" or "Epstein-Barr Virus Infections" or "Epstein Barr Virus Infections" or "Infections, EBV" or "EBV Infection" or "Infection, EBV" or "EBV Infections" or "Human Herpes Virus 4 Infections" or "Human Herpesvirus 4 Infections" or "Herpesvirus 4 Infections, Human" or "Epstein-Barr Virus Infection" or "Epstein Barr Virus Infection" or "Infection, Epstein-Barr Virus" or "Virus Infection, Epstein-Barr" or "Virus Infections, Epstein-Barr" or "Infections, Epstein-Barr Virus" OR "CMV" or "Cytomegalovirus Infections" or "Cytomegalovirus Infection" or "Infection, Cytomegalovirus" or "Cytomegalovirus Inclusion Disease" or "Cytomegalovirus Inclusion Diseases" or "Disease, Cytomegalovirus Inclusion" or "Diseases, Cytomegalovirus Inclusion" or "Inclusion Disease, Cytomegalovirus" or "Inclusion Diseases, Cytomegalovirus" or "Cytomegalic Inclusion Disease" or "Cytomegalic Inclusion Diseases" or "Disease, Cytomegalic Inclusion" or "Inclusion Disease, Cytomegalic" or

"Inclusion Disease" or "Inclusion Diseases" or "Infections, Cytomegalovirus" or "Salivary Gland Virus Disease" or "Cytomegalovirus Colitis" or "Colitis, Cytomegalovirus" or "Cytomegalovirus Colitides" or "Severe Cytomegalovirus Infection" or "CytoCMVmegalovirus Infection, Severe" or "Cytomegalovirus Infections, Severe" or "Infection, Severe Cytomegalovirus" or "Severe Cytomegalovirus Infections" or "Renal Tubular Cytomegalovirus Inclusions" or "Renal Tubular Cytomegalovirus Inclusion" or "Cytomegalovirus Inclusion" or "Inclusion, Cytomegalovirus" or "Cytomegalovirus Inclusions" or "CMV Inclusions" or "Inclusion, CMV" or "CMV Inclusion" or "Congenital Cytomegalovirus Infection" or "Congenital Cytomegalovirus Infections" or "Cytomegalovirus Infection, Congenital" or "Infection, Congenital Cytomegalovirus" or "Congenital CMV Infection" or "CMV Infection, Congenital" or "Congenital CMV Infections" or "Infection, Congenital CMV" or "Perinatal Cytomegalovirus Infection" or "Cytomegalovirus Infection, Perinatal" or "Infection, Perinatal Cytomegalovirus" or "Perinatal Cytomegalovirus Infections" or "Perinatal CMV Infection" or "CMV Infection, Perinatal" or "Infection, Perinatal CMV" or "Perinatal CMV Infections" or "Cytomegalovirus" or "Cytomegaloviruses" or "Salivary Gland Viruses" or "Salivary Gland Virus" or "Viruses, Salivary Gland" or "Virus, Salivary Gland" or "Herpesvirus 5, Human" or "Herpesvirus 5 (beta), Human" or "HHV 5" or "Human Herpesvirus 5" OR "Herpesvirus 6, Human" or "HBLV" or "HHV-6" or "Human B-Lymphotropic Virus" or "B-Lymphotropic Viruses, Human" or "B-Lymphotropic Virus, Human" or "Human B Lymphotropic Virus" or "Human B-Lymphotropic Viruses" or "HHV6" or "Human Herpesvirus 6" or "Human betaherpesvirus 6" or "Human betaherpesvirus 6s" or "Herpesvirus 6A, Human" or "Human Herpesvirus 6A" or "HHV-6A" or "HHV6A" or "Human betaherpesvirus 6A" or "Herpesvirus 6B, Human" or "Human Herpesvirus 6B" or "HHV-6B" or "HHV6B" or "Human betaherpesvirus 6B" or "Chromosomally Integrated Human Herpesvirus 6" or "ciHHV-6" or "ciHHV6" or "Chromosomally Integrated Human Herpesvirus 6A" or "ciHHV-6A" or "ciHHV6A" or "Chromosomally Integrated Human Herpesvirus 6B" or "ciHHV-6B" or "ciHHV6B" or "Inherited Chromosomally Integrated Human Herpesvirus 6" or "iciHHV6" or "iciHHV-6" or "Inherited Chromosomally Integrated Human Herpesvirus 6A" or "iciHHV-6A" or "iciHHV6A" or "Inherited Chromosomally Integrated Human Herpesvirus 6B" or "iciHHV-6B" or "iciHHV6B" OR "Herpesvirus 7, Human" or "Human Herpesvirus 7" or "HHV-7" OR "Herpesvirus 8, Human" or "Human Herpesvirus 8" or "HHV-8" or "Herpesvirus, Kaposi's Sarcoma-Associated" or "Herpesviruses, Kaposi's Sarcoma-Associated" or "Herpesvirus, Kaposi's Sarcoma Associated" or "Herpesvirus, Kaposis Sarcoma-Associated" or "Kaposi's Sarcoma-Associated Herpesviruses" or "Sarcoma-Associated Herpesviruses, Kaposi's" or "Kaposi's Sarcoma-Associated Herpesvirus" or "Kaposi's Sarcoma Associated Herpesvirus" or "Kaposis Sarcoma-Associated Herpesvirus" or "Kaposi Sarcoma-Associated Herpesvirus" or "Kaposi Sarcoma Associated Herpesvirus" or "Sarcoma-Associated Herpesvirus, Kaposi" or "Herpesvirus, Kaposi Sarcoma-Associated" or "Herpesvirus, Kaposi Sarcoma Associated" or "KSHV")

2. ADHD AND HHVs

PubMed: 207

Web of Science: 51

(“Attention Deficit Disorder with Hyperactivity“ or ”ADHD“ or ”ADDH“ or ”Attention Deficit Disorders with Hyperactivity“ or ”Attention Deficit Hyperactivity Disorders“ or “Attention Deficit Hyperactivity Disorder” or “Attention Deficit-Hyperactivity Disorder” or “Attention Deficit-Hyperactivity Disorders” or “Deficit-Hyperactivity Disorder, Attention” or “Deficit-Hyperactivity Disorders, Attention” or “Disorder, Attention Deficit-Hyperactivity” or “Disorders, Attention Deficit-Hyperactivity” or “Hyperkinetic Syndrome” or “Syndromes, Hyperkinetic” or “Attention Deficit Disorder” or “Attention Deficit Disorders” or “Deficit Disorder, Attention” or “Deficit Disorders, Attention” or “Disorder, Attention Deficit” or “Disorders, Attention Deficit” or “Brain Dysfunction, Minimal” or “Dysfunction, Minimal Brain” or “Minimal Brain Dysfunction”) AND ("HSV" or "Simplexvirus" or "Simplexviruses" or "Herpes Simplex Virus" or "Herpes Simplex Viruses" or "Herpes Labialis Virus" or "Herpes Labialis Viruses" or "Labialis Viruses, Herpes" or "Labialis Virus, Herpes" or "Viruses, Herpes Labialis" or "Virus, Herpes Labialis" or "Herpesvirus Hominis" or "Herpesvirus Homini" or "Homini, Herpesvirus" or "Hominis, Herpesvirus" or "Herpesvirus 1, Saimiriine" or "Marmoset Virus" or "Marmoset Viruses" or "Marmoset Herpesvirus" or "Herpesviruses, Marmoset" or "Herpesvirus, Marmoset" or "Marmoset Herpesviruses" or "Herpesvirus 1 (alpha), Saimirine" or "Herpesvirus 1, Saimirine" or "Saimirine Herpesvirus 1" or "Herpesvirus Platyrhinae" or "Platyrhinae, Herpesvirus" or "Herpes-T Virus" or "Herpes T Virus" or "Herpes-T Viruses" or "Saimiriine Herpesvirus 1" or "Herpesvirus 16, Cercopithecine" or "Herpesvirus Papio 2" or "Cercopithecine Herpesvirus 16" OR "VZV" or "Herpesvirus 3, Human" or "Herpesvirus Varicellae" or "Varicellae, Herpesvirus" or "Human Herpesvirus 3" or "Chickenpox Virus" or "Chickenpox Viruses" or "Herpes zoster Virus" or "Herpes zoster Viruses" or "Ocular Herpes zoster Virus" or "Varicella-Zoster Virus" or "Varicella Zoster Virus" or "Varicella-Zoster Viruses" or "Herpesvirus 3 (alpha), Human" or "VZ Virus" or "VZ Viruses" or "HHV-3" OR "Herpesvirus 4, Human" or "Burkitt Herpesvirus" or "Herpesvirus, Burkitt" or "Burkitt Lymphoma Virus" or "Lymphoma Virus, Burkitt or Burkitt's Lymphoma Virus" or "Burkitts Lymphoma Virus" or "E-B Virus" or "E-B Virus" or "E-B Viruses" or "EBV" or "Epstein-Barr Virus" or "Epstein Barr Virus" or "Herpesvirus 4 (gamma), Human" or "HHV-4" or "Human Herpesvirus 4" or "Infectious Mononucleosis Virus" or "Infectious Mononucleosis Viruses" or "Mononucleosis Viruses, Infectious" or "Mononucleosis Virus, Infectious" or "Epstein-Barr Virus Infections" or "Epstein Barr Virus Infections" or "Infections, EBV" or "EBV Infection" or "Infection, EBV" or "EBV Infections" or "Human Herpes Virus 4 Infections" or "Human Herpesvirus 4 Infections" or "Herpesvirus 4 Infections, Human" or "Epstein-Barr Virus Infection" or "Epstein Barr Virus Infection" or "Infection, Epstein-Barr Virus" or "Virus Infection, Epstein-Barr" or "Virus Infections, Epstein-Barr" or "Infections, Epstein-Barr Virus" OR "CMV" or "Cytomegalovirus Infections" or "Cytomegalovirus Infection" or "Infection, Cytomegalovirus" or "Cytomegalovirus Inclusion Disease" or "Cytomegalovirus Inclusion Diseases" or "Disease, Cytomegalovirus

Inclusion" or "Diseases, Cytomegalovirus Inclusion" or "Inclusion Disease, Cytomegalovirus" or "Inclusion Diseases, Cytomegalovirus" or "Cytomegalic Inclusion Disease" or "Cytomegalic Inclusion Diseases" or "Disease, Cytomegalic Inclusion" or "Inclusion Disease, Cytomegalic" or "Inclusion Disease" or "Inclusion Diseases" or "Infections, Cytomegalovirus" or "Salivary Gland Virus Disease" or "Cytomegalovirus Colitis" or "Colitis, Cytomegalovirus" or "Cytomegalovirus Colitides" or "Severe Cytomegalovirus Infection" or "CytoCMVmegalovirus Infection, Severe" or "Cytomegalovirus Infections, Severe" or "Infection, Severe Cytomegalovirus" or "Severe Cytomegalovirus Infections" or "Renal Tubular Cytomegalovirus Inclusions" or "Renal Tubular Cytomegalovirus Inclusion" or "Cytomegalovirus Inclusion" or "Inclusion, Cytomegalovirus" or "Cytomegalovirus Inclusions" or "CMV Inclusions" or "Inclusion, CMV" or "CMV Inclusion" or "Congenital Cytomegalovirus Infection" or "Congenital Cytomegalovirus Infections" or "Cytomegalovirus Infection, Congenital" or "Infection, Congenital Cytomegalovirus" or "Congenital CMV Infection" or "CMV Infection, Congenital" or "Congenital CMV Infections" or "Infection, Congenital CMV" or "Perinatal Cytomegalovirus Infection" or "Cytomegalovirus Infection, Perinatal" or "Infection, Perinatal Cytomegalovirus" or "Perinatal Cytomegalovirus Infections" or "Perinatal CMV Infection" or "CMV Infection, Perinatal" or "Infection, Perinatal CMV" or "Perinatal CMV Infections" or "Cytomegalovirus" or "Cytomegaloviruses" or "Salivary Gland Viruses" or "Salivary Gland Virus" or "Viruses, Salivary Gland" or "Virus, Salivary Gland" or "Herpesvirus 5, Human" or "Herpesvirus 5 (beta), Human" or "HHV 5" or "Human Herpesvirus 5" OR "Herpesvirus 6, Human" or "HBLV" or "HHV-6" or "Human B-Lymphotropic Virus" or "B-Lymphotropic Viruses, Human" or "B-Lymphotropic Virus, Human" or "Human B Lymphotropic Virus" or "Human B-Lymphotropic Viruses" or "HHV6" or "Human Herpesvirus 6" or "Human betaherpesvirus 6" or "Human betaherpesvirus 6s" or "Herpesvirus 6A, Human" or "Human Herpesvirus 6A" or "HHV-6A" or "HHV6A" or "Human betaherpesvirus 6A" or "Herpesvirus 6B, Human" or "Human Herpesvirus 6B" or "HHV-6B" or "HHV6B" or "Human betaherpesvirus 6B" or "Chromosomally Integrated Human Herpesvirus 6" or "ciHHV-6" or "ciHHV6" or "Chromosomally Integrated Human Herpesvirus 6A" or "ciHHV-6A" or "ciHHV6A" or "Chromosomally Integrated Human Herpesvirus 6B" or "ciHHV-6B" or "ciHHV6B" or "Inherited Chromosomally Integrated Human Herpesvirus 6" or "iciHHV6" or "iciHHV-6" or "Inherited Chromosomally Integrated Human Herpesvirus 6A" or "iciHHV-6A" or "iciHHV6A" or "Inherited Chromosomally Integrated Human Herpesvirus 6B" or "iciHHV-6B" or "iciHHV6B" OR "Herpesvirus 7, Human" or "Human Herpesvirus 7" or "HHV-7" OR "Herpesvirus 8, Human" or "Human Herpesvirus 8" or "HHV-8" or "Herpesvirus, Kaposi's Sarcoma-Associated" or "Herpesviruses, Kaposi's Sarcoma-Associated" or "Herpesvirus, Kaposi's Sarcoma Associated" or "Herpesvirus, Kaposis Sarcoma-Associated" or "Kaposi's Sarcoma-Associated Herpesviruses" or "Sarcoma-Associated Herpesviruses, Kaposi's" or "Kaposi's Sarcoma-Associated Herpesvirus" or "Kaposi's Sarcoma Associated Herpesvirus" or "Kaposis Sarcoma-Associated Herpesvirus" or "Kaposi Sarcoma-Associated Herpesvirus" or "Kaposi Sarcoma Associated Herpesvirus" or "Sarcoma-Associated Herpesvirus, Kaposi" or "Herpesvirus, Kaposi Sarcoma-Associated" or "Herpesvirus, Kaposi Sarcoma Associated" or "KSHV")

3. TD/TS AND HHVs

PubMed: 120

Web of Science: 12

(“Tourette Syndrome” or “Syndrome, Tourette” or “Multiple Motor and Vocal Tic Disorder, Combined” or “Tic Disorder, Combined Vocal and Multiple Motor“ or ”Tourette Disease“ or “Tourette Disorder” or “Tourette's Disease” or “Tourettes Disease” or “Tourette's Disorder” or “Tourettes Disorder” or “Tourette's Syndrome” or “Tourettes Syndrome” or “Chronic Motor and Vocal Tic Disorder” or “Gilles De La Tourette's Syndrome” or “Gilles de la Tourette Disorder” or “Combined Multiple Motor and Vocal Tic Disorder” or “Combined Vocal and Multiple Motor Tic Disorder” or “Gilles de la Tourette Syndrome” or “Gilles de la Tourette's Disease” or “Tic Disorders” or “Tic Disorder” or “Transient Tic Disorder” or “Transient Tic Disorders” or “Vocal Tic Disorder” or “Vocal Tic Disorder” or “Vocal Tic Disorders” or “Childhood Tic Disorders” or “Childhood Tic Disorder” or “Motor Tic Disorders” or “Motor Tic Disorder” or “Post-Traumatic Tic Disorder” or “Post Traumatic Tic Disorder” or “Post-Traumatic Tic Disorders”) AND ("HSV" or "Simplexvirus" or "Simplexviruses" or "Herpes Simplex Virus" or "Herpes Simplex Viruses" or "Herpes Labialis Virus" or "Herpes Labialis Viruses" or "Labialis Viruses, Herpes" or "Labialis Virus, Herpes" or "Viruses, Herpes Labialis" or "Virus, Herpes Labialis" or "Herpesvirus Hominis" or "Herpesvirus Homini" or "Homini, Herpesvirus" or "Hominis, Herpesvirus" or "Herpesvirus 1, Saimiriine" or "Marmoset Virus" or "Marmoset Viruses" or "Marmoset Herpesvirus" or "Herpesviruses, Marmoset" or "Herpesvirus, Marmoset" or "Marmoset Herpesviruses" or "Herpesvirus 1 (alpha), Saimirine" or "Herpesvirus 1, Saimirine" or "Saimirine Herpesvirus 1" or "Herpesvirus Platyrhinae" or "Platyrhinae, Herpesvirus" or "Herpes-T Virus" or "Herpes T Virus" or "Herpes-T Viruses" or "Saimiriine Herpesvirus 1" or "Herpesvirus 16, Cercopithecine" or "Herpesvirus Papio 2" or "Cercopithecine Herpesvirus 16" OR "VZV" or "Herpesvirus 3, Human" or "Herpesvirus Varicellae" or "Varicellae, Herpesvirus" or "Human Herpesvirus 3" or "Chickenpox Virus" or "Chickenpox Viruses" or "Herpes zoster Virus" or "Herpes zoster Viruses" or "Ocular Herpes zoster Virus" or "Varicella-Zoster Virus" or "Varicella Zoster Virus" or "Varicella-Zoster Viruses" or "Herpesvirus 3 (alpha), Human" or "VZ Virus" or "VZ Viruses" or "HHV-3" OR "Herpesvirus 4, Human" or "Burkitt Herpesvirus" or "Herpesvirus, Burkitt" or "Burkitt Lymphoma Virus" or "Lymphoma Virus, Burkitt or Burkitt's Lymphoma Virus" or "Burkitts Lymphoma Virus" or "E-B Virus" or "E-B Virus" or "E-B Viruses" or "EBV" or "Epstein-Barr Virus" or "Epstein Barr Virus" or "Herpesvirus 4 (gamma), Human" or "HHV-4" or "Human Herpesvirus 4" or "Infectious Mononucleosis Virus" or "Infectious Mononucleosis Viruses" or "Mononucleosis Viruses, Infectious" or "Mononucleosis Virus, Infectious" or "Epstein-Barr Virus Infections" or "Epstein Barr Virus Infections" or "Infections, EBV" or "EBV Infection" or "Infection, EBV" or "EBV Infections" or "Human Herpes Virus 4 Infections" or "Human Herpesvirus 4 Infections" or "Herpesvirus 4 Infections, Human" or "Epstein-Barr Virus

Infection" or "Epstein Barr Virus Infection" or "Infection, Epstein-Barr Virus" or "Virus Infection, Epstein-Barr" or "Virus Infections, Epstein-Barr" or "Infections, Epstein-Barr Virus" OR "CMV" or "Cytomegalovirus Infections" or "Cytomegalovirus Infection" or "Infection, Cytomegalovirus" or "Cytomegalovirus Inclusion Disease" or "Cytomegalovirus Inclusion Diseases" or "Disease, Cytomegalovirus Inclusion" or "Diseases, Cytomegalovirus Inclusion" or "Inclusion Disease, Cytomegalovirus" or "Inclusion Diseases, Cytomegalovirus" or "Cytomegalic Inclusion Disease" or "Cytomegalic Inclusion Diseases" or "Disease, Cytomegalic Inclusion" or "Inclusion Disease, Cytomegalic" or "Inclusion Disease" or "Inclusion Diseases" or "Infections, Cytomegalovirus" or "Salivary Gland Virus Disease" or "Cytomegalovirus Colitis" or "Colitis, Cytomegalovirus" or "Cytomegalovirus Colitides" or "Severe Cytomegalovirus Infection" or "CytoCMVmegalovirus Infection, Severe" or "Cytomegalovirus Infections, Severe" or "Infection, Severe Cytomegalovirus" or "Severe Cytomegalovirus Infections" or "Renal Tubular Cytomegalovirus Inclusions" or "Renal Tubular Cytomegalovirus Inclusion" or "Cytomegalovirus Inclusion" or "Inclusion, Cytomegalovirus" or "Cytomegalovirus Inclusions" or "CMV Inclusions" or "Inclusion, CMV" or "CMV Inclusion" or "Congenital Cytomegalovirus Infection" or "Congenital Cytomegalovirus Infections" or "Cytomegalovirus Infection, Congenital" or "Infection, Congenital Cytomegalovirus" or "Congenital CMV Infection" or "CMV Infection, Congenital" or "Congenital CMV Infections" or "Infection, Congenital CMV" or "Perinatal Cytomegalovirus Infection" or "Cytomegalovirus Infection, Perinatal" or "Infection, Perinatal Cytomegalovirus" or "Perinatal Cytomegalovirus Infections" or "Perinatal CMV Infection" or "CMV Infection, Perinatal" or "Infection, Perinatal CMV" or "Perinatal CMV Infections" or "Cytomegalovirus" or "Cytomegaloviruses" or "Salivary Gland Viruses" or "Salivary Gland Virus" or "Viruses, Salivary Gland" or "Virus, Salivary Gland" or "Herpesvirus 5, Human" or "Herpesvirus 5 (beta), Human" or "HHV 5" or "Human Herpesvirus 5" OR "Herpesvirus 6, Human" or "HBLV" or "HHV-6" or "Human B-Lymphotropic Virus" or "B-Lymphotropic Viruses, Human" or "B-Lymphotropic Virus, Human" or "Human B Lymphotropic Virus" or "Human B-Lymphotropic Viruses" or "HHV6" or "Human Herpesvirus 6" or "Human betaherpesvirus 6" or "Human betaherpesvirus 6s" or "Herpesvirus 6A, Human" or "Human Herpesvirus 6A" or "HHV-6A" or "HHV6A" or "Human betaherpesvirus 6A" or "Herpesvirus 6B, Human" or "Human Herpesvirus 6B" or "HHV-6B" or "HHV6B" or "Human betaherpesvirus 6B" or "Chromosomally Integrated Human Herpesvirus 6" or "ciHHV-6" or "ciHHV6" or "Chromosomally Integrated Human Herpesvirus 6A" or "ciHHV-6A" or "ciHHV6A" or "Chromosomally Integrated Human Herpesvirus 6B" or "ciHHV-6B" or "ciHHV6B" or "Inherited Chromosomally Integrated Human Herpesvirus 6" or "iciHHV6" or "iciHHV-6" or "Inherited Chromosomally Integrated Human Herpesvirus 6A" or "iciHHV-6A" or "iciHHV6A" or "Inherited Chromosomally Integrated Human Herpesvirus 6B" or "iciHHV-6B" or "iciHHV6B" OR "Herpesvirus 7, Human" or "Human Herpesvirus 7" or "HHV-7" OR "Herpesvirus 8, Human" or "Human Herpesvirus 8" or "HHV-8" or "Herpesvirus, Kaposi's Sarcoma-Associated" or "Herpesviruses, Kaposi's Sarcoma-Associated" or "Herpesvirus, Kaposi's Sarcoma Associated" or "Herpesvirus, Kaposis Sarcoma-Associated" or "Kaposi's Sarcoma-Associated Herpesviruses" or "Sarcoma-Associated Herpesviruses, Kaposi's" or "Kaposi's Sarcoma-Associated Herpesvirus" or "Kaposi's Sarcoma Associated Herpesvirus" or "Kaposis Sarcoma-Associated Herpesvirus" or "Kaposi Sarcoma-Associated Herpesvirus" or "Kaposi Sarcoma Associated Herpesvirus" or "Sarcoma-Associated Herpesvirus, Kaposi" or

"Herpesvirus, Kaposi Sarcoma-Associated" or "Herpesvirus, Kaposi Sarcoma Associated" or "KSHV")

Summary of search terms and strategy in Chinese databases

Search terms in Chinese

1. ASD AND HHVs

检索结果：维普 3 篇 / 知网（CMV）6 篇 / 万方—布尔逻辑（CMV）15 篇

（孤独症儿童 + 孤独症谱系障碍 + 孤独症谱系 + 孤独症患儿 + 孤独症谱系障碍 +孤独症谱系障碍儿童 + 孤独症患者 + 高功能孤独症 + 婴幼儿孤独症 + 婴儿孤独症 + 克氏孤独症 + 小儿孤独症 + 自闭症儿童 + 自闭症谱系 + 自闭症谱系障碍 + 自闭症患儿 + 自闭症患者 + 自闭症谱系障碍儿） * （人疱疹病毒 + 人疱疹病毒 6 型 + 人疱疹病毒 7 型+人疱疹病毒 8 型 + 人疱疹病毒 6+ 人疱疹病毒 7 + 人疱疹病毒 8 + 单纯疱疹病毒 + 单纯疱疹病毒感染 + 单纯疱疹病毒 1 + 单纯疱疹病毒 1 型 + 单纯疱疹病毒 2 型 + 单纯疱疹病毒 2 + 人疱疹病毒 3 + 人疱疹病毒 3 型 + 带状疱疹病毒 + 水痘-带状疱疹病毒 + 人疱疹病毒 4 + 人疱疹病毒 4 型 + EB 病毒感染 + 爱泼斯坦-巴尔病毒 + 人疱疹病毒 5 + 人疱疹病毒 5 型 + 巨细胞病毒 + 巨细胞病毒感染 + 先天性巨细胞病毒 + 先天性巨细胞病毒感染）

(Children with autism + autism spectrum disorder + Autism spectrum + Children with autism spectrum disorder + High-functioning autism + infantile autism + Klinefelter autism + pediatric autism + Autism Spectrum) * (Human Herpesvirus + Human Herpesvirus Type 6 + Human Herpesvirus Type 7 + Human Herpesvirus Type 8 + Human Herpesvirus 6+ Human Herpesvirus 7 + Human Herpesvirus 8 + Herpes Simplex Virus + Herpes Simplex Virus Infection + Herpes Simplex Virus 1 + Herpes Simplex Virus Type 1 + Herpes Simplex Virus Type 2 + Herpes Simplex Virus 2 + Human Herpesvirus 3 + Human Herpesvirus Type 3 + Herpes zoster virus + Varicella-zoster virus + Human herpesvirus 4 + Human herpesvirus type 4 + Epstein-Barr virus + Human herpesvirus 5 + Human herpesvirus type 5 + Cytomegalovirus + Cytomegalovirus infection + Congenital cytomegalovirus + Congenital cytomegalovirus infection)

2. ADHD AND HHVs

检索结果：维普 1 篇 / 知网（CMV）3 篇 / 万方—布尔逻辑（CMV）9 篇

(注意缺陷多动障碍 + 注意缺陷多动障碍儿童 + 注意缺陷多动障碍患儿 + 注意缺陷与多动障碍 + 注意缺陷多动症 + 注意缺陷与多动 + 注意缺陷伴多动）*（人疱疹病毒 + 人疱疹病毒 6 型 + 人疱疹病毒 7 型+ 人疱疹病毒 8 型 + 人疱疹病毒 6+ 人疱疹病毒 7 + 人疱疹病毒 8 + 单纯疱疹病毒 + 单纯疱疹病毒感染 + 单纯疱疹病毒 1 + 单纯疱疹病毒 1 型 + 单纯疱疹病毒 2 型 + 单纯疱疹病毒 2 + 人疱疹病毒 3 + 人疱疹病毒 3 型 + 带状疱疹病毒 + 水痘-带状疱疹病毒 + 人疱疹病毒 4 + 人疱疹病毒 4 型 + EB 病毒感染 + 爱泼斯坦-巴尔病毒 + 人疱疹病毒 5 + 人疱疹病毒 5 型 + 巨细胞病毒 + 巨细胞病毒感染 + 先天性巨细胞病毒 + 先天性巨细胞病毒感染）

(Attention Deficit Hyperactivity Disorder + Children with Attention Deficit Hyperactivity Disorder + Attention Deficit and Hyperactivity Disorder + Attention Deficit and Hyperactivity + Attention Deficit with Hyperactivity) * (Human Herpesvirus + Human Herpesvirus Type 6 + Human Herpesvirus Type 7 + Human Herpesvirus Type 8 + Human Herpesvirus 6+ Human Herpesvirus 7 + Human Herpesvirus 8 + Herpes Simplex Virus + Herpes Simplex Virus Infection + Herpes Simplex Virus 1 + Herpes Simplex Virus Type 1 + Herpes Simplex Virus Type 2 + Herpes Simplex Virus 2 + Human Herpesvirus 3 + Human Herpesvirus Type 3 + Herpes zoster virus + Varicella-zoster virus + Human herpesvirus 4 + Human herpesvirus type 4 + Epstein-Barr virus + Human herpesvirus 5 + Human herpesvirus type 5 + Cytomegalovirus + Cytomegalovirus infection + Congenital cytomegalovirus + Congenital cytomegalovirus infection)

3. TD/TS AND HHVs

检索结果：维普 7 篇 / 知网（CMV）7 篇 / 万方—布尔逻辑（CMV）13 篇

(抽动症 + 抽动障碍 + 抽动秽语综合征 + 抽动秽语征 + 抽动症患儿 + 抽动障碍患儿 + 多发性抽动 + 发声性抽动 + 运动性抽动 + 多发性抽动综合征）*（人疱疹病毒 + 人疱疹病毒 6 型 + 人疱疹病毒 7 型+ 人疱疹病毒 8 型 + 人疱疹病毒 6+ 人疱疹病毒 7 + 人疱疹病毒 8 + 单纯疱疹病毒 + 单纯疱疹病毒感染 + 单纯疱疹病毒 1 + 单纯疱疹病毒 1 型 + 单纯疱疹病毒 2 型 + 单纯疱疹病毒 2 + 人疱疹病毒 3 + 人疱疹病毒 3 型 + 带状疱疹病毒 + 水痘-带状疱疹病毒 + 人疱疹病毒 4 + 人疱疹病毒 4 型 + EB 病毒感染 + 爱泼斯坦-巴尔病毒 + 人疱疹病毒 5 + 人疱疹病毒 5 型 + 巨细胞病毒 + 巨细胞病毒感染 + 先天性巨细胞病毒 + 先天性巨细胞病毒感染）

(Tourette's Syndrome + Tic Disorder + Tourette Syndrome + Tourette Signs + Children with Tourette's Syndrome + Children with Tourette's Disorder + Multiple Tics + Vocal Tics + Motor Tics + Multiple Tics Syndrome) * (Human Herpesvirus + Human Herpesvirus Type 6 + Human Herpesvirus Type 7 + Human Herpesvirus Type 8 + Human Herpesvirus 6+ Human Herpesvirus 7 + Human Herpesvirus 8 + Herpes Simplex Virus + Herpes Simplex Virus Infection + Herpes Simplex Virus 1 + Herpes Simplex Virus Type 1 + Herpes Simplex Virus Type 2 + Herpes Simplex Virus 2 + Human Herpesvirus 3 + Human Herpesvirus Type 3 + Herpes zoster virus + Varicella-zoster virus + Human herpesvirus 4 + Human herpesvirus type 4 + Epstein-Barr virus + Human herpesvirus 5 + Human herpesvirus type 5 + Cytomegalovirus + Cytomegalovirus infection + Congenital cytomegalovirus + Congenital cytomegalovirus infection)

Risk of bias assessments using the AHRQ evaluation criteria

Case-control Study (*retrospective cohort study)	Define the source of information (survey, record review)	List inclusion and exclusion criteria for exposed and unexposed subjects (cases and controls) or refer to previous publications	Indicate time period used for identifying patients	Indicate whether or not subjects were consecutive if not population-based	Indicate if evaluators of subjective components of study were masked to other aspects of the status of the participants	Describe any assessments undertaken for quality assurance purposes (e.g., test/retest of primary outcome measurements)	Explain any patient exclusions from analysis	Describe how confounding was assessed and/or controlled.	If applicable, explain how missing data were handled in the analysis	Summarize patient response rates and completeness of data collection	Clarify what follow-up, if any, was expected and the percentage of patients for which incomplete data or follow-up was obtained
Marylú Mora, 2009	yes	yes	yes	no	unclear	yes	yes	no	unclear	yes	no
Carla Lintas, 2010	yes	yes	no	no	unclear	yes	yes	no	unclear	yes	no
Jianhua Li, 2010	yes	unclear	yes	no	unclear	yes	yes	no	unclear	yes	no
Ivan Gentile, 2014 [a]	yes	yes	yes	no	unclear	yes	yes	yes	unclear	yes	no
Emanuela Zappulo, 2018	yes	yes	yes	no	unclear	yes	yes	no	unclear	yes	no
Thayne L. Sweeten, 2019	yes	yes	yes	no	unclear	unclear	yes	no	unclear	yes	no
Dmitry Maltsev, 2024	yes	yes	yes	no	unclear	yes	yes	no	unclear	yes	no
Ivan Gentile, 2014 [b]	yes	yes	yes	no	unclear	yes	yes	yes	unclear	yes	no
Ivan Gentile, 2017 [c]	yes	yes	yes	no	unclear	yes	yes	yes	unclear	yes	no
Maha I S Kawashti, 2006	yes	yes	no	no	unclear	yes	yes	no	unclear	yes	no
Ivan Gentile, 2014 [d]	yes	yes	yes	no	unclear	yes	yes	yes	unclear	yes	no
Ayako Sakamoto, 2015	yes	yes	yes	no	unclear	yes	unclear	no	unclear	yes	no

Mona-Lisa Engman, 2015	yes	unclear	yes	no	unclear	yes	yes	no	unclear	yes	no
Ivan Gentile, 2017 [e]	yes	yes	yes	no	unclear	yes	yes	yes	unclear	yes	no
Marjolein J Korndewal,2017*	yes	unclear	yes	no	unclear	yes	yes	no	yes	yes	yes
Chien-Heng Lin, 2021*	yes	unclear	yes	no	unclear	yes	yes	yes	unclear	yes	yes
Zeinab R Hassan, 2023	yes	unclear	no	no	unclear	yes	yes	no	unclear	yes	no
Garth L Nicolson, 2007	yes	unclear	no	no	unclear	yes	yes	yes	unclear	yes	no
Ivan Gentile, 2013	yes	yes	yes	no	unclear	yes	yes	yes	unclear	yes	no
Mervan Bekdas, 2014	yes	yes	yes	no	unclear	yes	yes	no	unclear	yes	no
Ruizhen Chai, 2005	yes	unclear	yes	no	unclear	no	no	no	unclear	yes	yes
Jiangyu Chen, 2012	yes	unclear	yes	no	unclear	yes	yes	no	unclear	yes	no
Yanzhao Chen, 2017	yes	yes	yes	no	unclear	yes	yes	no	unclear	yes	no
Jialin Xu, 2020	yes	no	yes	no	unclear	no	no	no	unclear	yes	no
Guifang Kuang, 2001	yes	unclear	no	no	unclear	yes	yes	no	unclear	yes	no
Guifang Kuang, 2005	yes	unclear	yes	no	unclear	yes	yes	no	unclear	yes	no
Yanhui Chen, 2006	yes	unclear	no	no	unclear	yes	yes	no	unclear	yes	no

[a] Title: Prevalence of herpes simplex virus 1 and 2 antibodies in patients with autism spectrum disorders.

[b] Title: Exposure to Varicella Zoster Virus is higher in children with autism spectrum disorder than in healthy controls. Results from a case-control study.

[c] Title: No evidence of congenital varicella zoster virus infection assessed through dried blood spot in children with autism spectrum disorders

[d] Title: Prevalence and titre of antibodies to cytomegalovirus and epstein-barr virus in patients with autism spectrum disorder.

[e] Title: Prevalence of Congenital Cytomegalovirus Infection Assessed Through Viral Genome Detection in Dried Blood Spots in Children with Autism Spectrum Disorders.

Quality assessments using the Newcastle-Ottawa Scale (NOS)

Case-control Study (*retrospective cohort study)	Selection				Comparability Control for important factor [a]	Exposure			Total score
	Adequate definition of cases	Representativeness of the cases	Selection of controls	Definition of controls		Ascertainment of exposure	Same method of ascertainment for cases and controls	Nonresponse rate	
Marylú Mora, 2009	★	★	★	★	★	-	★	★	7
Carla Lintas, 2010	★	-	★	★	★	-	★	★	6
Jianhua Li, 2010	★	★	-	★	★	-	★	★	6
Ivan Gentile, 2014 [b]	★	★	-	★	★	-	★	★	6
Emanuela Zappulo, 2018	★	★	★	★	★	-	★	★	7

Thayne L. Sweeten, 2019	★	★	★	★	★	-	★	★	7
Dmitry Maltsev, 2024	★	★	-	★	★	-	★	★	6
Ivan Gentile, 2014 [c]	★	★	-	★	★	-	★	★	6
Ivan Gentile, 2017[d]	★	★	-	★	★	-	★	★	6
Maha I S Kawashti, 2006	★	★	★	★	★	-	★	★	7
Ivan Gentile, 2014 [e]	★	★	-	★	★	-	★	★	6
Ayako Sakamoto, 2015	★	★	-	★	★	-	★	-	5

Mona-Lisa Engman, 2015	★	-	★	★	★	-	★	★	6
Ivan Gentile, 2017[f]	★	★	-	★	★	★	★	★	7
Marjolein J Korndewal, 2017*	★	★	★	★	★	★	★	★	8
Chien-Heng Lin, 2021*	★	★	★	★	★	★	★	★	8
Zeinab R Hassan, 2023	★	★	★	★	-	-	★	★	6
Garth L Nicolson, 2007	★	★	★	★	★	★	★	★	8
Ivan Gentile, 2013	★	★	-	★	★	-	★	★	6

Mervan Bekdas, 2014	★	★	★	★	★	★	★	★	8
Ruizhen Chai, 2005	★	★	★	-	★	-	★	★	6
Jiangyu Chen, 2012	★	★	★	★	★	-	★	★	7
Yanzhao Chen, 2017	★	★	-	★	★	-	★	★	6
Jialin Xu, 2020	★	★	-	★	★	-	★	★	6
Guifang Kuang, 2001	★	★	-	★	★	-	★	★	6
Guifang Kuang, 2005	★	★	-	★	★	-	★	★	6

Yanhui Chen, 2006	★	-	★	★	★	-	★	★	6

[a] A maximum of 2 stars can be allotted in this category.

[b] Title: Prevalence of herpes simplex virus 1 and 2 antibodies in patients with autism spectrum disorders.

[c] Title: Exposure to Varicella Zoster Virus is higher in children with autism spectrum disorder than in healthy controls. Results from a case-control study.

[d] Title: No evidence of congenital varicella zoster virus infection assessed through dried blood spot in children with autism spectrum disorders

[e] Title: Prevalence and titre of antibodies to cytomegalovirus and epstein-barr virus in patients with autism spectrum disorder.

[f] Title: Prevalence of Congenital Cytomegalovirus Infection Assessed Through Viral Genome Detection in Dried Blood Spots in Children with Autism Spectrum Disorders
